# Self-regulated optomechanics achieves ultrabroadband and high-efficiency all-optical devices

**DOI:** 10.1016/j.xinn.2026.101348

**Published:** 2026-03-12

**Authors:** Xuning Wang, Guodong Hou, Feiyu Zhang, Feihong Du, Ruhong Luo, Zhenhua Ma, Yezhan Lin, Chunyu Wang, Yifan Zhao, Binzhou Sun, Cenling Huang, Lu Yu, Shanyu Zheng, Qiang Li, Donglin Han, Haixin Qiu, Jiang Zou, Xin Chen, Tiannan Yang, Wei Li, Weihong Zhu, Xiangyang Zhu, Di Zhang, Yanqing Lu, Guang Meng, Xiaoshi Qian

**Affiliations:** 1 State Key Laboratory of Mechanical System and Vibration, Interdisciplinary Research Center, School of Mechanical Engineering, Shanghai Jiao Tong University, Shanghai 200240, China; 2 MOE Key Laboratory for Power Machinery and Engineering, School of Mechanical Engineering, Shanghai Jiao Tong University, Shanghai 200240, China; 3 Shanghai Key Laboratory of Functional Materials Chemistry, Key Laboratory for Advanced Materials and Institute of Fine Chemicals, East China University of Science and Technology, Shanghai 200237, China; 4 State Key Laboratory of Metal Matrix Composites, School of Materials Science and Engineering, Shanghai Jiao Tong University, Shanghai 200240, China; 5 National Laboratory of Solid State Microstructures, Collaborative Innovation Center of Advanced Microstructures, and College of Engineering and Applied Sciences, Nanjing University, Nanjing 210093, China; 6 Shanghai Academy of Natural Sciences (SANS), Shanghai 200031, China

**Keywords:** all-optical devices, photothermal responsive materials, self-regulated, soft actuator

## Abstract

All-optical logic devices require nonreciprocal wave propagation, which is often achieved by resonance or nonlinear effects at the cost of limited transmitted modes, wavelengths, and overall energy efficiency. By leveraging the inherited feedback loops, the nature-inspired self-regulated soft actuators could act as “gatekeepers” to identify the wave propagation direction to achieve similar logic functions in linear optics. Here, we report an effective design for all-optical diodes, transistors, and logic gates using self-regulated soft waveguides. These soft optomechanical systems, which are fabricated from hydrogels and liquid crystal elastomers (LCEs), exhibit direction recognition, autonomous alignment and selective transmission, achieving wavelength- and polarization-independent processing of signals and high overall efficiency. This versatile platform presents extended capabilities of integrated soft robotics and embodied intelligent material systems.

## Public summary

• Soft waveguides with self-regulated deformation under different illuminations.
• Wavelength and polarization independent all-optical diodes and transistors.
• Modular logic gates implement essential Boolean operations.

## Graphical abstract

**Figure:**
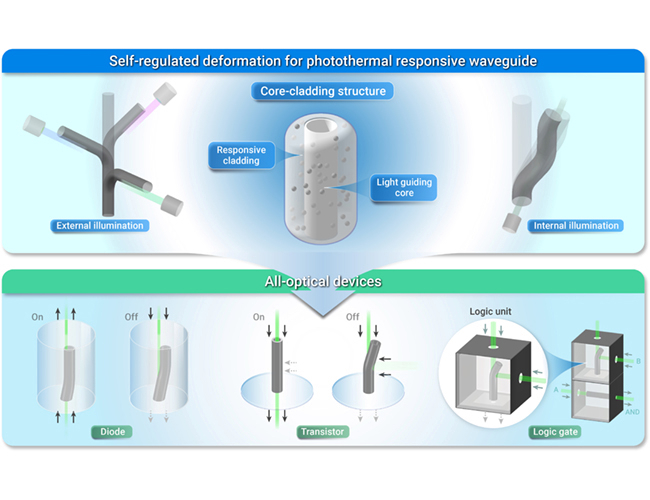

## Introduction

**Figure 1:**
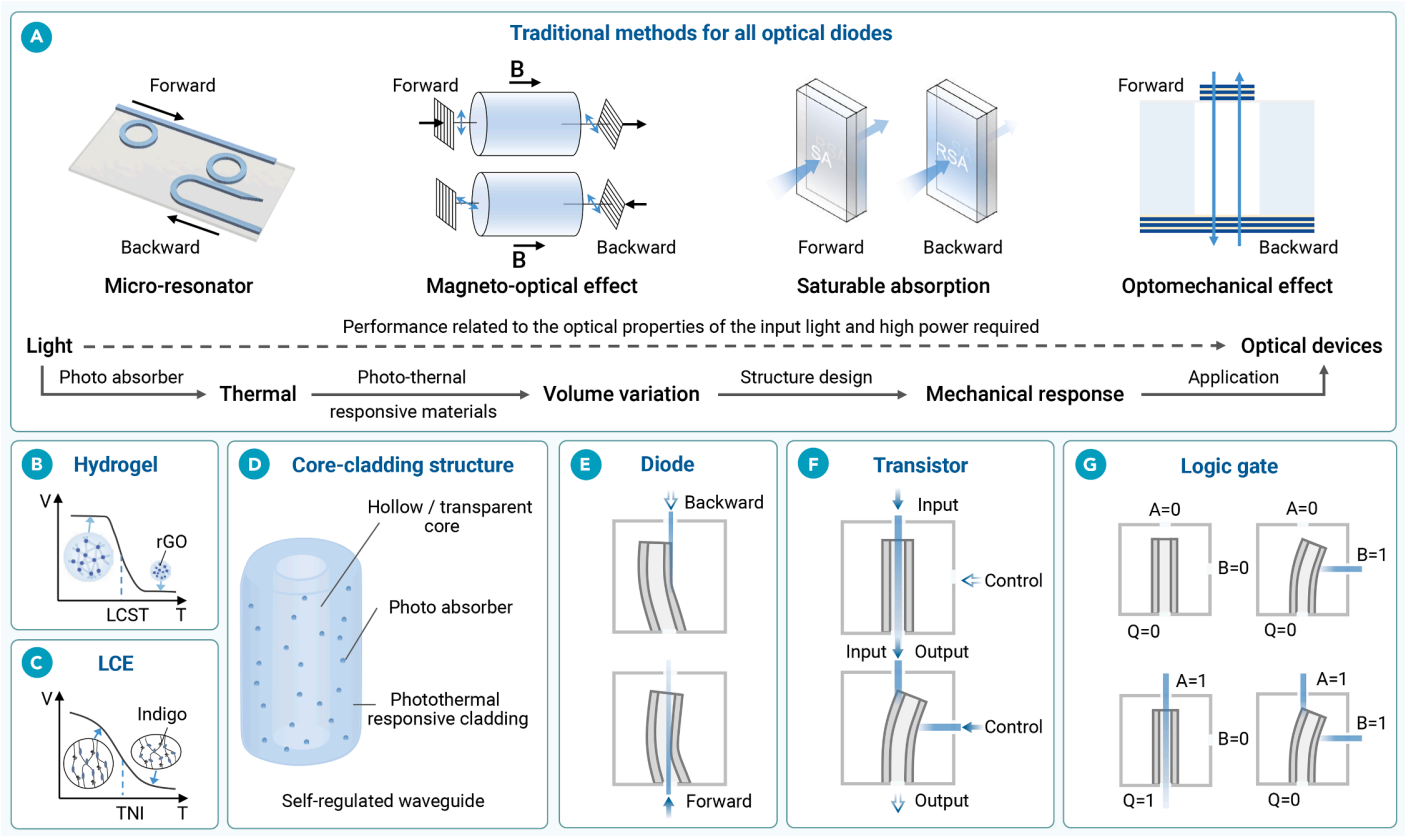
All-optical devices based on traditional methods and self-regulated photothermal-responsive waveguides (A) Traditional methods for all-optical diodes.^7^^,^^8^^,^^9^^,^^14^ (B) Thermally responsive behavior of PNIPAAm hydrogels. (C) Thermally responsive behavior of LCEs. (D) Structure of the responsive waveguide consisting of an actuating cladding with photoabsorbers inserted and the inside core is hollow or filled with transparent unresponsive materials. (E) Concept and structure of an optical diode, which allows light to pass in one direction, with inconsistent deformation of the responsive waveguide under illumination from different directions. (F) Concept and structure of an optical transistor, which can modulate strong input light with weaker control light, analogous to the source and gate functions in an electrical transistor. (G) Concept and structure of the basic element for logic gates, with response results under four different input combinations.

Modern society is built on advances in silicon-based electronics that process information by accurately controlling charge carriers^1^ in tightly engineered structures forming diodes, transistors, etc.^2^ All-optical devices, which modulate photons instead of electrons and holes, avoiding charge transport, thus preventing Joule heating and excessive energy loss^3^ are vital for communication, sensing, and detection.^4^^,^^5^^,^^6^ These all-optical approaches rely on nonlinear effects^7^^,^^8^^,^^9^^,^^10^^,^^11^^,^^12^^,^^13^ and symmetry breaking structures^14^^,^^15^ (Figure 1A) that highly depend on specific optical properties, such as the wavelength^16^^,^^17^^,^^18^^,^^19^ and polarization state,^20^^,^^21^^,^^22^ leading to narrow working bandwidth and low adaptability. Furthermore, the energy dissipation in the phase matching and coupling process^23^ is non-negligible, resulting in reduced overall efficiency.

Natural systems cannot afford this luxury. With inherited sensing, actuation, and feedback loops, organisms have evolved collective self-regulated behaviors, i.e., heliotropism,^24^^,^^25^ chemotropism,^26^ etc., to efficiently interact with nearby energies, regardless of the form, mode, or frequency. Similar ideas have been realized in stimuli-responsive material systems.^27^^,^^28^^,^^29^^,^^30^ Among these materials, photothermal-responsive materials stand out for their ability to convert light energy into heat via photoabsorbers.^31^ This conversion drives temperature-induced volume changes in the materials, enabling a range of mechanical responses, such as bending,^32^^,^^33^ oscillation,^34^^,^^35^ locomotion,^36^^,^^37^^,^^38^^,^^39^^,^^40^ nonreciprocal motion,^41^^,^^42^ and multimodal auto-switching functions.^43^ Photothermal-responsive materials can operate across diverse light wavelengths by integrating efficient broadband photoabsorbers.^44^ Recent advancements have demonstrated their remarkable sensitivity, enabling actuation under extremely low power inputs, even below the intensity of natural sunlight.^35^ Advanced 3D printing techniques have enabled fabrication and control of microscale-responsive joints,^45^ leading to miniaturized and highly integrated soft devices. These achievements collectively suggest possible routes to achieve self-regulated optical devices by utilizing multi-physical, highly efficient light-matter interactions.

Here, we introduce a universal design principle for self-regulated all-optical devices, including diodes, transistors, and logic gates, leveraging photothermal-responsive soft actuators. This principle is verified with actuators fabricated from poly(N-isopropylacrylamide) (PNIPAAm) hydrogels and LCEs. Reduced graphene oxides (rGOs) and Indigo are used as broadband photoabsorbers for PNIPAAm hydrogels (Figure 1B) and LCEs (Figure 1C), respectively, enabling the materials to respond to illumination with a broad range of wavelengths, and the two kinds of materials can work in different operating environments. A core-cladding structure is introduced (Figure 1D), with the photothermal-responsive cladding reacting to illumination on its outer surface or inner surface and the transparent core transmitting light. The self-regulated, omnidirectional deformation forms the basis for several device architectures: (1) an all-optical diode (Figure 1E) that achieves a high isolation ratio of up to 34.99 ± 0.94 dB, comparable with that achieved with traditional methods, for example, microresonators,^7^ but with an ultrawide spectral range covering the UV-visible-NIR bands; (2) a transistor (Figure 1F) comprising a gate that allows weak light to control strong light, with a theoretical gain of more than 57, whereas, experimentally, we achieved a 5× gain without high energy loss induced by nonlinear effects such as second harmonic generation^46^; and (3) modular logic gates (Figure 1G) implementing essential Boolean operations. These devices are effective, are independent of wavelength and polarization, and require no additional complicated structural design. Naturally, this design principle can be extended to a variety of stimuli-responsive materials, offering a universal platform for designing logic devices that respond to other forms of emitted energy.

## Materials and methods

### Sample fabrication

The precursor solution for PNIPAAm hydrogel prepared at room temperature was prepared with 30 wt % NIPAAm monomer, 1.5 wt % crosslinker BIS, and 1 vol % photo-initiator Darocur 1173 in DMSO with 0.3 wt % GO. The mixture was injected into a mold with tubular space inside, and cured under UV illumination. The cured hydrogel was immersed into DI water for full water exchange to remove unreacted monomers and DMSO. The tubular sample was put into 0.33 M hydrazine aqueous solution to reduce GO to rGO.

The precursor solution of PNIPAAm hydrogel prepared by the ice templet method was prepared with 20 wt % NIPAAm monomer, 1.5 wt % crosslinker BIS, 3.36 wt % photo-initiator Irgacure 2959 in DI water with 0.3 wt % GO. The mixture was injected into the molds and the molds were placed into a refrigerator at −18°C overnight. While the samples were frozen completely, they were cured with a UV lamp at −18°C. The cured hydrogels were immersed into DI water for full water exchange and then put into 0.33 M hydrazine aqueous solution to reduce GO to rGO.

The double-layer sample was assembled with a polyacrylamide (PAM) inner and a PNIPAAm tube. The PAM hydrogel precursor solution was prepared with 25 wt % AM monomer, 1.5 vol % crosslinker BIS, 1 vol % photo-initiator Irgacure in DI water. The mixture solution was injected into the mold and cured under UV illumination. The PAM cylinder and the PNIPAAm tube were hitched together to establish the double-layer structure.

The LCE precursor solution was prepared with 0.4 g RM257, 20 mg Irgacure 2959, 4 mg Indigo, 20 μL PETMP, 86 μL EDDET, and 8 μL DPA dissolved in dichloromethane. The mixture was ultrasonic dispersed for 10 min, then placed in a vacuum environment to eliminate air bubbles. The mixture solution was added into the mold, standing for 24 h to evaporate the solvent and complete the reaction. After the LCE tube was removed from the mold, the sample was stretched 2 times of initial length, and was cured under UV light for 4 h to fix the LCE sample in mono-domain state.

### Characterization of optical diode

The photothermal actuators used for optical diodes were made from PNIPAAm hydrogel prepared at room temperature, PNIPAAm hydrogel prepared with the ice template method, and LCE. The diode was irradiated by a 532 nm continuous wave laser (MGL-N-532A-5W), with a beam diameter of 1 mm. The output power of the diode was detected by a photodiode power sensor (S121C, Thorlabs) with a console (PM100USB, Thorlabs).

### Characterization of optical transistor

The photothermal actuator used for optical transistor was made from PNIPAAm hydrogel prepared by the ice templet method and the PAM inner layer was selected for comparison. The constant irradiation for the transistor was caused by a 532 nm continuous wave laser (MGL-N-532A-5W), with a beam diameter of 1 mm. The control signal for the transistor was caused by another 532 nm continuous wave laser (MGL-N-532A-5W), with a beam diameter of 1.5 mm. The output power of the transistor was detected by a thermal power sensor (LP100, Cnilaser).

The characteristic parameters are calculated by the output performance of the transistor. The power of the input and control illuminations are defined as *P*_*in*_ and *P*_*control*_. When the transistor is on, the output power is at a high value. The stable output power detected is defined as $P_{out\_on\;}$. When the control laser is turned on and the transistor is off, the output power decreases. For the circumstance that the output power achieves stability, the mean value of the stable output power is used to represent the cutoff performance of the transistor and defined as $P_{out\_off}$. For the circumstance that the output power does not reach a balance within the experiment time span, the minimum value of the output power is used as $P_{out\_off}$. With the defined values, related parameters can be calculated with the following equations.

In order to analyze the transfer efficiency of the transistor at on state, the forward transmittance is defined as:

(Equation 1) $$\begin{array}{c} T_{forward}=\frac{P_{out\_on}}{P_{input}}\times 100\%\text{.} \end{array}$$

The isolation capacity of the transistor is defined with the comparison value of the output power between the on and off state.

(Equation 2) $$\begin{array}{c} I=10\times \mathrm{lg}\left(\frac{P_{out\_on}}{P_{out\_off}}\right)\text{.} \end{array}$$

The amplification ratio is defined as the enlargement times between the output power when the transistor is on and control power.

(Equation 3) $$\begin{array}{c} A=\frac{P_{out\_on}}{P_{control}} \end{array}\text{.}$$

### Characterization of optical logic gates

The photothermal actuator used for optical logic gates was made from PNIPAAm hydrogel prepared by the ice templet method and the LCE. The input irradiation signals were produced by two 532 nm continuous wave laser (MGL-N-532A-5W) with some spectroscopes and reflectors.

### Simulation

The effects of the designed all-optical devices are based on the deformation of the actuating cladding. Theoretical analysis and simulation of photothermal response of cladding help to understand the deformation of the structure and is the foundation for designing and verifying the devices with different target functions. The light triggered phototropic behavior of the responsive waveguide is a transient multi-physics process. For hydrogels, the process involves heat transfer and mass diffusion, together with the large deformation. The commercial multi-physics modeling software COMSOL is used to model and calculate the complex behavior. Details of the simulations methods, models and results are provided in Text S2.

### Data analysis

All the photos and videos were recorded by a camera (Olympus EM10). Video sequences were clipped with video editing software (Adobe Premiere Pro). The motion data were extracted from the movie by using the physical software (Tracker) for video analysis.

## Results

### Design principle and operation mechanism

**Figure 2:**
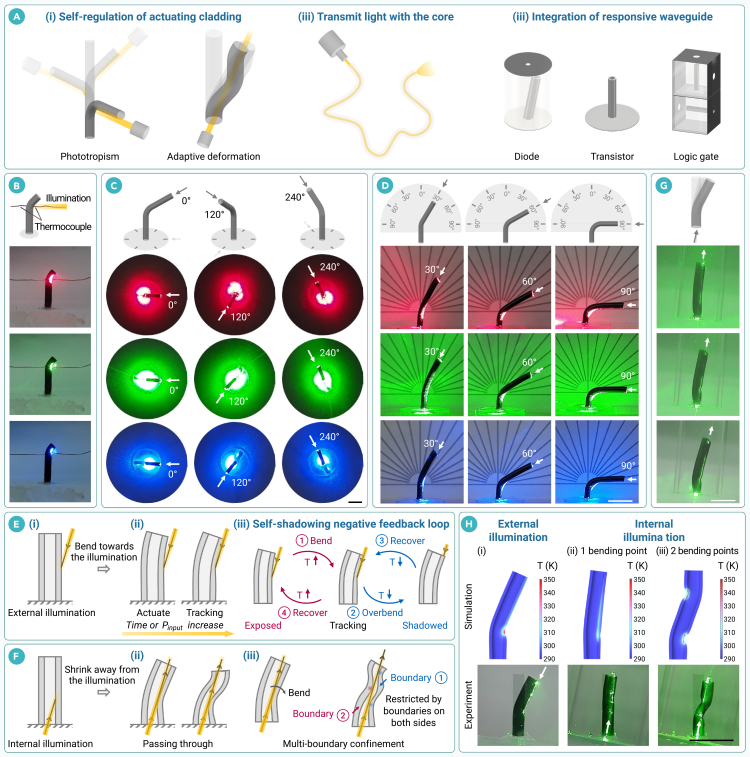
Behavior and mechanism of the photothermal-responsive waveguide (A) Design principles of all-optical devices based on responsive waveguides. (B) PNIPAAm hydrogel actuating cladding bent toward the incident direction of illumination on the outside of the actuator, with two thermocouples attached to the front and back sides of the illumination area. The wavelengths of the red, green, and blue lasers were 671, 532, and 457 nm. (C) Azimuth angle test for the LCE cladding actuator. (D) Zenith angle test for the LCE cladding actuator. (E) Phototropic bending process of the actuating cladding when light illuminates the outer surface and the self-shadowing negative feedback mechanism. (F) Behavior of the actuating cladding with illumination on the inside with the multi-boundary confinement. (G) Deformation of the actuating cladding under internal illumination from different incident angles. The illumination power was 197 mW. (H) Different deformation behaviors of the actuator under external illumination and internal illumination illustrated with simulations and experiments. The cladding was prepared with a PNIPAAm hydrogel, and the core was prepared with a PAM hydrogel. The powers for (i), (ii), and (iii) were 202, 265, and 352 mW. Scale bars, 1 cm.

For the basic unit of the self-regulated all-optical device, an optical fiber-inspired structure consisting of an actuating cladding (Figure 2A (ⅰ)) and a light-guiding core (Figure 2A (ⅱ)) is adopted (Figure S1). Through targeted integration strategies, this architecture enables implementation of essential optical components such as diodes, transistors, and logic gates (Figure 2A (ⅲ)). The actuating cladding is constructed from photothermal-responsive materials, including PNIPAAm hydrogels and LCEs. Their response relies on a sequence of coupled processes: light absorption, photothermal conversion, and thermally induced deformation. Absorption spectrum analysis (Figure S2) reveals that incorporation of rGOs into PNIPAAm hydrogels and Indigo into LCEs significantly broadens the light absorption range, enabling a broadband photothermal response. The thermal properties of the materials were characterized using differential scanning calorimetry (Figure S3), which reveals a lower critical solution temperature (LCST) of approximately 33°C for the PNIPAAm hydrogels and a nematic-to-isotropic transition temperature of 54°C for the LCEs. Based on swelling kinetics experiments (Figure S4), the normalized volume of PNIPAAm hydrogel decreased from 1 to 0.6 within 20 s at environmental temperatures of 45°C, demonstrating its thermoresponsive behavior.

To characterize the photothermal conversion process, temperature changes under red, green, and blue illumination were recorded using thermocouples placed on both the illuminated and shaded sides of the PNIPAAm-based responsive cladding (Figure 2B). The illuminated surface exhibited a rapid temperature increase above 42°C (above LCST) within 5 s with the input power of 320 mW, whereas the back surface temperature remained below the threshold (Figure S5). This localized temperature gradient triggered contraction in the light-exposed region, whereas the unexposed region retained its volume, resulting in directional bending toward the light source. Owing to the axisymmetric design, the cladding demonstrates omnidirectional phototropic bending in response to incident light from any spatial direction. The direction of incidence is defined by the azimuth and zenith angles (Figure S6). For the PNIPAAm cladding, tropistic bending was observed over azimuth angles from 0° to 360° and zenith angles from 0° to 32° (Figure S7). Additionally, comparable bending behavior was observed for the LCE cladding under red, green, and blue laser illumination with azimuth angles ranging from 0° to 360° (Figures 2C and S8A; Video S1) and zenith angles ranging from 0° to 90° (Figures 2D and S8B; Video S2). Under 200 mW illumination, the LCE actuating cladding aligned stably with the incident direction in all cases, reaching steady state within 40 s. The tropistic bending is also applicable for UV and NIR light, with wavelengths of 365 and 808 nm (Figure S9) and the light with different polarization states (Figure S10).

Video S1. Tracking light from various azimuth angles under red, green, and blue lasers The sample which was made from RM257 LCE was set in the middle of an electric rotating platform. The inner diameter of the actuator is 1 mm and the outer diameter of the actuator is 2.3 mm. Originally, the actuator was perpendicular to the rotating plane. Then, the illumination shone on the outer side of the actuator. The state of the rotating platform remained stable for 30 s. In response, the actuator bent toward the incident direction and aligned with the illumination ultimately. After that, the platform turned 60° and remained at that state for 30 s while the actuator adaptively rotated to follow the illumination from another direction and reached a new stable. The process that the platform rotated 60° and the actuator reacted continued until the platform had turned 360°. The wavelength for the laser of the left case is 671 nm. The wavelength for the laser of the middle case is 532 nm. The wavelengths for the laser of the right case were 457 nm. The light powers for all the cases were 200 mW.

Video S2. Tracking light from various zenith angles under red, green, and blue lasers The sample was the same as Video S1. The illumination shone from different zenith angles of 0°, 30°, 60°, and 90°. As the laser turned on, the actuator bent toward the illumination direction and aligned with the incident light ultimately. The wavelengths of the laser were 671, 532, and 457 nm in order with the video and the light power was 200 mW.

The above results suggest a wide range of bending angles that the soft optomechanical structure can achieve. However, actuation alone cannot guarantee successful alignment and instant tracking of incident light. The actuation needs to be automatically terminated once the whole device is pointing at the light source and automatically restarted once misaligned (Figure 2E (ⅰ)–(ⅱ)). This self-regulated behavior is modulated by a self-shadowing negative feedback loop,^32^ as illustrated in Figure 2E (ⅲ). If an external photonic stimulation illuminates the external surface of the device, the localized heating causes tropistic bending toward the light. As bending progresses, the distal end aligns with the light path and begins to cast a shadow on the previously illuminated region, reducing its temperature to below the critical threshold, and the shrinking part tends to recover. This feedback cycle continues until a steady state is reached, with the structure stably pointing toward the incident light, which can then be coupled into and transmitted in the transparent core (Figure S11). This behavior autonomously emerges through the interplay of light, heat, and mechanical deformation, without additional intervention.

If an external illumination is not shone on the surface of the cladding but is directly coupled into the waveguide, phototropic photonic transmission is also autonomously achievable (Figure 2F (ⅰ)). When the light beam is reflected at the core-cladding interface, the internal illuminated region shrinks, generating cross-sectional asymmetry of the deformation; hence, the cladding bends, allowing the incident light to pass through the device (Figure 2F (ⅱ)). Unlike external illumination, this internal bending process may involve multiple spots of deformation (three bending points, for example, as shown in Figure 2F (ⅲ)). The concurrent deformations eventually generate a path for light guidance. We find that the process of deformation is uncertain and sensitive to different directions (Figure 2G) of incident light shining into the waveguide, but the ultimate light-guiding effect can always be expected, which reveals the self-adaptive characteristic of the responsive waveguide.

We established a finite element model (FEM) and an experimental setup to analyze the self-regulating behavior. The FEM accurately agreed with the experimental results (Figure S12) and matched the deformation behavior under external (Figure 2H (ⅰ)) and internal (Figure 2H (ⅱ)–(ⅲ); Video S3) illumination. When the power of the illumination slightly changes from 265 mW (Figure 2H (ⅱ)) to 352 mW (Figure 2H (ⅱi)), the structure adjusts its shape from one bending point to two bending points to form the light pathway.

Video S3. Illuminating the inner side of the actuating cladding The actuator was built with the PNIPAAm hydrogel (prepared at room temperature and allyl disulfide added) actuating cladding and the PAM inner layer. The outer diameter of the actuator is 2.4 mm and the inner diameter of the actuator is 1.5 mm. The actuator was stuck obliquely to a glass substrate, which was set on a glass tube. The light shone from the top of the actuator vertically through the inner layer of the actuator and illuminated the inner layer of the actuating cladding. When the light power was 265 mW, the actuator bent toward the direction of the incident light and the light passed through the inner layer of the deformed actuator. When the light power came to 352 mW, the actuator bent sharply and ultimately reached the stable state that the light passed through the actuator with two interaction bending points. The results reveal the adaptability of the actuator under the inner illumination to form the path.

The mechanism of the responsive actuator is applicable to different types of photothermal-responsive materials. The characteristics of the response depend on the geometric parameters and material (Figure S13). For PNIPAAm hydrogels, due to stiffness and microstructure (Figure S14) difference, actuators with smaller diameters and prepared with the ice template methods^39^^,^^47^^,^^48^ have faster response speeds and LCE actuators can be actuated in air (Text S1). Furthermore, the actuation mechanism exhibits reversible deformation characteristics. During the responsive process, the materials maintained elasticity without undergoing plastic deformation (Figure S15). Through 100 cyclic illumination tests (Figure S16), we demonstrated that the hydrogel actuator preserves its response kinetics and recovery properties with no performance degradation. As the actuators are photothermal responsive, the environmental parameters affect the response characteristics (Text S3; Figure S17). For PNIPAAm hydrogels, the response speed increased with the relatively higher temperature, but, when the temperature exceeded the LCST, the deformation ability was restricted (Figure S17A). Notably, the core of the waveguide can be hollow (filled with water) or contain a transparent inner layer to guide light. For example, Figure S18A shows the core made of a transparent PAM hydrogel guiding the oblique incident light and Figure S18B shows a knotted PAM hydrogel core guiding the laser. As a linear optical system, this self-regulated, multimode light-guiding system transfers and regulates rich information, including a wide range of spectrum and complex optical patterns (Figure S18C). On the basis of the key functions of self-regulated light sensing, tracking and guiding, we designed and fabricated a series of all-optical devices as follows.

### Diode-like soft system

**Figure 3:**
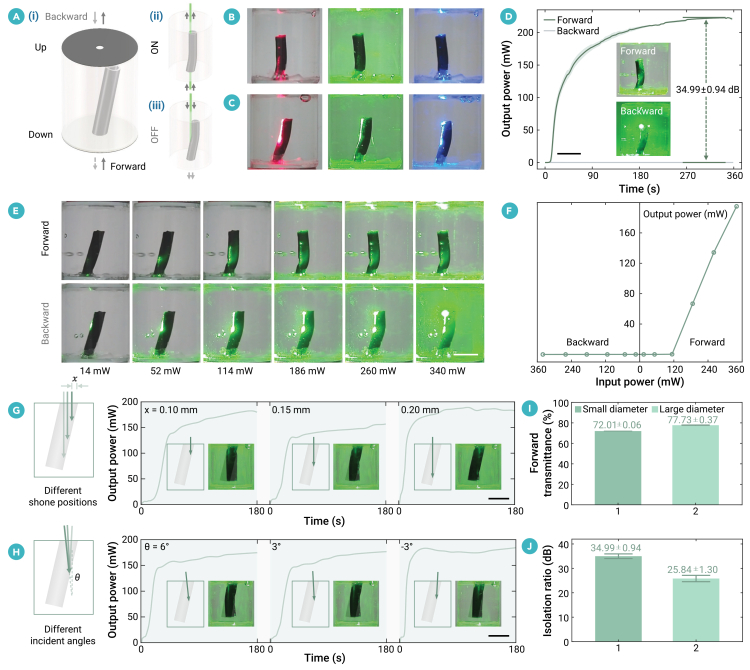
Principle and performance of the all-optical diode (A) Structure and mechanism of the diode. (B and C) Photos of the diode under forward (B) and backward (C) illumination, with the experimental results under red, green, and blue lasers. (D) Time evolution of the output power under forward and backward illumination, with an isolation ratio of 34.99 ± 0.94 dB under an input power of 310 mW. (E) Photos of the diode under different powers of illumination from the forward and backward directions. (F) Output power versus input power plot revealing the characteristics of the diode. (G) Schematic diagram and time evolution of the output power under illumination with different illumination points. Values of x were 0.10, 0.15, and 0.20 mm, from left to right. (H) Schematic diagram and time evolution of the output power under illumination with different incident directions. Values of θ were 6°, 3°, and −3°, from left to right. For (G) and (H), the input power was 252 mW. The diode was made with a PNIPAAm hydrogel. (I and J) Characteristics of diodes with different diameters, including the forward transmittance (I) and isolation ratio (J). Scale bars, 1 cm. Error bands indicate SD, *n* = 3.

An all-optical diode is a two-port system that transmits light traveling forwards and blocks other light. In contrast to the conventional nonlinear optical approach, a self-regulated optomechanical device can react to light from different directions and allows light guidance in only one direction. The responsive waveguide is fixed on the bottom side of a cylindrical glass tube and tilted (Figures 3A (ⅰ) and S19). Two light-absorbing films cover the top and bottom of the tube. One transparent opening is designed on each film to serve as the port to allow light transmission. When light enters the structure from the port on the bottom port, the light beam hits the inner side of the actuating cladding, and the tilted device turns upright guiding the light through the top port of the device (Figure 3A (ⅱ)). Conversely, when light enters from the top port, the light beam is randomly scattered and cannot pass through the bottom port (Figure 3A (ⅲ)). We tested the design by utilizing the PNIPAAm hydrogel as the active material for the actuating cladding, and the all-optical diode works for incident light with wavelengths of 671, 532, and 457 nm (Figures 3B and 3C; Table S1). Comparing the images of the off state (Figure 3C) with those of the on state (Figure 3B), the light scattering in the case of backward illumination is much stronger, and ultralow light transmission is recorded at the bottom port. Under an input power of 310 mW, the wavelength-independent diode achieved an isolation ratio of 34.99 ± 0.94 dB (Figure 3D; Video S4), which is comparable with that of the superior all-optical diode that works for only a limited spectrum.^7^^,^^19^^,^^22^^,^^49^

Video S4. Responsive behavior of diode The diode was made from a PNIPAAm hydrogel waveguide with the inner diameter of 1.2 mm and the outer diameter of 2.5 mm. The actuator was obliquely stuck to a glass substrate and sealed in a glass tube, which was filled with water. The diode was set on the light power meter and the incident light shone from the top. The power of the incident light was 310 mW. When the light entered the diode from the forward direction, the inner side of the actuator was illuminated and the actuator deformed along the light direction. Ultimately, the light passed through the waveguide to reach the output port and the diode was on. When the light entered the diode from the opposite direction, the outside of the actuator was illuminated and the actuator bent toward the light direction. Although the actuator pointed the incident direction, most of the light power was blocked by the actuating cladding, which could be observed through the bright scattered spot on the top of the actuator. As a result, the output of the diode was weak, and the diode was off.

To further investigate the characteristics of the diode, the responses under different input illumination powers were evaluated. Under different illumination powers (input), Figure 3E shows the final deformation state of the diode, and Figure S20A shows the time evolution of the light power transmitted through the device (output). The example device exhibited an activation input power of 186 mW. With stronger input power, the response time to achieve the on state is shorter. The optical transmission behavior of the diode is shown in Figure 3F. With different input powers, the output power of the backward illumination remained close to 0. Beyond the activation power, the deformation of the waveguide allows the light to sufficiently pass through (Figure S20B), and the isolation ratio is maintained above 33 dB (Figure S20C). For the device under investigation, the maximum bending speed of the actuator is calculated to reach 2.38°/s when the input power is 340 mW (Figure S20D). The speed can be faster if we miniaturize the device. The maximum response speed can reach 95.08°/s for the PNIPAAm hydrogel and 648.93°/s for the LCE, and the LCE column can oscillate at a frequency of 12 Hz (Figure S21; Video S5). The activation input power can thus be manipulated if needed. The theoretical response frequency of the device can be further extended to the kHz range.^50^^,^^51^ The requirement for sufficient temperature gradient generation limits further decrease of the geometric parameters (Text S2.3; Figure S22), but alternative approaches such as utilizing materials with distinct thermoresponsive properties can help to overcome heat transfer limitations and are promising for micron-level fabrication.^35^^,^^52^

Video S5. Fast response of PNIPAAm hydrogel and LCE columns Fast response of PNIPAAm hydrogel and LCE columns revealed with the bending and the oscillating process while the columns were illuminated. Demo 1: fast response of PNIPAAm hydrogel column. The sample was made from PNIPAAm hydrogel prepared with ice template method. The diameter of the sample is 0.66 mm. The illumination shone from the horizontal direction and the power was 485 mW. The actuator bent toward the incident direction and the maximum angular velocity of the bending process is 95.08°/s. Demo 2: fast response of LCE column. The sample was made from the LCE prepared with RM257 and RM82, which were equal of mass. The diameter of the sample is 0.36 mm. The illumination shone from the horizontal direction and the power was 123 mW. The column bent toward the incident direction rapidly and the maximum angular velocity of the bending process is 648.93°/s. Demo 3: oscillation of LCE column. The sample was made with the same method as demo 2. The diameter of the sample is 0.37 mm. The illumination shone from the horizontal direction and power was 84 mW. The column bent toward the incident direction and then oscillated around the incident direction. The frequency of the oscillation is 12 Hz.

The diode is self-regulating and stable in the case of small external disturbances. When the input condition is slightly varied in terms of position and incident direction, the diode can reach a new steady state by self-adjusting the deformation. For example, in the case of minor sliding of the position with the gap between the light and the edge of the actuator varying between 0.1, 0.15, and 0.20 mm, the light illuminated different spots on the inner wall of the actuating cladding. The original deformation spot recovered, and the new illumination spot deformed to allow light transmission (Figure 3G). When the direction of incidence is changed from 6°, 3°, to −3°, the diode reconfigured to a different angle to adaptively align with the incident light and allowed forward transmission (Figure 3H).

The self-regulated all-optical diode exhibits a high overall energy efficiency. The highest forward transmittance is 77.73% ± 0.37% (PNIPAAm), indicating that only 22% of the input power is absorbed for actuation or scattered as loss. The scaling effect is shown in Figures 3I and 3J. Diodes with smaller inner (1.2 mm) and outer (2.5 mm) diameters have larger isolation ratios (∼35 dB), and diodes with larger inner (2.7 mm) and outer (3.8 mm) diameters (Figure S23) have higher forward transmittance. The design principle is also suitable for other photothermal-responsive materials, such as PNIPAAm hydrogels prepared with the ice template method (Figure S24) and LCEs (Figure S25). Compared with reported counterparts in the literature,^7^^,^^8^^,^^19^^,^^22^^,^^49^^,^^53^^,^^54^ our self-regulated all-optical diode system exhibits sharply different operating bandwidths and energy efficiencies (Figure S26; Table S2). For example, the nonlinear effects-based all-optical diode exhibits energy efficiency below 1%,^7^ whereas the self-regulated all-optical diode presents an overall efficiency of 77% and a theoretical efficiency of over 92.7% (Figure S11B) together with a wide bandwidth by incorporating extensively studied photothermal nanoparticles with wide absorption spectra.^44^

The design principle of the diode based on the responsive waveguide is not restricted to that shown in Figure 3, in which the transmission of a laser beam that can directly couple into the waveguide and interact with the internal surface of the actuating cladding is regulated. A similar diode design method (Figure S27) can be used to regulate a reverse conduction behavior with different input modes (more in Text S4; Figure S28).

### Transistor-like soft system

**Figure 4:**
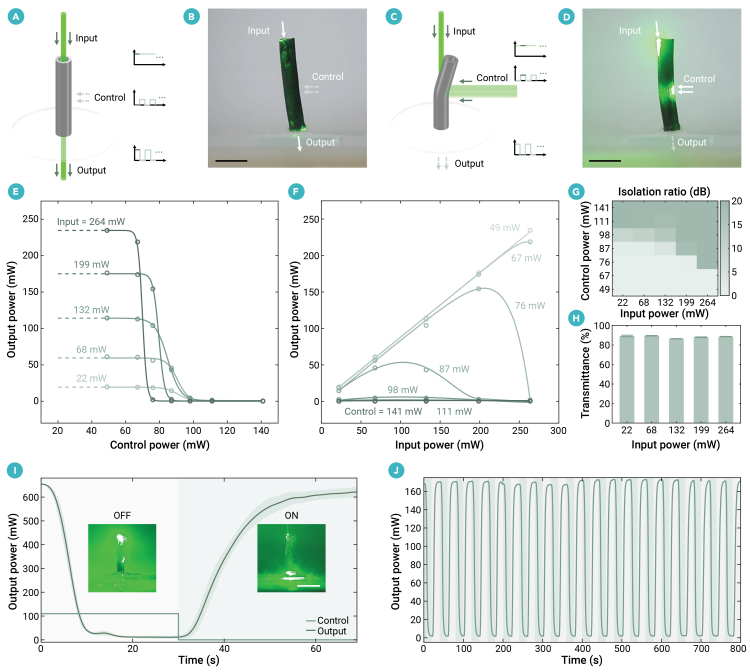
Principle and performance of the optical transistor (A and B) Principle (A) and photo (B) of the transistor in the conducting state. (C and D) Principle (C) and photo (D) of the transistor in the cutoff state. For (B) and (D), input power, 223 mW; control power, 150 mW. (E) Transfer characteristics of the transistor. (F) Output characteristics of the transistor. (G) Isolation ratio of the transistor. (H) Transmittance of the transistor in the conducting state. (I) Time evolution of the amplify behavior of the double-layer transistor (input power, 755 mW; control power, 110 mW, error bands indicate SD, *n* = 3). (J) Cyclic actuation test of the transistor, with input power of 185.08 mW and control power of 116.45 mW, which was turned on for 20 s and turned off for 20 s for 20 cycles. The transistors of (B), (D), and (I) were built with PNIPAAm prepared with the ice template method with a PAM hydrogel core. The transistors of (E) to (H) and (J) were built with PNIPAAm prepared with the ice template method without a PAM hydrogel core. The calculation methods for the parameters mentioned in (G) and (H) are explained in the materials and methods. Scale bars, 1 cm.

The all-optical transistor is a three-port device designed to control the on/off state of the propagation of one light signal using another one, which is preferably weaker in power than the prior signal.^46^^,^^55^ Analogous to its electronic counterpart, the input, output, and control signals correspond to the source, drain, and gate terminals, respectively (Figure S29). To achieve this function, a photothermal-responsive waveguide is fixed on a base (Figure S30). A light beam propagates through the core, and a control light beam points at the external surface of the actuating layer. In the default conducting state, with the control beam off, the waveguide remains upright, allowing the input signal to propagate unobstructedly (Figures 4A and 4B). Upon activation of the control beam, localized heating induces bending of the waveguide toward the illuminated side, misaligning it with the input path and effectively blocking transmission (Figures 4C and 4D). This transition into a nonconducting “cutoff mode” echoes the behavior of a depletion-mode metal-oxide-semiconductor field-effect transistor (MOSFET) or a junction field-effect transistor (Figure S31; Video S6). In addition to the cutoff mode, we also designed and demonstrated a complementary “transmission mode” (Figure S32), in which the waveguide is initially tilted and thus misaligned with the input beam. Illumination of the sidewall by the control beam then induces photothermal bending that realigns the waveguide with the optical path, enabling light transmission. This mode is conceptually analogous to an enhancement-mode MOSFET or a bipolar junction transistor, further enriching the functionality of our all-optical transistor platform.

Video S6. Responsive behavior of transistor The transistor was made with a double-layer actuator. The inner layer of the actuator was PAM hydrogel and the outer layer of the actuator was PNIPAAm hydrogel prepared with the ice template method. The inner diameter of the actuator is 2.5 mm and the outer diameter of the actuator is 3.3 mm. The input light is set parallel to the axis of the actuator and through the inner layer of the transistor when the actuator is undeformed. The control light is set perpendicular to the actuator. For this case, the input power was 233 mW and the control power was 150 mW. The input light was normally open. When the control light was turned on, the actuator bent toward the direction of the gate light and the input light from the top was shadowed. As a result, little illumination passed through the output port and the transistor was off. When the control light was turned off, the deformed actuator recovered. As a result, the input light could pass through the device to reach the output port and the transistor was on.

For the cutoff mode of the all-optical transistor, we measured its characteristics under various input (22–343 mW) and control (49–141 mW) powers, with the transistor consisting of a PNIPAAm hydrogel-responsive cladding (Figure S33). At a given input power, increasing control intensity led to a progressive decrease in the output signal. Notably, for higher input powers, the control threshold required to induce output suppression decreased, and the rate of output attenuation became more pronounced (Figure 4E). Conversely, at fixed control powers below 49 mW, the output was linearly with the input while, as the control power increased, the output declined with increasing input power, indicating the growing dominance of the gating effect (Figure 4F). A critical switching threshold of the control power was 111 mW, beyond which the output signal remained near zero regardless of the input power, indicating complete shutdown of light transmission (Figure 4F). Under these conditions, the device achieved an isolation ratio exceeding 18 dB (Figure 4G) and a transmission ratio over 86% (Figure 4H). In the conducting regime, the amplification ratio (defined as the output-to-control power ratio) ranged from 0.14 to 2.73, offering broad tunability of the transmitted light intensity (Figure S34A). A double-layer configuration incorporating a PAM hydrogel core demonstrated comparable switching characteristics, further validating the adaptability of the platform (Figure S35).

This device enables lower-power optical signals to modulate the transmission of significantly higher-power beams. Amplification ratio of 5.96 ± 0.02 was achieved with an input power of 755 mW and a control power of 110 mW for a double-layer transistor (Figure 4I). Due to the interaction between the input light and the top region of the actuating layer, the input power cannot exceed the maximum threshold that enables the actuating structure to maintain the recovery capacity (Figure S36; Video S7). In the single-layer design, a lower maximum amplification ratio of 2.73 was achieved (Figure S34) because the double-layer design benefits from the recovery-enhancing effect endowed by the PAM hydrogel core. Importantly, the switching behavior remained robust across multiple operational cycles under fixed input and control powers (Figure 4J), demonstrating high durability and repeatability.

Video S7. Amplification behavior of transistor The transistor was the same as Video S6. The control power was set consistently as 150 mW. When the control light was turned on, the actuator bent toward the illumination direction and the input light was blocked. As the control light was turned off, the actuator tended to recover to the original state. When the input power was 924 mW, the actuator was able to fully recover. When the input power was 967 mW, the actuator could not recover to the original state. The transistor fails when the input power is too strong that the actuator lost its continuous ability to react and recover.

Theoretically, by tuning the numerical aperture of the self-regulated all-optical material system, a deviation as small as 1° in the direction of the light beam can lead to termination of light transmission, yielding an amplification factor of 57.3, which further increases when accounting for positional absorption (Text S2.4). In addition, by properly initially shielding the source light to prevent its direct transmission and regulating it through a small gate input, the transistor can achieve an extremely high amplification factor of 1,000 (Text S2.4). The combination of photoresponsive and light-insensitive materials can further increase the amplification ratio by reducing excessive top-cladding deformation (Figure S37).

### All-optical logic gates

**Figure 5:**
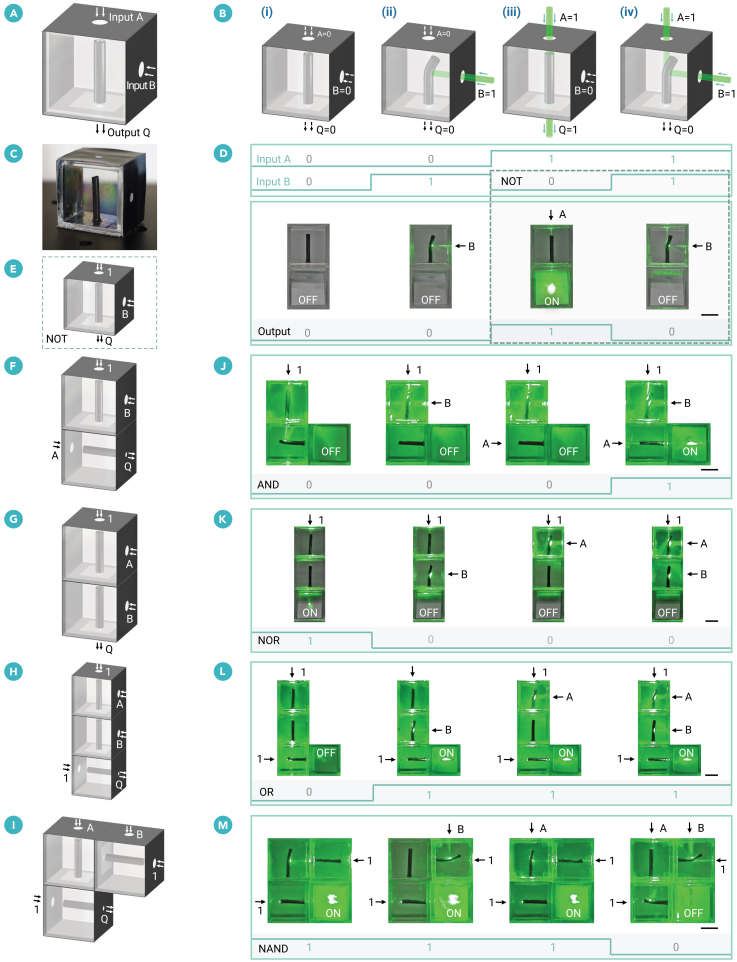
Mechanism and design principle of logic gates (A) Structure of the basic element, and definitions of input port A, input port B, and output port Q. (B) Diagrams of the four kinds of response results for different combinations of inputs. (C) Photo of the basic element packaged with an acrylic box and shading films. (D) Photos of the single-element response results corresponding to (B). (E) Design principle of the NOT gate, with port A defined as a constant input and the output equaling the NOT operation of the signal from port B. The two photos on the right of (D) confirm this principle. (F–I) Design principles of the AND gate, NOR gate, OR gate, and NAND gate. (J–M) Experimental verification of the AND gate, NOR gate, OR gate, and NAND gate with the LCE actuators. Scale bars, 1 cm.

Leveraging the transistor as building blocks, we proposed modular and reconfigurable all-optical logic gates to conduct essential Boolean operations, including NOT, AND, OR, NAND, NOR, etc. (Figure S38). In these configurations, the constant input from the top is defined as input port A, and the control input from the side is defined as input port B. Under this configuration, the transistor operates as a binary logic gate with specific output responses for each of the four input combinations (Figures 5A and 5B). When both A and B are off (Figure 5B (i)), no output is observed. When B is on, the responsive waveguide bends in the illumination direction regardless of the state of A (Figure 5B (ii) and (iv)), blocking the signal and turning the device off. Only when A is on and B is off (Figure 5B (iii)) does the signal pass through the device, generating a positive output. For cascading and integration, the device was housed in a square acrylic enclosure with ports located on the central faces for systematic unit alignment (Figures 5C and S39). The output states were visualized using a tilted diffuse reflective screen. Experimental validation using LCE-based prototypes confirmed the logic behavior of the basic unit across all four input conditions (Figure 5D). Furthermore, quantitative analysis was conducted for the LCE-based basic element (more in Text S5; Figure S40).

For the basic unit, when port A remains a constant input, the output corresponds to a NOT operation on port B (Figure 5E). Since the output optical signal maintains identical characteristics to the input signal, the basic unit enables cascading possibility and the output from one unit is sufficient to actuate at least two subsequent units at the same time (Figure S41). Moreover, at the currently investigated scale, the photothermal effect remains localized within the irradiation region without inducing thermal crosstalk between adjacent devices (Figure S42). By stacking a horizontally placed unit and a vertically placed unit together, an AND gate is formed (Figure 5F), whereas two vertically aligned units form a NOR gate (Figure 5G). Further stacking of a NOT gate at the output of these assemblies yields OR (Figure 5H) and NAND (Figure 5I) operations. The design principles are summarized in Figure S43. These logic functions were confirmed using LCE samples (Figures 5D and 5J–5M; Video S8), and a similar behavior was observed for hydrogel-based devices fabricated via the ice template method (Figure S44; Video S9).

Video S8. Logic gates built with RM257 LCE The logic elements were built with RM257 LCEs. They were stacked together as the design principles. All the deformation processes of the basic element, NOT gate, AND gate, NOR gate, OR gate, and NAND gate are shown in order with different input combinations. The output status of the device was displayed by a sloping placed diffuse reflective surface. When the output is on, there is a bright spot on the surface.

Video S9. Logic gates built with PNIPAAm hydrogel The logic elements were built with ice template method prepared PNIPAAm hydrogels. They were stacked together as the design principles. All the deformation processes of the NOT gate, AND gate, and NOR gate are shown in order with different input combinations.

To further streamline the logic construction, we introduced an alternative five-port element design (Figure S45) capable of realizing all fundamental logic operations (NOT, AND, OR, NAND, NOR, XOR, and XNOR) with only one or two components. Simulations across all 16 binary input states validated the expected responses (Figure S46). The inherent flexibility of this modular system allows the layout and input configuration of logic gates to be readily adapted to diverse application scenarios.

## Discussion

In summary, we present the integration of an optical waveguide, a direction recognition capability, and a self-regulated switch by utilizing a photothermal-responsive actuating cladding and a transparent core. This architecture enables highly adaptive deformations to guide photon transmissions from various directions, governed by an internal feedback mechanism. The phototropic light-guiding and smart switching behaviors enable all-optical diodes, transistors, and Boolean operations without relying on nonlinear optics or resonance. Similar to the many tropistic behaviors in nature, these devices respond to and transmit energy with ultrawide spectral and polarization mode ranges, containing complex information much more than 0 and 1, with a high energy efficiency. Furthermore, the all-optical functionality of the responsive waveguide is not limited to light-tracking mode and potential oscillatory mode could be generated to apply for periodic modulation of constant optical signals (Figure S47). The operating mechanisms are not constrained to specific materials and, for the actuating layer, replacing photothermal absorbers with biological materials such as microalgae^39^ can enhance the biocompatibility for the system. Additional nonlinear or linear optical devices can be integrated into a conscious optical “gatekeeper” if additional functionalization is needed and these functional units can be further cascaded and scaled. This concept can provide self-regulating capabilities for signal sensing, transmission, and processing, opening new routes toward autonomous optical processors, soft robotics, and embodied intelligent material systems.

## Resource availability

### Materials availability

This study did not generate new unique materials.

### Data and code availability

Data are available from the corresponding author upon reasonable request.

## Funding and acknowledgments

The authors gratefully acknowledge the support of the following funding: 10.13039/501100001809National Natural Science Foundation of China (T2488302, T2342010, 52076127, 52406022, and 5247030214), NSFC Young Student Basic Research Program (PhD candidate: 24Z033403036), China National Postdoctoral Program for Innovative Talent (BX20240220), Postdoctoral Fellowship Program of CPSF (GZB20240422 and GZC20241007), Natural Science Foundation of Shanghai (20ZR1471700, 22JC1401800 and 25ZR1402212), 10.13039/501100011415State Key Laboratory of Mechanical System and Vibration (MSVZD202211, MSVZD202301 and MSVZD202401), Shanghai Jiao Tong University 2030 Initiative, Shanghai Jiao Tong University SiYuan Scholar Program. X.Q. acknowledges the support from the Student Innovation Center, the Instrumental Analysis Center, and the Physics Experiment Center at Shanghai Jiao Tong University, and the New Cornerstone Science Foundation through the XPLORER PRIZE. X.Q. is a SANS Exploration Scholar. The funders had no role in study design, data collection and analysis, decision to publish, or preparation of the manuscript.

## Author contributions

X.Q. conceived the concept, designed the experiment, and wrote the manuscript. X.W., G.H., F.Z., F.D., R.L., Z.M., Y. Lin, C.W., Y.Z., B.S., C.H., L.Y., S.Z., Q.L., D.H., H.Q., J.Z., X.C., T.Y., and W.L. carried out the material synthesis, characterization, and systematic demonstration. X.W. and G.H. conducted the optical device experiment and analysis and carried out the numerical simulation. D.Z., W.Z., X.Z., Y. Lu, G.H., and G.M. supervised the theoretical and device modeling. X.Q. and G.M. supervised the project. All authors contributed to the manuscript and approved the final version.

## Declaration of interests

X.Q., X.W., and G.H. are inventors on a provisional patent application related to the described work. The other authors declare no competing interests.

## Lead contact website

https://me.sjtu.edu.cn/teacher_directory1/qianxiaoshi.html.

## Supplemental information

Document S1. Figures S1–S47 and Tables S1–S7, and materials and methods

Document S2. Article plus supplemental information
