# Supplementary material for: Self-regulated optomechanics achieves ultrabroadband and high-efficiency all-optical devices
Source: Innovation (Camb). 2026 Mar 12;7(8):101348. doi: 10.1016/j.xinn.2026.101348 (PMC13457184; doi:10.1016/j.xinn.2026.101348)
Supplement: Supplementary file 1 [file mmc1.pdf]

The Innovation, Volume 7

Supplemental Information

Self-regulated optomechanics achieves ultrabroadband and high-efficiency all-optical devices

Xuning Wang, Guodong Hou, Feiyu Zhang, Feihong Du, Ruhong Luo, Zhenhua Ma, Yezhan Lin, Chunyu Wang, Yifan Zhao, Binzhou Sun, Cenling Huang, Lu Yu, Shanyu Zheng, Qiang Li, Donglin Han, Haixin Qiu, Jiang Zou, Xin Chen, Tiannan Yang, Wei Li, Weihong Zhu, Xiangyang Zhu, Di Zhang, Yanqing Lu, Guang Meng, and Xiaoshi Qian

Materials and Methods

Materials

N-isopropylacrylamide (NIPAAm), 2-hydroxy-1-(4-(2-hydroxyethoxy)phenyl)-2-methylbenzene (Irgacure 2959), 3-(trimethoxysilyl)propylmethacrylate (TMSPMA), acetic acid glacial, 1,4-bis[4-(3-acryloyloxypropoxy)benzoyloxy]-2-methylbenzene (RM257) and dipropylamine (DPA) were purchased from Adamas-beta. Acrylamide (AAM) and indigotin were purchased from Aladdin. N,N'-methylenebisacrylamide (BIS), 2-hydroxy-2-methylpropiophenone (Darocur 1173) and allyl disulfide were purchased from Sigma-aldrich. Dimethyl sulfoxide (DMSO) and dichloromethane were purchased from General-reagent. Hydrazine monohydrate was purchased from Alfa Aesar. Pentaerythritol tetra(3-mercaptopropionate) (PETMP) and 2,2'-(ethylenedioxy)diethanethiol (EDDET) were purchased from Meryer. Ethanol and haxane was purchased from Greagent. Graphene oxide (GO) was purchased from Jiangsu XFNANO Materials Tech Co., Ltd.

Sample preparation

Silica gel molds

The structures of the molds were shaped into stepped shafts by machining method with stainless steel. Silica gel A and B were mixed at ration of 1:1 by weight. The air bubbles inside the mixture were removed in a vacuum environment. The silica gel mixture was added to the container with the stepped shaft mold fixed inside the mixture and then the mixture cured at 80°C. The mold was removed and the silica gel mold was obtained.

Silane-treatment of substrate

The substrates were silane-treated in order to immobilize the hydrogel sample on them. Glass slides were ultrasonicated with acetone and ethanol respectively for 6 minutes. After the glass slides were dried with nitrogen, they were treated with oxygen plasma for 300 seconds. Then, they were immersed in a mixture of 0.5 ml 3-(trimethoxysilyl)-propyl methacrylate (TMSPMA), 0.6 ml of 99% acetic acide and 100 ml of ethanol for 8 hours and washed in ethanol then dried.

Fabrication of PNIPAAm cladding

The precursor solution for PNIPAAm hydrogels prepared at room temperature contained 30 wt% NIPAAm monomer, 1.5 wt% crosslinker BIS, 1vol% photo-initiator Darocur 1173 in DMSO with 0.3 wt% GO. In some circumstances, the solution was also added with 0.1 vol% Allyl Disulfide. A metal column matching the inside diameter of the mold was put inside the silica gel mold and the mixture solution was injected into the mold to fill the free space. The mold filled with mixture solution was treated with UV curing for 60 seconds. (The illumination time for the solution with Allyl Disulfide was 250 seconds.) The cured gel structure was pulled out from the mold and immersed into the DI water to remove unreacted monomers and DMSO. Then the hydrogels were put into 0.33 M hydrazine aqueous solution to reduce GO to rGO.

Fabrication of PNIPAAm cladding with ice templet method

The precursor solution for PNIPAAm hydrogel prepared by ice templet method was prepared with 20 wt% NIPAAm monomer, 1.5 wt% crosslinker BIS, 3.36 wt% photo-initiator Irgacure in DI water with 0.3 wt% GO. The molds filled with the solution were placed into a refrigerator at -18°C overnight to ensure the solution frozen completely. Then the samples were cured with a UV

lamp at -18°C. The cured hydrogels were removed from the molds and immersed into DI water for full water exchange. Then the hydrogels were put into 0.33 M hydrazine aqueous solution to reduce GO to rGO.

Fabrication of PAM hydrogel and double layer cylinder actuator

The precursor solution for PAM hydrogel was prepared with 25 wt% AM monomer, 1.54 wt% crosslinker BIS, 2.24 wt% photo-initiator Irgacure in DI water. The silica mold prepared with the metal bar fitted the inner layer diameter of the double layer actuator was used for the fabrication of PAM hydrogel inner layer. The mixture solution was injected into the mold and a silane-treated glass slide covered the top of the mold, followed by UV curing for 200 seconds. Then the sample stuck to the glass slide was pulled out carefully and immersed into DI water to remove unreacted monomers and for full water exchange. With the prepared PNIPAAm tubular sample and the PAM sample, the double layer actuator was assembled. The PAM cylinder passed through the PNIPAAm tube, and the PNIPAAm tube was stuck to the glass slide with UV adhesive, to establish the double layer structure.

Fabrication of LCE cladding

The precursor solution for LCE was prepared with 0.4 g RM257, 20 mg Irgacure 2959, 4 mg Indigo, 20 µl PETMP, 86 µl EDDET and 8 µl DPA dissolved in 1200 µl dichloromethane. Then the mixture was ultrasonic dispersed for 10 minutes. After the solution was placed in a vacuum environment to eliminate air bubbles, the mixture solution was added into the mold with a thin glass tube in the middle to form a tubular structure. Then the mold was stood for 24 hours to evaporate the solvent and complete the reaction. After the LCE tube was removed from the mold, the sample was stretched 2 times of initial length, and it was cured under UV light for 4 hours to fix the LCE sample in monodomain state.

<u>Optical devices preparation and arrangement</u>

Fabrication of optical diode

As shown in Figure S19, the photothermal responsive waveguide was stuck to the glass slide with an included angle between the axis of the sample and the direction perpendicular to the glass slide. Then, the sample was sealed in a glass tube. The glass tube was filled with DI water when the sample was made of hydrogel. Shading films with holes matched the inside passage of the tilted sample were stuck to the glass slide on both side of the device, for the sake of reducing the effects of scattered light. When the diode is in the conducting state, the laser beam vertically irradiates the port on the down side which is connected with the sample. When the diode is in the cut-off state, the laser beam comes from the opposite direction.

Fabrication of optical transistor

As shown in Figure S30, the photothermal responsive waveguide was stuck to the glass slide, and the shading film with holes matched the inside passage of the waveguide was stuck to the bottom of the glass slide. The constant laser was adjusted parallel to the axis of the tube and pass through the inside passage. The control laser was horizontally exposed to the outside of the waveguide. Corresponding to the definition of traditional transistors, the constant input, control and output refer to source, gate and drain respectively.

Fabrication of optical logic gates

As shown in Figure S39, the basic unit that would constitute the logic gates was fabricated. The photothermal responsive waveguide was stuck to the glass slide vertically and then fixed in an acrylic box. When the sample was made from hydrogel, the acrylic box was filled with DI water. Four side faces of the box was covered with shading films with holes in the center. (As the shown direction, the shading films on the left, right and top sides are not necessary.) From Figure S39B, the ports on the top and right side are the input ports and the port on the bottom side is the output port. Different logic gates were architected by stacking the basic units together.

Characterization

Optical properties

The absorbance of membrane containing photo-absorbers was characterized by the UV-NIR spectrometer (Lamda 950). The wavelength range detected was set from 250 to 2000 nm. Figure S2A shows the absorbance of the PNIPAAm hydrogel membrane containing rGOs as photo-absorbers. The thickness of the membrane is 1.2 mm and the absorbance is over 1.46 when the wavelength is above 370 nm. The absorption effect for rGOs is relatively consistent and strong. Figure S2B shows the absorbance of the RM257 LCE membrane containing indigo as photo-absorbers. The thickness of the membrane is 0.5 mm. In the UV-Vis band, the absorbance is around 1. In the NIR band, the absorbance is lower and around 0.3. rGO and indigo perform a strong absorption efficiency over a wide wavelength range. Figure 2C shows the transmittance of PAM hydrogel across the 250–2000 nm wavelength range. The PAM hydrogel demonstrated over 90% transmittance in the 250–1000 nm range. Due to the strong infrared absorption by water, the transmittance of PAM hydrogel decreased significantly beyond 1250 nm. Since the design methodology presented in this study is material-independent, replacing the inner layer material can effectively circumvent the issue of water absorption in the infrared band. The refractive index of PNIPAAm hydrogel and PAM hydrogel was tested with the total internal reflection method and the refractive index is 1.31 and 1.36 (wavelength, 587.6 nm), respectively.

Differential scanning calorimetry (DSC)

Corresponding to the differential scanning calorimetry (DSC) measurements (Figure S3) (DSC25, TA), the low critical solution temperature (LCST) is 33℃ for the PNIPAAm hydrogels made at room temperature without allyl disulfide and for the PNIPAAm hydrogels made at room temperature with allyl disulfide, the LCST is 32 ℃. As to the PNIPAAm hydrogels made with the ice templet method, the LCST is 34 ℃. For RM257 LCE, the nematic-to-isotropic transition temperature (T_{NI}) is 54 ℃.

Swelling ration and kinetics

The volume change of the hydrogel in water bath with different temperature was recorded through a manual optical inspector (ZQ-601). The hydrogel cylinder which was fully swollen was put in the container at room temperature, then the hot water (with the temperature of 45 ℃, 60 ℃ and 75 ℃) was poured into the container to cover the hydrogel fully. The hydrogel was heated and the volume of the hydrogel shrunk. The overall process was recorded, and later the statistics of the volume variation was marked and calculated with software Tracker and Matlab.

Mechanical properties

Mechanical properties of PNIPAAm hydrogels prepared at room temperature were tested by a dynamic mechanical analyzer (DMA, TA Instruments, Q850). The sample was fully swollen

before testing. The stress-strain curves of the hydrogels with different mixture and RM257 LCEs are shown in Figure S15A-B. With the experimental results, Young's modulus (calculated from the linear region at 10% strain) and toughness values for both the hydrogels and liquid crystal elastomers were calculated and listed in Table S7. For the PNIPAAm hydrogel, minor differences between loading and unloading paths were observed (Fig. S15C), suggesting the presence of slight hysteresis. Notably, the unloading curve consistently returned to near the origin point, demonstrating that the PNIPAAm hydrogel similarly maintained elastic behavior without measurable plastic deformation under the investigated experimental conditions. For the cyclic tensile characteristics of the LCE, we subjected it to 50 repeated cycles of stretching to 50% strain followed by recovery (Fig. S15D). Owing to the viscoelastic property of LCE, hysteresis was observed in the stress-strain curves during cyclic loading. However, no significant variation was detected between successive cycles after multiple deformation cycles, indicating that the LCE material also exhibits fatigue-resistant properties and no detectable plastic deformation within the tested strain range.

Microscopic morphology

The fully swelled hydrogels were frozen by liquid nitrogen and treated with freeze-dryer (Biocool) for 48 hours. The cross-sectional morphology of PNIPAAm hydrogels were observed by a scanning electron microscope (NOVA NanoSEM 230). The microscopic morphology of PNIPAAm prepared at room temperature is shown in Figure S14A, and the microscopic morphology of PNIPAAm prepared with ice templet method is shown in Figure S14B. The pores of the hydrogel prepared with ice templet method is much larger than that of the hydrogel prepared at room temperature, while the spacing between the pores is wider, which suits the response performance of samples made with different approaches.

The waveguides made from hydrogels and LCEs were cut along the circular section. The morphologies of the cross-sections were photographed with an optical camera (Olympus EM10). Figure S1A corresponds to the double layer hydrogel with PNIPAAm hydrogel as the actuating cladding and the PAM hydrogel as the guiding core. Figure S1B corresponds to the hollow RM257 LCE actuator.

Supplemental Texts

1. **Phototropic bending performance of actuators with different geometric parameters and materials**

Phototropic bending performance of actuators is related with the geometric parameters and materials. Bending and recovery process of different samples with different geometric parameters and made from different materials were analyzed. The illumination direction is horizontal. The images of the bending process and time evolution of angle variation are shown in Figure S13.

The influence of geometric parameters is illustrated with sample 01-03. Figure S13B shows the time evolution of angle variation for sample 01 which was made from room temperature method prepared PNIPAAm hydrogel with 0.48mm diameter under the illumination power of 163 mW, 250 mW and 338 mW. Figure S13C shows the time evolution of angle variation for sample 02 which was made from room temperature method prepared PNIPAAm hydrogel with 1.09 mm diameter under the illumination power of 85 mW, 163 mW, 250 mW and 338 mW. Figure S13D shows the time evolution of angle variation for sample 03 which was made from room temperature method prepared PNIPAAm hydrogel with 2.30 mm diameter under the illumination power of 163 mW, 250 mW and 338 mW. From (B), (C) and (D), when the diameter increases, the response speed increases as well. Under 163 mW illumination, both Samples 01 and 02 achieved stable light-alignment within the test duration, with response times of 27 s and 143 s respectively. To standardize comparisons, we evaluated the time required for 10° bending, as some samples failed to reach full alignment due to limited power or time constraints. The 10° bending times for Samples 01, 02, and 03 under 163 mW were 2.7 s, 4.4 s, and 60.8 s, confirming faster responses with smaller diameters.

The influence of materials is illustrated with sample 02, 04-06. Figure S13E shows the time evolution of angle variation for sample 04 which was made from room temperature method prepared PNIPAAm hydrogel with allyl disulfide added and 1.08 mm diameter under the illumination power of 85 mW, 163 mW and 250 mW. Sample 04 (1.08 mm diameter, containing allyl disulfide) demonstrated enhanced response speed relative to Sample 02 (1.09 mm, without allyl disulfide), requiring 134 s for full alignment and only 2.5 s for 10° bending, suggesting that allyl disulfide incorporation improves actuation speed, while no obvious promotion is found for recovery speed. All samples prepared at room temperature (01-04) exhibited recovery times exceeding 1 minute. Figure S13F shows the time evolution of angle variation for sample 05 which was made from ice template method prepared PNIPAAm hydrogel with 0.66 mm diameter under the illumination power of 27 mW, 85 mW, 163 mW, 250 mW and 338 mW. Sample 05, an ice-template method prepared PNIPAAm hydrogel (0.66 mm diameter), achieved ultrafast actuation despite limited maximum bending angles. It required merely 0.9 s for 10° bending and 10.4 s for full recovery under 163 mW illumintaion, outperforming room-temperature prepared counterparts. Figure S13G shows the time evolution of angle variation for sample 06 which was made from RM257 LCE with 1.03 mm inner diameter and 2.33 mm outer diameter under the illumination power of 68 mW, 119 mW and 167 mW. LCE sample operated in air and showed rapid response (1.7 s for 10° bending, 27 s for alignment with a 45 s recovery time under 167 mW illumination). From Figures S13B-G, the asymmetry of the temperature distribution for the samples with small dimensions is broken when the light power is strong enough, while the lager diameter and the hollow structure helps to reduce the negative effects.

2. Theory and simulation

2.1 Photo-thermal responsive behavior of the devices

The light triggered phototropic behavior of the actuating cladding is a multi-physics process.[1-3] For hydrogels, firstly, the light illuminates the photo-thermal responsive material, the temperature of the local region increases for the effect of the photo absorbers. Then the temperature increasement results in the change of the characteristics of the hydrogel network, when the temperature is over the LCST of the photo-thermal responsive hydrogels. The mass diffusion of water transfer out of the gel occurs at the same time, which triggers the large deformation of the cladding. When the infect of the illumination is turned off, the temperature drops below the critical temperature and the water transfers into the gel. From the explanation above, the heat transfer process involves the photo-thermal transfer process caused by the photo-absorbers and the heat convection of water and actuator surfaces. The mass diffusion process and large deformation properties can be expressed through the constitutive relations of the materials. Those two effects are modeled with the Solid Module and Heat Transfer Module in commercial multi-physics simulation software COMSOL.

Referring to the theory developed by Hong et al.,[1] the mechanical and mass diffusion performance can be modeled with the normalized nominal free energy density $\widehat{W}(\boldsymbol{F})$, which is given by:

$$\widehat{W}(\boldsymbol{F})\cdot v = \frac{1}{2}N\nu[F_{iK}F_{iK} - 3 - 2\log(\det\boldsymbol{F})] - \left[(\det F - 1)\log\left(\frac{\det\boldsymbol{F}}{\det\boldsymbol{F} - 1}\right) + \frac{\chi(T)}{\det\boldsymbol{F}}\right] - \mu(\det\boldsymbol{F} - 1) \quad \text{(S1)}$$

$N\nu$ is the normalized shear modulus of the dry polymer and can be calculated with $N_{kT} = \frac{k_B T}{v}N\nu$, where N_{kT} is the shear modules of the dry gel, k_B is the Boltzmann constant, v is the molecule volume of water and T is the temperature of the surrounding environment. $\boldsymbol{F}$ is the deformation gradient. χ is the temperature dependent dimensionless materials properties representing the enthalpy of mixing, which can be calculated with the experimentally tested swelling ratio λ with the following equation.

$$0 = \frac{N\nu}{\lambda^2}\left(\lambda - \frac{1}{\lambda}\right) + \ln\left(1 - \frac{1}{\lambda^3}\right) + \frac{1}{\lambda^3} + \frac{\chi(T)}{\lambda^6} \quad \text{(S2)}$$

The normalized chemical potential μ can be calculated by the mass conservation equation:

$$\frac{1}{\det\boldsymbol{F}}\frac{\partial[\det\boldsymbol{F} - 1]}{\partial t} + \frac{\partial\left[-\frac{[\det\boldsymbol{F} - 1]D(T)}{\det\boldsymbol{F}}\frac{\partial\mu}{\partial x_i}\right]}{\partial x_i} = 0 \quad \text{(S3)}$$

where $D(T)$ is the temperature dependent intrinsic diffusivity and can be obtained by fitting the transient process with the present model.

The thermal effect generated by the photo-absorber is simplified with the presetting of Deposited Beam Power node in COMSOL. The heat transfer process can be modeled with

$$\rho C_P\frac{\partial T}{\partial t} + \nabla(-k\nabla T) = 0 \quad \text{(S4)}$$

ρ is the density of the material, C_P is the specific heat capacity of the hydrogel and k is the thermal conductivity of the hydrogel. C_P can be calculated with

$$C_P(\boldsymbol{F}) = \frac{1}{\det F}C_{P_{drygel}} + \left(1 - \frac{1}{\det F}\right)C_{P_{water}} \quad \text{(S5)}$$

$C_{P_{drygel}}$ is the specific heat capacity of dry gel and $C_{P_{water}}$ is the specific heat capacity of water.

The heat convection equation is

$$q = h(T_{water} - T) \tag{S6}$$

h is the heat transfer coefficient of the solvent-hydrogel boundary, T_{water} is the temperature of the surrounding water.

The theory model was verified with the demo that a PNIPAAm hydrogel column bending towards the obliquely incident light. The experiment and simulation results are shown in Figure S12.

The theory model is used for the analysis of different deformation processes under outside illumination and inside illumination (Figure 2H). Parameters used in the simulation are shown in table S3.

The theory model is also used for the analysis of the five ports logic element (Figure S45). In this case, the geometry model is a hollow cylinder with the bottom fixed and restrained. Input lasers are set from different direction respectively. Parameters used in the simulation are shown in table S4. The simulation results are shown in Figure S46, with the schematic drawing and the temperature distribution with deformation including the upper view and overall view. From the results, all the deformation simulations accord with the design principle. The basic element is conducting when the input combination is A, C and D on, B off, or A, B and C off, D on.

2.2 Light propagating within the responsive waveguide

We simulated the light propagating process within the responsive waveguide with the Geometrical Optics Module of COMSOL. Ray trajectories and the transmittance of the inner layer was analyzed. The geometry model of the waveguide is a bending double layer cylinders (Figure S11A). The transmittance of the light through the inner layer is measured by using a wall node with the Deposited Ray Power sub-node to compute the total incident power that arrives at the end of the waveguide.

Figure S11B shows the ray trajectories of internal illumination. Most of the ray passes through the core of the waveguide and the transmittance is 0.927. Parameters used in the simulation of internal illumination are shown in table S5. For external illumination, we simulated the situation when the boundaries of incident light and waveguide's external surface are tangent. Part of the light is shadowed by the cladding and the rest part of the illumination transmits through the core (Figure S11C). Transmittance of the device was calculated with different diameter of the light varying from 0.2 to 2 mm (Figure S11D). Parameters used in the simulation of external illumination are shown in table S6.

2.3 The limitation of size caused by thermal transport

Besides fabrication constraints, size exerts significant influence on thermal transport which influences the performance of the photothermal system. For the proposed methodology, establishing an effective temperature gradient across the structural section constitutes the critical determinant for functional realization. We evaluate geometric limitations through the analysis of thermal diffusion length $\delta = \sqrt{2k/\rho c\omega}$, where k is the thermal conductivity of the actuator, ρ is the density, c is the specific heat, and ω is the frequency.[4] When the geometric size is within the thermal diffusion length, heat transfer significantly through the whole structure limiting the generation of temperature gradient and the light-induced asymmetry will be rapidly reduced. For 30 ms which is the response time of micron-scale structure proposed by Xin[5] et al., the calculated

thermal diffusion length is 86 μm. We compare the heat transfer behavior of column actuators with diameters of 500 μm and 5 μm, discovering significant temperature gradient for the large diameter actuator and tiny temperature difference for the small one (Figure S22). Also, there are several alternative approaches for facilitating effective responses at smaller scales. For instance, utilizing materials with distinct thermo-responsive properties can maintain asymmetric volume variations through inherent differential responsiveness,[6,7] even in the absence of sufficient temperature gradients, overcoming heat transfer limitations.

2.4 Theoretical Amplification Factor Calculation for Transistors

Our transistor is designed to modulate high-power photon signals using low-power photon signals. In the device design, assuming no core layer loss, the incident light source is regarded as entirely excluded. In an ideal small-sized transistor, once the device deviates from the source by a slight angle (<1°), signal transmission is interrupted. When the transistor is in its operational equilibrium state, $P_1 \cdot \sin\theta = P_2$, allowing the device to exert control. The source power is P_1, while the control light power is P_2. Under these conditions, the amplification factor is given by $\beta = P_1/P_2 = 1/\mathrm{Sin}\theta$. When $\theta = 1°$, the amplification factor β is 57.3. When $\theta = 0.5°$, the amplification factor β is 114.6.

When accounting for the absorption rates at the source and gate positions, expressed as $\alpha_1 \cdot P_1 \sin\theta = \alpha_2 \cdot P_2$, the amplification factor becomes $\beta = P_1/P_2 = \alpha_2/\alpha_1 \cdot \sin\theta$, leading to an even greater theoretical amplification when α_2 is higher than α_1.

If the transistor is designed not strictly in a symmetric form but instead adopts a locally deformable architecture, localized optical stimulation can be applied to achieve effective control.

Ideally, we assume the ambient fluid temperature is close to the LCST of the material, with a small temperature difference of 0.1 K. Define the photothermal responsive region with an area of 1 mm². When the control light power satisfies $P1 = hA\Delta T$, the device is effectively actuated. In a laminar flow water environment with a small temperature difference, the convective heat transfer coefficient is defined as h = 200, resulting in P1 = 2 mW. When the Source light power P2 is 2 W, the transistor achieves an amplification factor of $\beta = P1/P2 = 1000$.

Strictly speaking, by initially shielding the Source light properly to prevent its direct transmission, and regulating it through a small Gate input, the transistor can achieve an extremely high amplification factor.

3. Phototropic bending performance of actuators with different environmental temperature and humidity

As all hydrogel-based devices discussed in this work operate in an aqueous environment, external humidity does not directly influence the response of hydrogels immersed in water. To demonstrate that our proposed method is not limited to specific environments, we also present implementations of the LCE-based devices in an air environment. For the LCE structure, we tested its response under different humidity of 23%, 36% and 49% (Figure S17B). The actuator maintained consistent performance, indicating that humidity is not a dominant factor in LCE actuation.

Regarding temperature effects, we examined the bending response of PNIPAAm hydrogel at water temperatures of 11 ℃, 16 ℃, 21 ℃, 33 ℃, and 42 ℃ (Figure S17A). We compared the time required for 10° response and recovery for each condition to reveal the response speed and recovery speed. When the temperature exceeded the LCST (LCST for PNIPAAm hydrogel prepared with ice template method is 34℃ (Figure S2A)), the actuator had already undergone the volume change and the deformation triggered by illumination was limited. When the environment temperature was below LCST, the actuator showed a faster response speed with relatively higher temperature with the 10° response time decreasing from 2.52 s to 0.62 s while the temperature increased from 11 ℃ to 33 ℃. Only minor changes occurred for the recovery speed during the temperature variation and the 10° recovery times were under 1 s for all validated conditions within the LCST. Therefore, appropriately increasing the ambient temperature can reduce the energy threshold required for the responsive material to reach the transition temperature, while the ambient temperature should be maintained below the LCST to ensure sufficient deformation capacity.

4. Two configurations of the diode and the influence of the light diameter

In the main text, we introduced a diode design method based on an optical waveguide-driven architecture. However, for the same architecture, the implementation approach for diode functionality is not unique. For a diode fabricated and packaged using the method described in Figure S19, we consider the following three operational modes: 1) Up-side: Incident light enters from the top and interacts with the outer surface of the actuator (Figures S27C-D). 2) Down-side-ON: Incident light enters from the bottom port and interacts with the inner surface of the actuator (Figure S27C). 3) Down-side-OFF: Incident light enters from the bottom port but propagates directly through the inner layer of the driving structure (Figure S27D). For these cases, we tested the output responses of the LCE-based diode and observed significant differences in output among the three modes (Figure S28A) under the same input power of 129 mW. In the Down-side-ON mode, a substantial amount of light is emitted from the interior of the driving structure, achieving an output of 13.95 mW. In the Up-side mode, unavoidable non-conductive regions exist between the incident light and the inner light-guiding layer, resulting in a lower output of 0.1623 mW compared to the Down-side-ON mode. In the Down-side-OFF mode, the incident light does not directly interact with the driving structure, and the emitted light signal from the end of the driving structure is blocked by the encapsulation light-shielding film, resulting in no effective output and the output power detected was 0.0076mW. The diode design method discussed in Figures 3 and S27C employs the Down-side-ON mode as the forward output and the Up-side mode as the backward output. For the LCE-based diode, the isolation ratio was 19.34 dB, and in this configuration, a smaller output in the Up-side mode corresponds to better isolation characteristics. Similarly, when comparing the Down-side-OFF mode, the Up-side mode can also serve as the forward output, while a larger output in the Up-side mode indicates superior isolation properties. The isolation ratio of the LCE diode was 13.30 dB for this configuration.

The output efficiency of the diode in the Up-side mode is correlated with the diameter of the incident light and influence the performance of the two approaches. We consider two limiting occasions: 1) Spot light: If the incident light diameter is small (relative to the fixed geometric dimensions of the driving structure), the light cannot fully cover the non-conductive regions even when the structure aligns the incident direction and is entirely blocked by the walls (Figure S28B (left)), resulting in a theoretical output efficiency of 0. 2) Area light: If the incident light diameter expands to cover the entire driving structure, the inner light-guiding region is fully illuminated when the actuator aligns incident light (Figure S28B (right)). In this case, sufficient light can enter the guiding layer.

Thus, we conclude that in the Up-side mode, transmission increases from 0 as the incident light diameter grows (Figure S28C). For the structure under discussion, we identify the non-conductive regions as the actuating layer and define the thickness of these regions as the critical threshold of the incident light diameter (Figure S28C) for the two configurations' effectiveness. When the incident light diameter is smaller than the actuating layer thickness, the diode is only compatible with the configuration illustrated in Figure S27C. When the incident light diameter exceeds the actuating layer thickness, the diode accommodates both design methods in Figures S27C-D. Also, practical implementations may exhibit deviations due to specific material deformation and light-guiding conditions.

5. Quantitative analysis for the LCE-based logic element and discussions on multi-unit integration

Through variation of input powers at ports A (21 mW, 40 mW, 62 mW) and B (21 mW, 34 mW, 47 mW), we obtained the output characteristics of LCE logic units (Figure S40A). When maintaining constant input power at port A, increasing port B's power enhanced the actuator's deformation, progressively occluding the incident light at port A and consequently reducing the output (Figure S40B). At 47 mW of port B input, the output power approached zero, demonstrating isolation ratio exceeding 27.9 dB for all port A power levels (Figure S40C). Thus, we adopted 47 mW as the driving power for subsequent analyses. The signal-to-noise ratios (SNR) under various input power combinations are presented in Figure S40D. Increasing port A's power improved SNR in both ON/OFF states. During the ON state (zero port B input), the SNR depended solely on the device's transmission efficiency for port A signals and remained above 39 dB. As port B power increased, transitioning the unit toward OFF state, the SNR decreased correspondingly, confirming effective switching behavior. The transmission efficiency reached 75.68 ± 1.66% in the fully ON state (Figure S40E).

Having established the fundamental properties of the basic logic units, we focus on multi-unit configurations as well. Regarding cascadability, since the light modulation mechanism relies solely on optical path obstruction without altering light characteristics, one unit's output can directly serve as another's input. This is validated by successful implementations of AND, NOR, OR, and NAND gates using two or three units (Figures 5J-M). For fan-out capability, experimental verification confirmed that one unit's output could simultaneously drive two downstream units via beam splitter, demonstrating a minimum fan-out of 2 (Figure S41). Also, the bilayer hydrogel transistor units discussed earlier exhibited an amplification ratio of 5.96 (Figure 4I), theoretically enabling a fan-out of 5. For the LCE units, considering port B's driving power of 47 mW, the transition time from ON to OFF state was measured as 12 s, yielding an energy consumption of 0.56 J.

Supplemental Figures

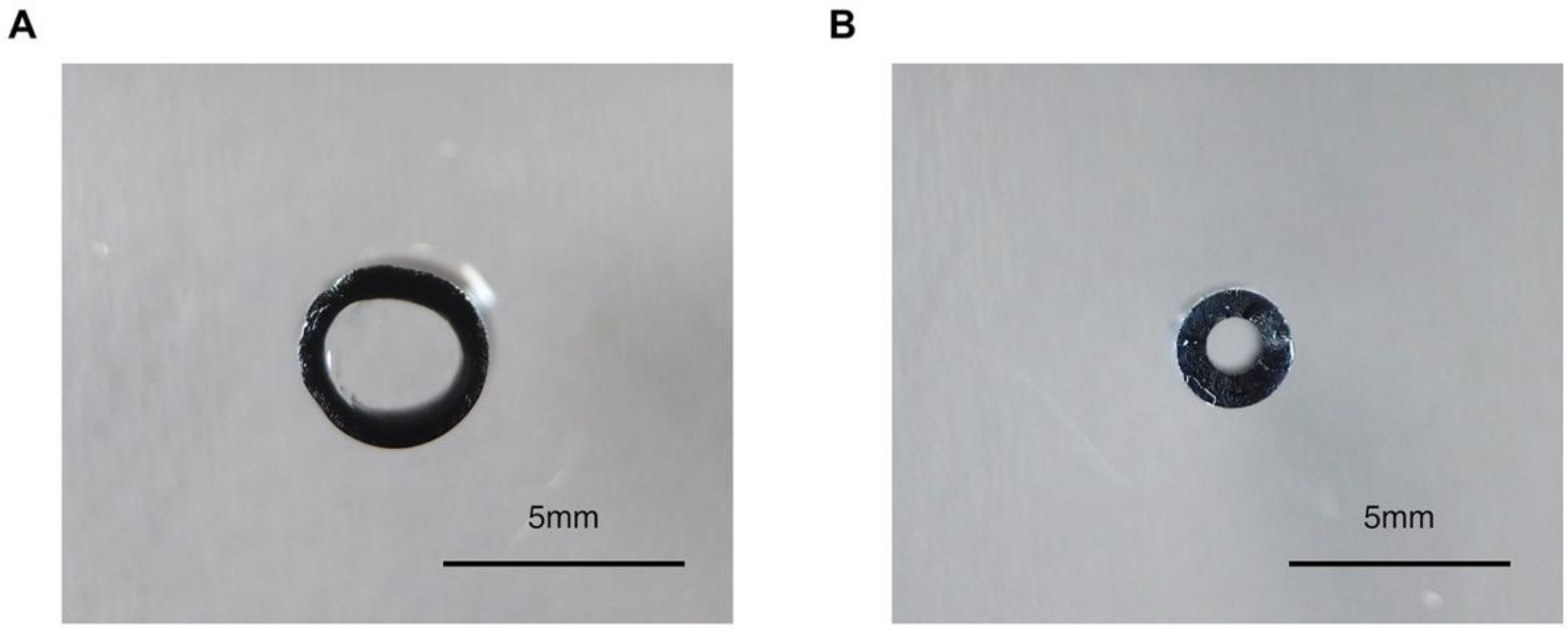

Figure S1. Cross section photos of double layer hydrogel and hollow RM257 LCE. (**A**) Cross section image of double layer hydrogel with PNIPAAm hydrogel as the outside layer and PAM hydrogel as the inside layer. (**B**) Cross section images of RM257 LCE cladding.

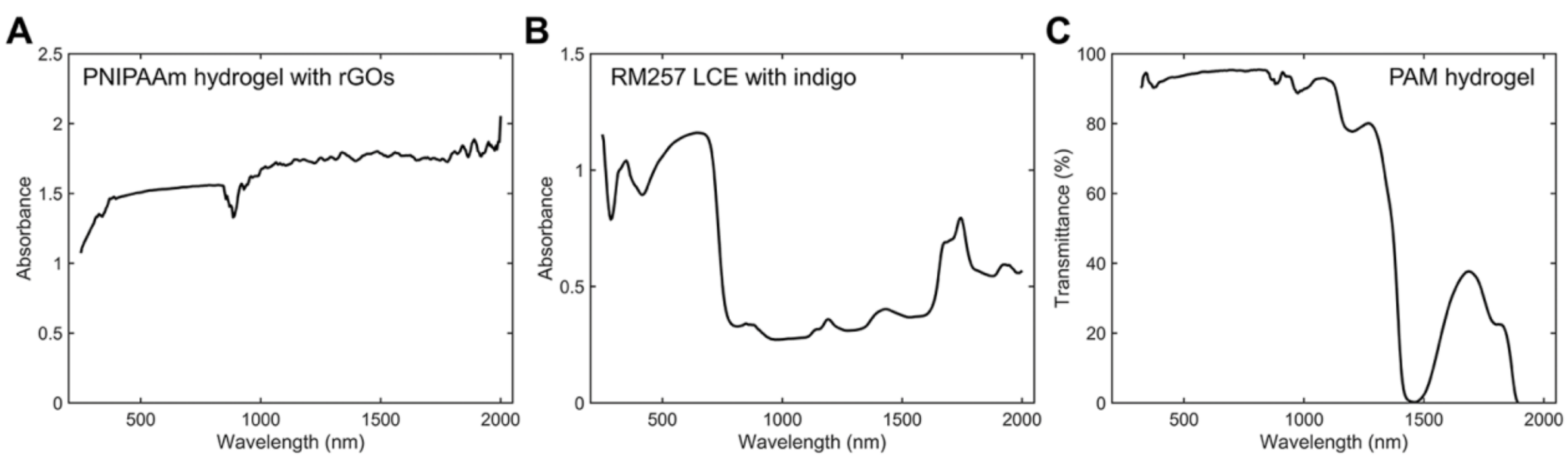

Figure S2. UV-Vis-NIR absorption spectrum for PNIPAAm hydrogel (A) and RM257 LCE (B) containing photo-absorber and transmittance spectrum for PAM hydrogel (C).

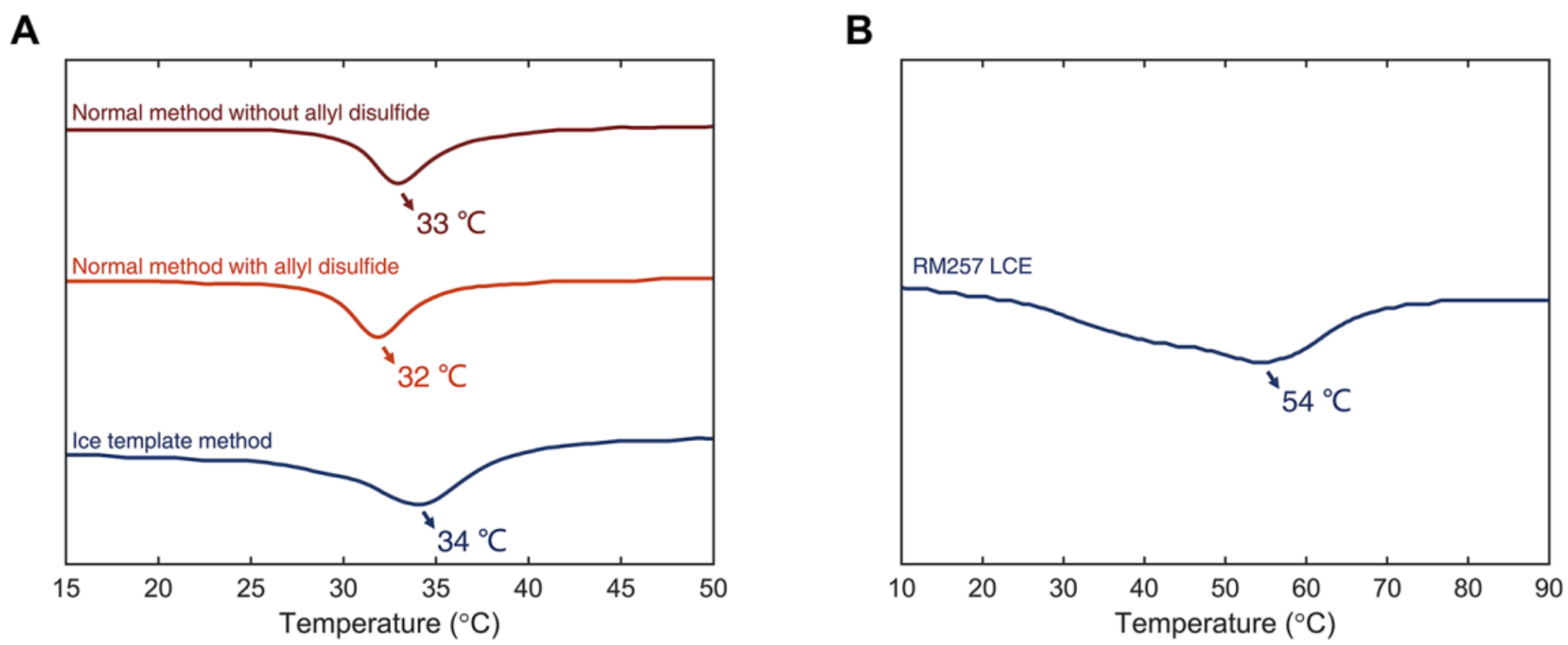

Figure S3. Differential scanning calorimetry curve. (**A**) The DSC results of PNIPAAm hydrogels. (From top to bottom, the hydrogels were prepared with normal method at room temperature without allyl disulfide, normal method with allyl disulfide and ice template method. The LCST are 33 ℃, 32 ℃ and 34 ℃ respectively.) (**B**) The DSC results of RM257 LCE. (TNI for RM257 LCE is 54 ℃.)

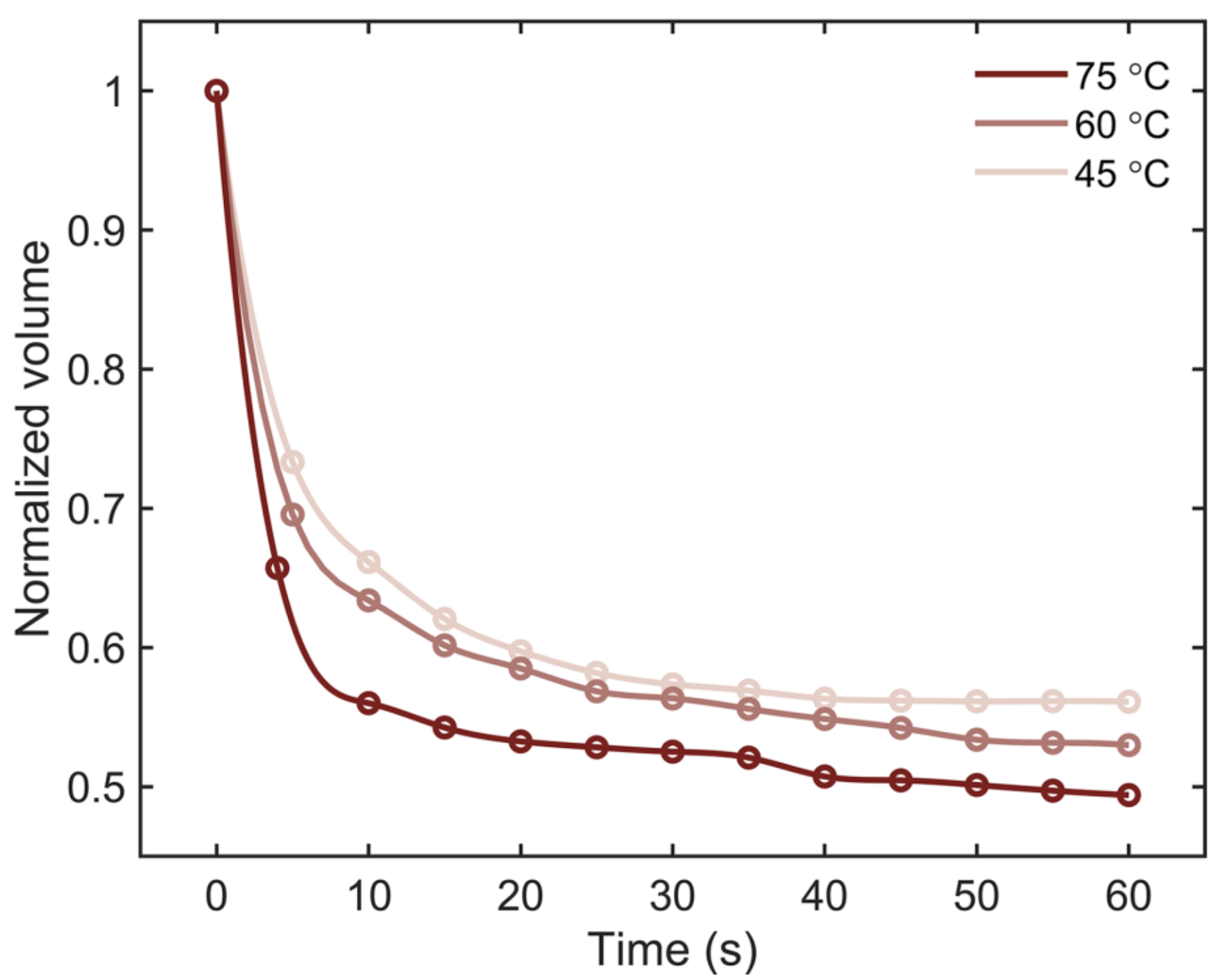

Figure S4. Swelling ratios and kinetics of the PNIPAAm hydrogel under the temperature of 45, 60, 75 ℃.

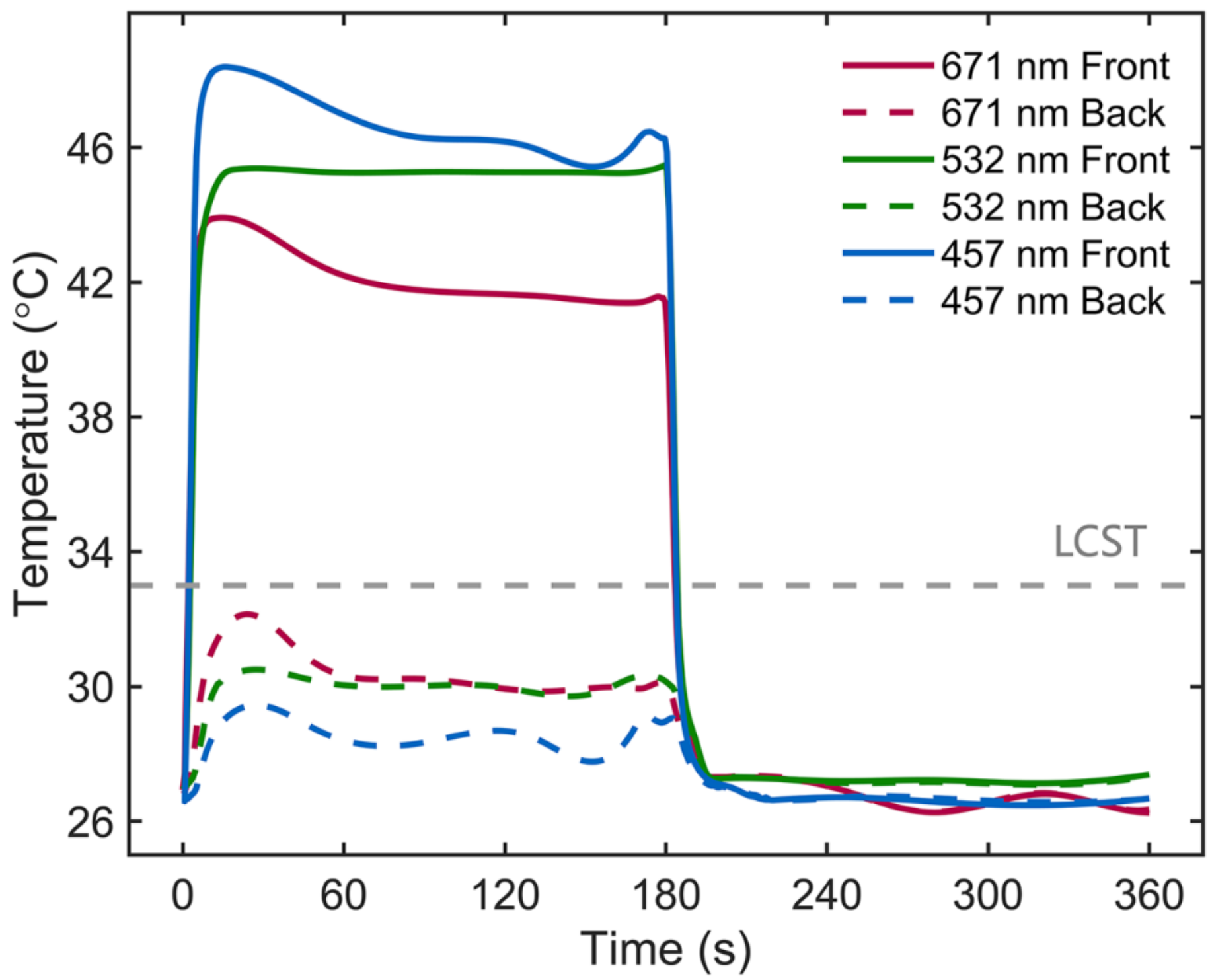

Figure S5. Time evolution of temperature detected by the thermocouples (Figure 2B) under the illumination on the outer surface. The red, green and blue curve correspond to the laser with wavelength of 671 nm, 532 nm and 457 nm. The solid lines correspond to the illuminated side and the dotted lines correspond to the back side.

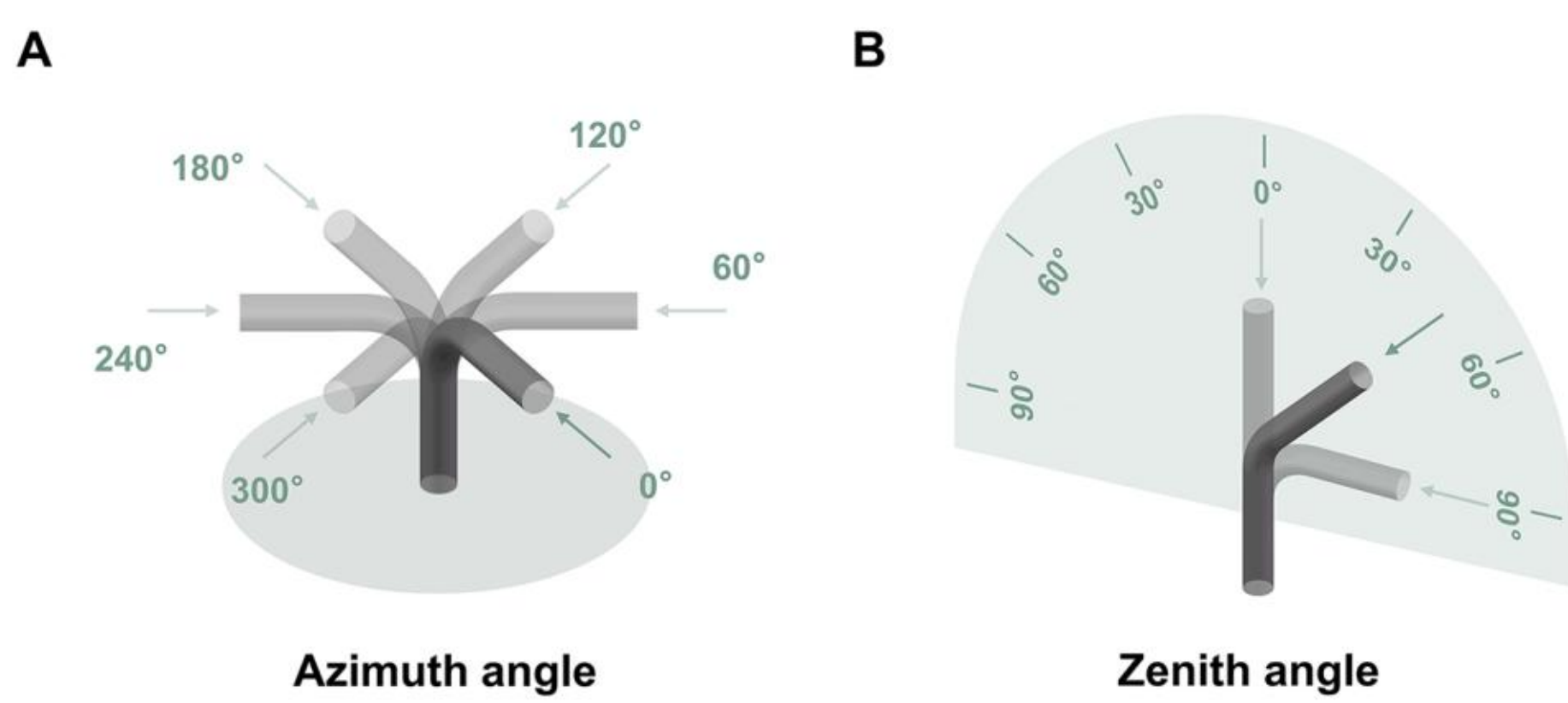

Figure S6. Schematic of azimuth angle (A) and zenith angle (B).

A

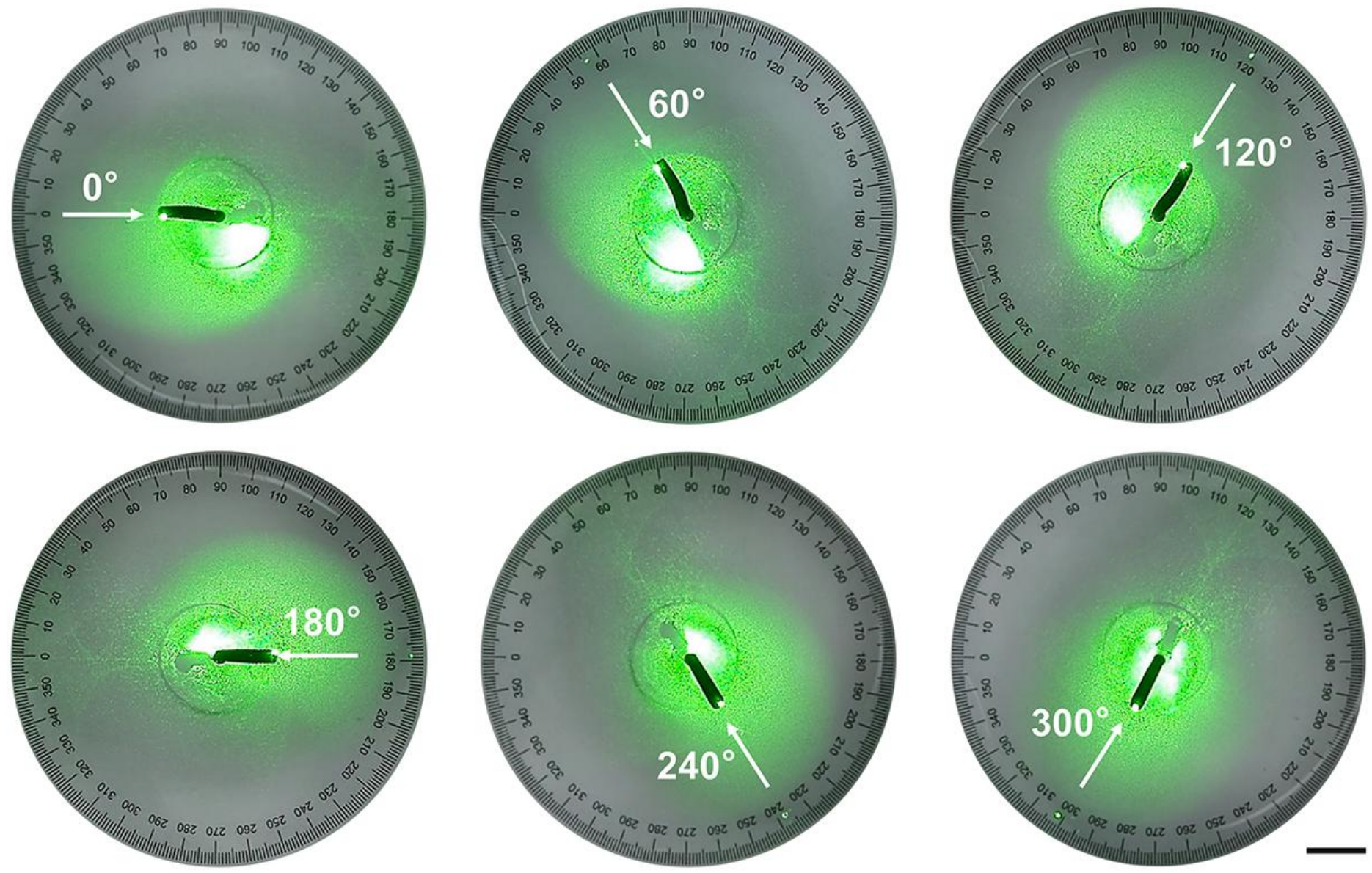

B

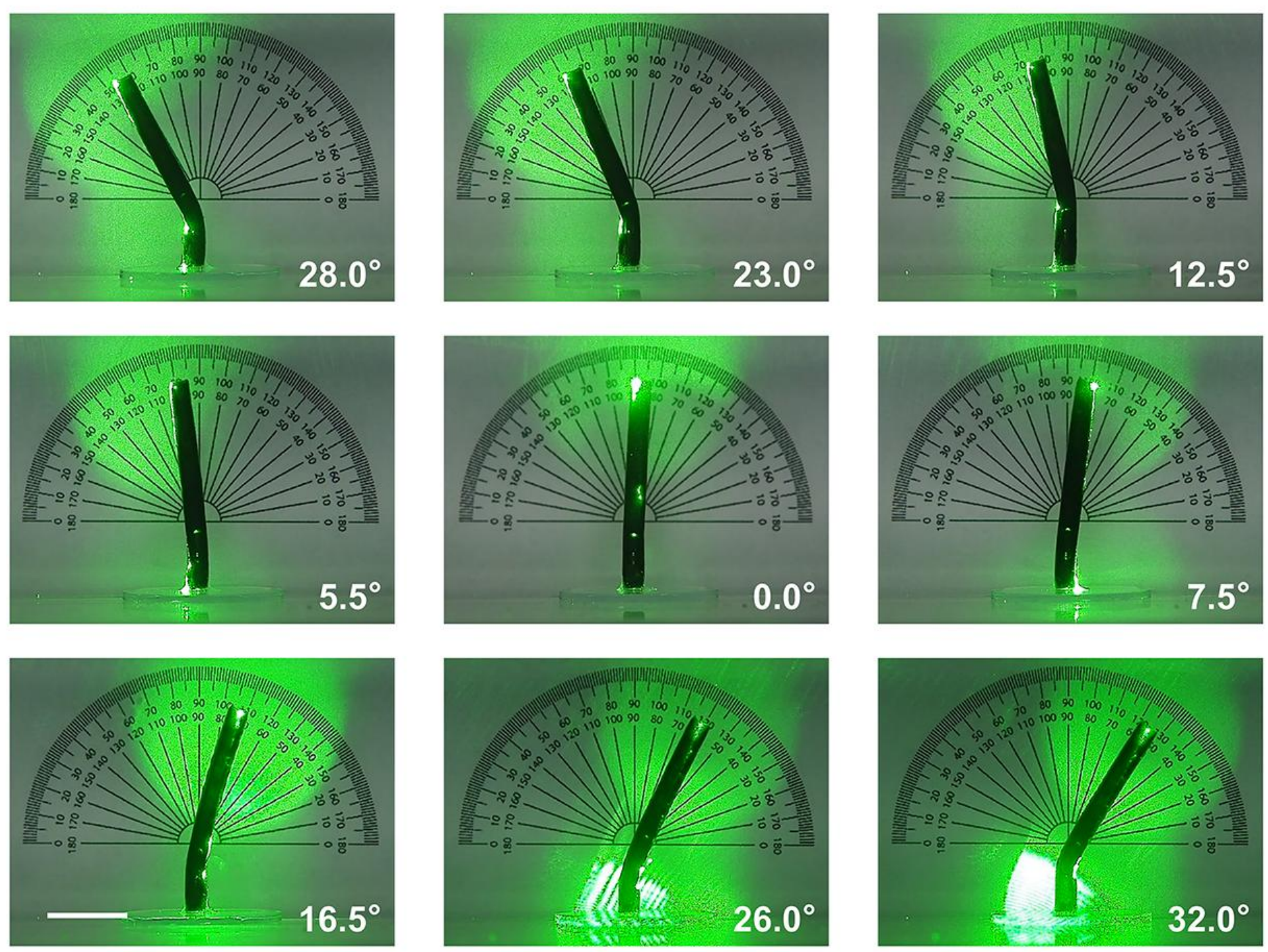

Figure S7. Omnidirectional phototropic bending of a PNIPAAm hydrogel cladding. (**A**)The azimuth angle varying from 0 ° to 360.0 °. The actuator was placed in the center of the rotating platform and the light was turned on when the direction of the platform had been adjusted. (**B**)The zenith angle varying from 0.0 ° to 32.0 °. Scale bars, 1 cm.

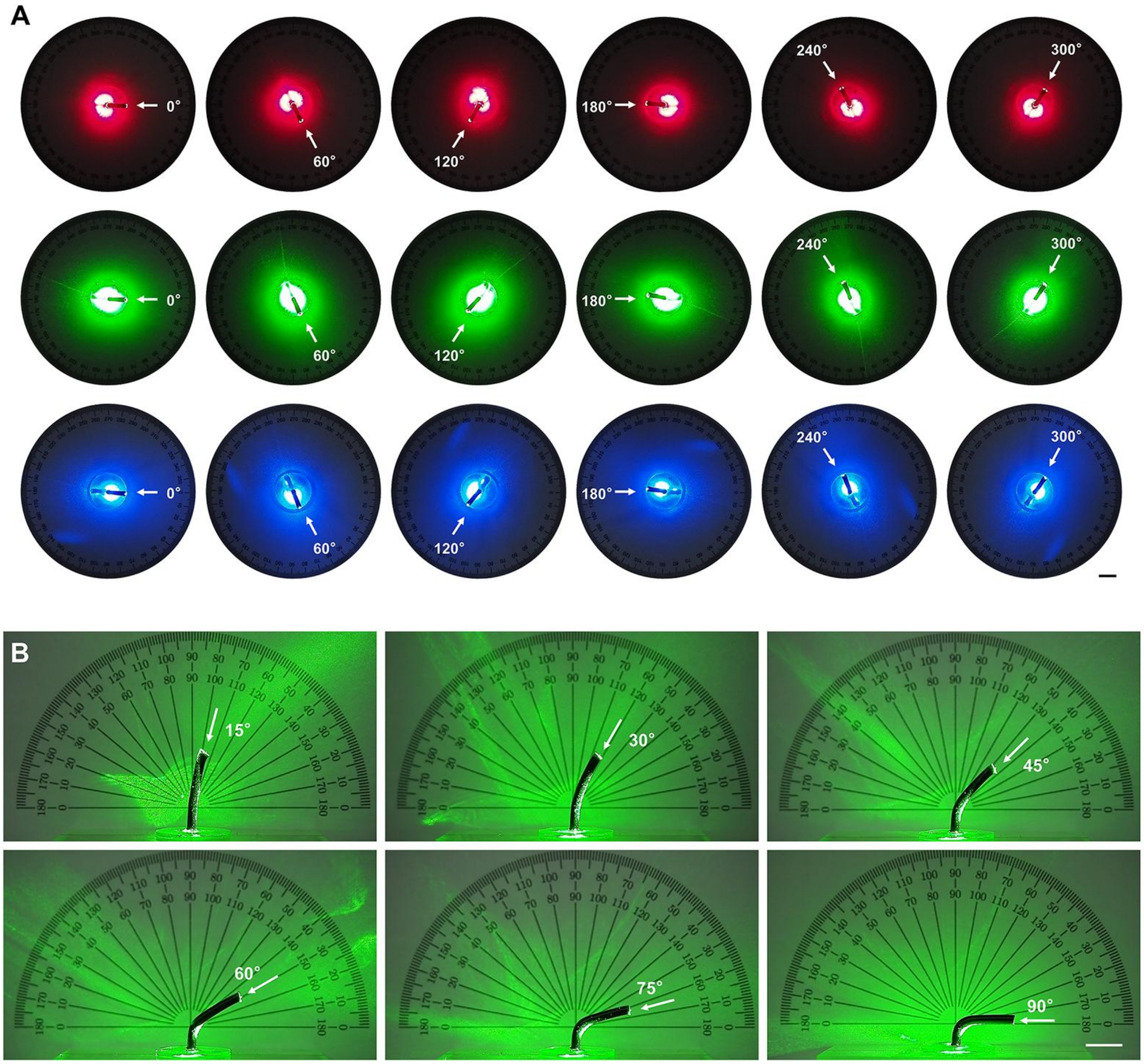

Figure S8. Omnidirectional phototropic bending of LCE cladding. (**A**) The azimuth angle varying from 0 ° to 360 ° under red, green and blue laser illumination. The illumination was shone from the fixed direction and the sample was placed vertically in the center of an electric rotating platform. The platform was turned for 60 ° every 30 seconds until it had turned 360 °. During that period of time the illumination was on consistently. (**B**) The zenith angle of 15 °, 30 °, 45 °, 60 °, 75 °, 90 °. The illumination was shone from different zenith angle, and the actuator could bend along with the incident direction eventually. Scale bar, 1 cm.

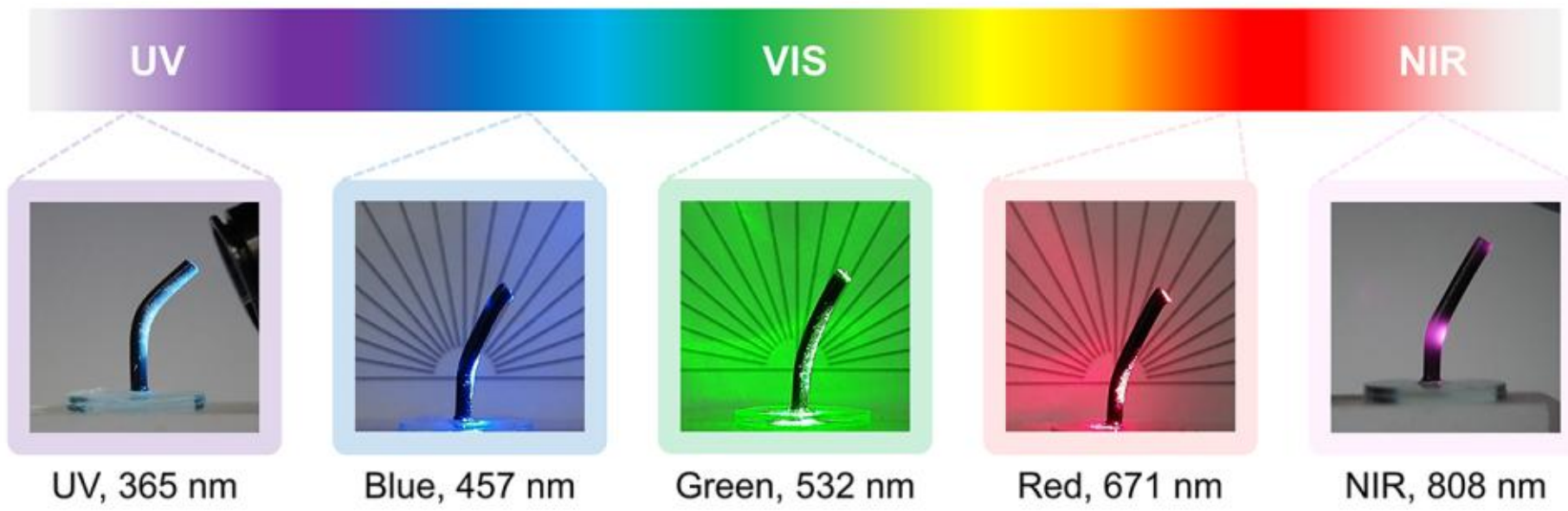

Figure S9. LCE actuator bending towards light with wavelengths across UV-Vis-NIR region.

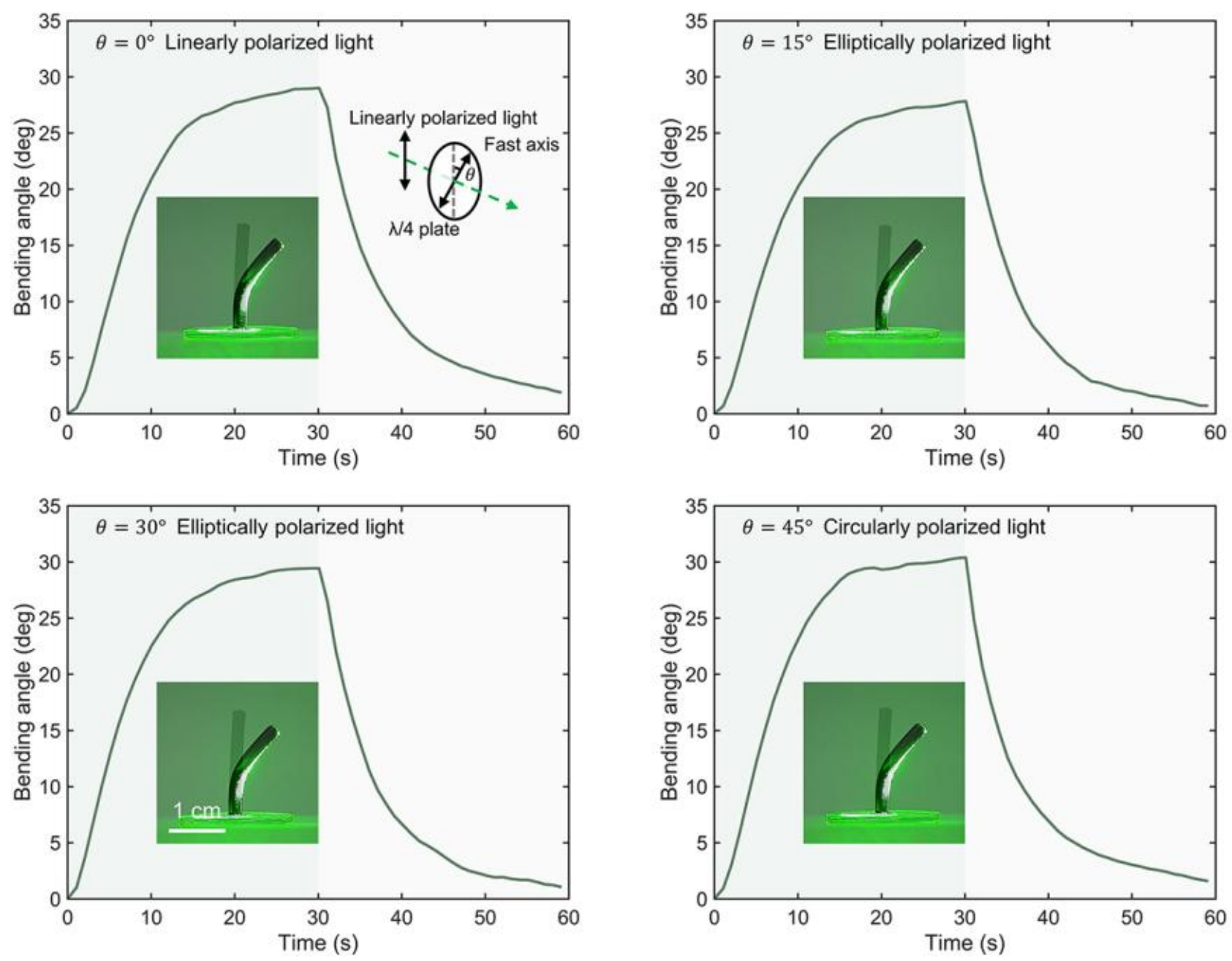

Figure S10. LCE actuator tracking the incident light with different polarization state including linearly polarized light, elliptically polarized light and circularly polarized light (light power, 150 mW).

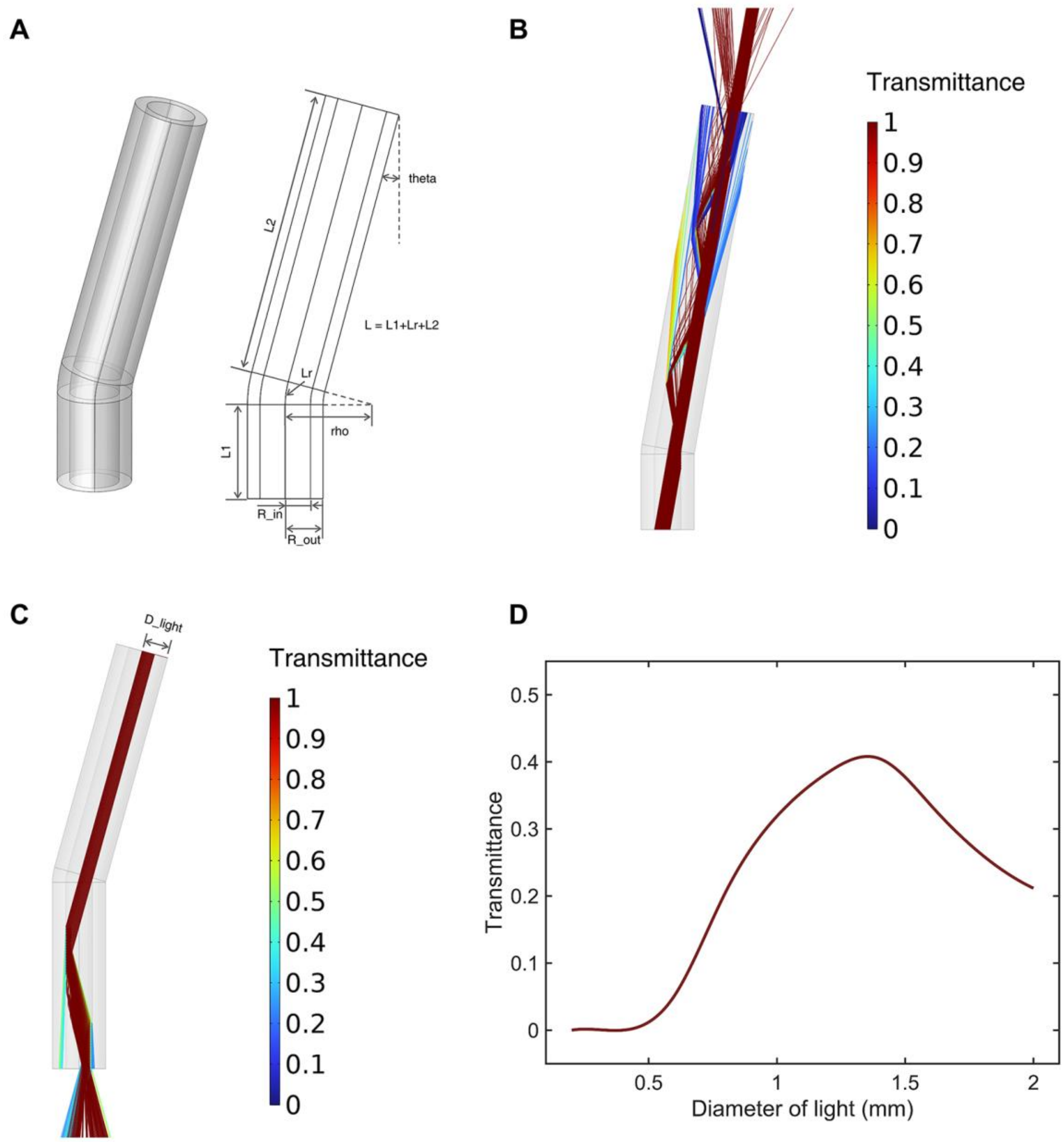

Figure S11. Simulation of the light propagating within the responsive waveguide. (**A**) The geometry structure and the parameters of the waveguide. (**B**) Ray trajectories of the waveguide under internal illumination and the color expression for the refracted rays indicates the transmittance. (**C**) Ray trajectories of the waveguide under external illumination. (The diameter of the light is 1 mm.) (**D**) The transmittance of the waveguide with light diameter varying from 0.2 to 2 mm.

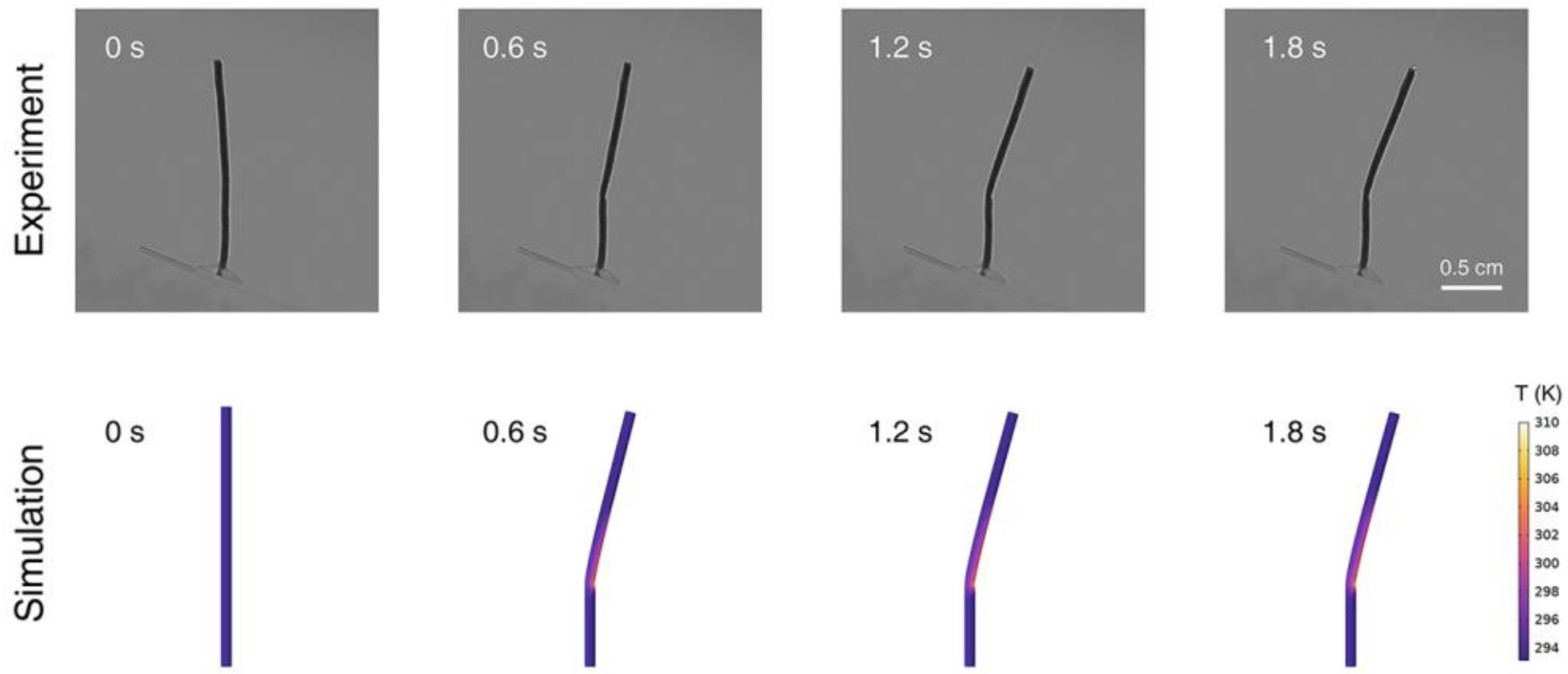

Figure S12. The experiment and simulation results of a PNIPAAm hydrogel column bending towards the obliquely incident light.

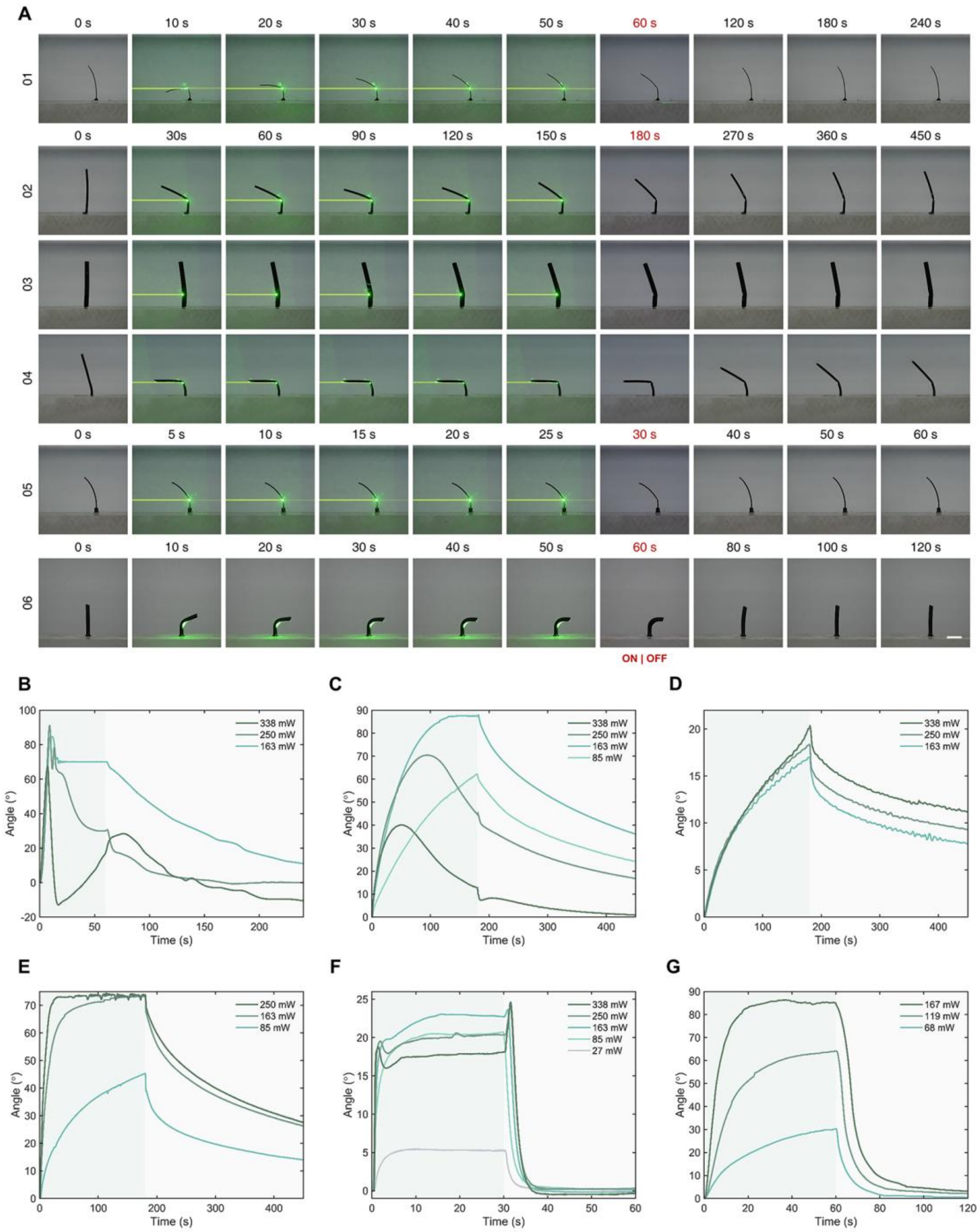

Figure S13. Phototropic bending of samples with different geometric parameters and materials. (**A**) Photos of the bending and recovery process of different samples. (The illumination direction is horizontal. The laser was turned on at 0 s and the time legend which is shown with red color represents the point when the laser was turned off. The number on the left is used to name different samples. The light power for sample 01-05 was 250 mW and for sample 06 is 167 mW. Diameters of sample 01-05 are 0.48 mm, 1.09 mm, 2.30 mm, 1.08 mm, and 0.66 mm respectively. Inner diameter of sample 06 is 1.03 mm and outer diameter is 2.33 mm.) (**B** to **G**) Time evolution of angle variation for sample 01-06. Scale bar, 1 cm.

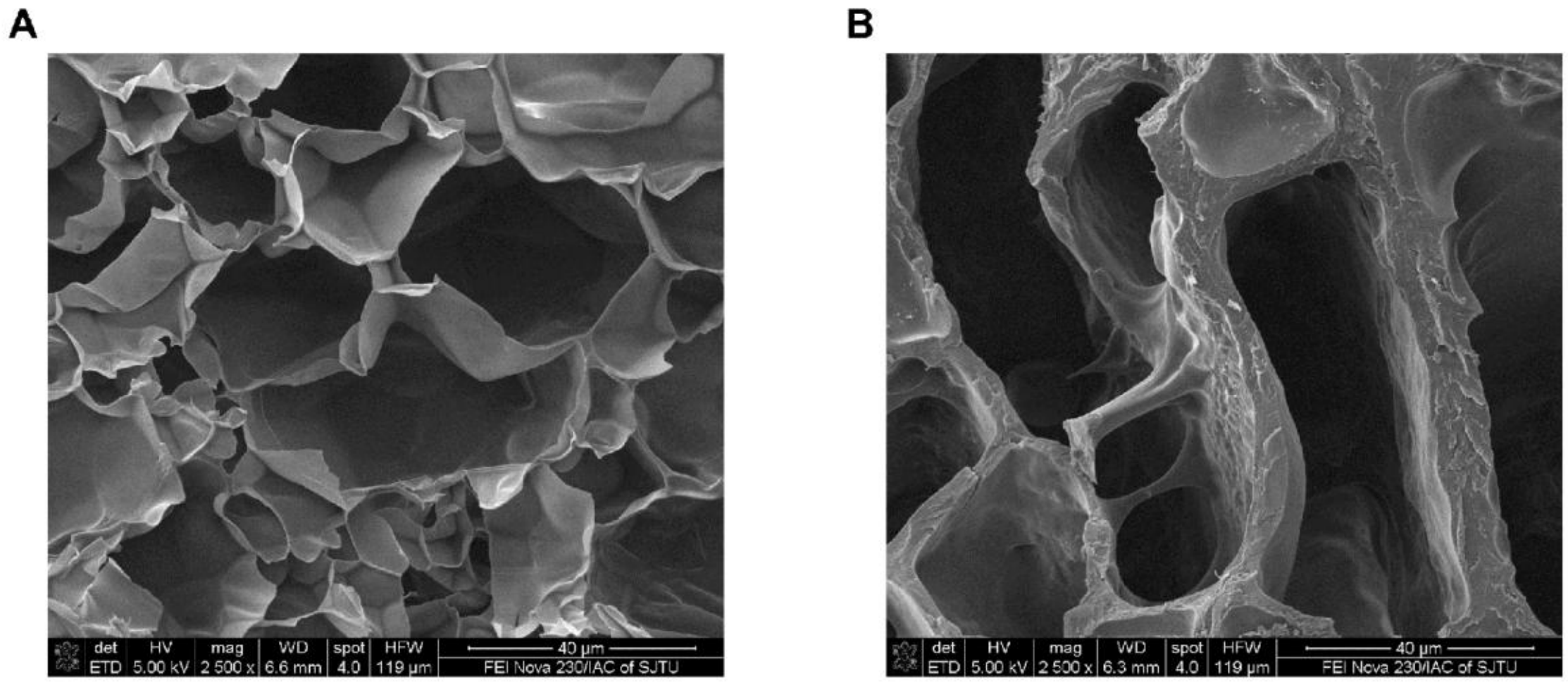

Figure S14. Scanning electron microscope images of PNIPAAm hydrogels. (**A**) Cross-sectional morphology of PNIPAAm hydrogel, prepared at room temperature with allyl disulfide. (**B**) Cross-sectional morphology of PNIPAAm hydrogel prepared with ice template method.

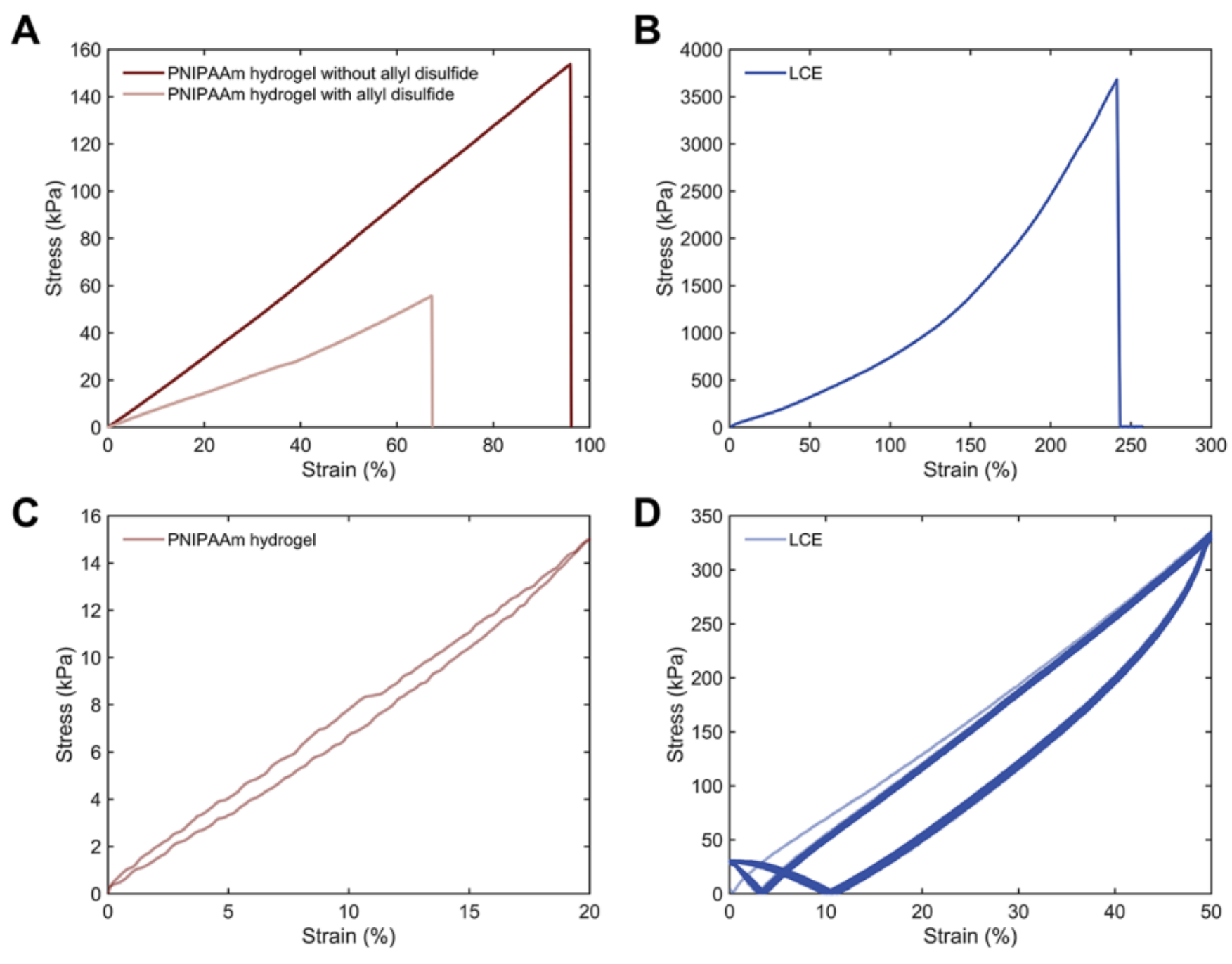

Figure S15. Stress-strain curves and cyclic tensile behavior of responsive materials. (**A**) Stress-strain curves of PNIPAAm hydrogels. (**B**) Stress-strain curves of RM257 LCEs. (**C**) Tensile behavior of PNIPAAm hydrogel (maximum strain, 20%). (**D**) Cyclic tensile behavior of RM257 LCE. (Maximum strain, 50%. Repeated for 50 cycles.)

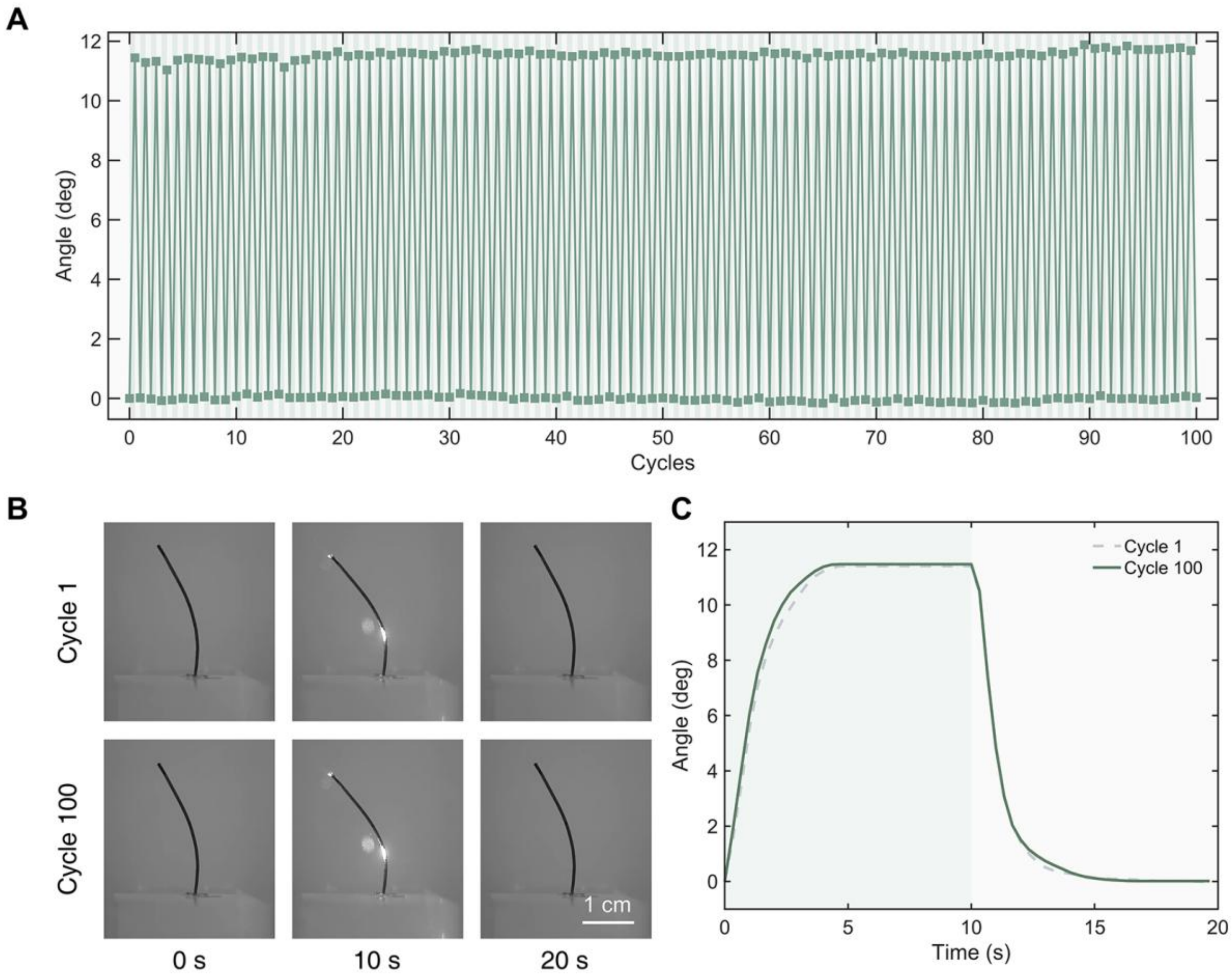

Figure S16. Cycling durability experiment of photothermal responsive hydrogel column. (**A**) Bending angle at the start and end of the cycle. (Light intensity was 51 mW and every cycle the light was on and off for 10 s.) (**B**) Motion of the sample at cycle 1 and cycle 100. (**C**) Time evolution of bending angle at cycle 1 and cycle 100.

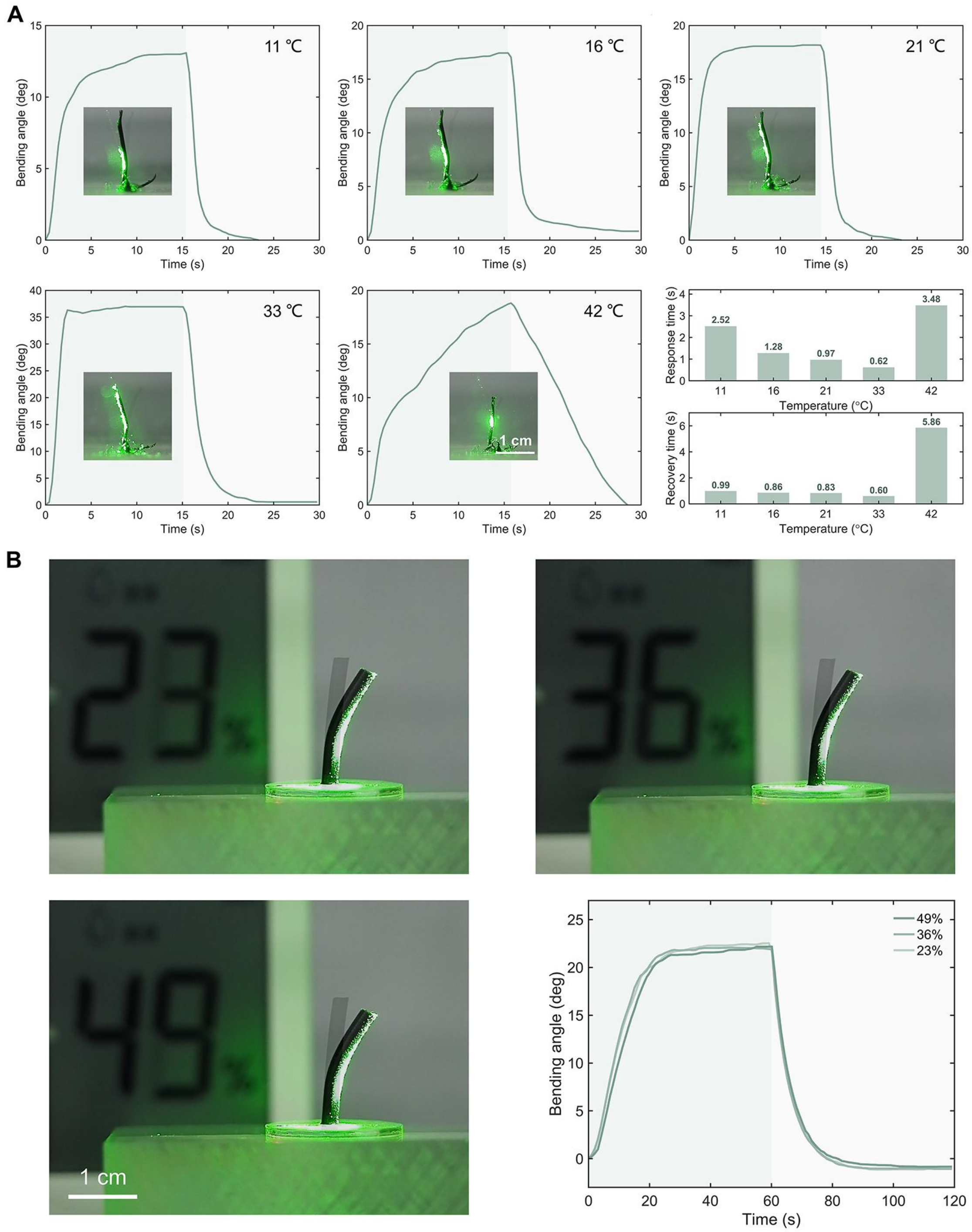

Figure S17. Phototropic bending behaviors with different environmental temperature and humidity. (**A**) Phototropic bending behaviors of PNIPAAm hydrogel-based actuator under temperature of 11 ℃, 16 ℃, 21 ℃, 33 ℃and 42 ℃ together with the 10° bending and recovery time (input power, 120 mW). (**B**) Phototropic bending behaviors of LCE-based actuator with humidity varying between 23%, 36% and 49% (input power, 140 mW).

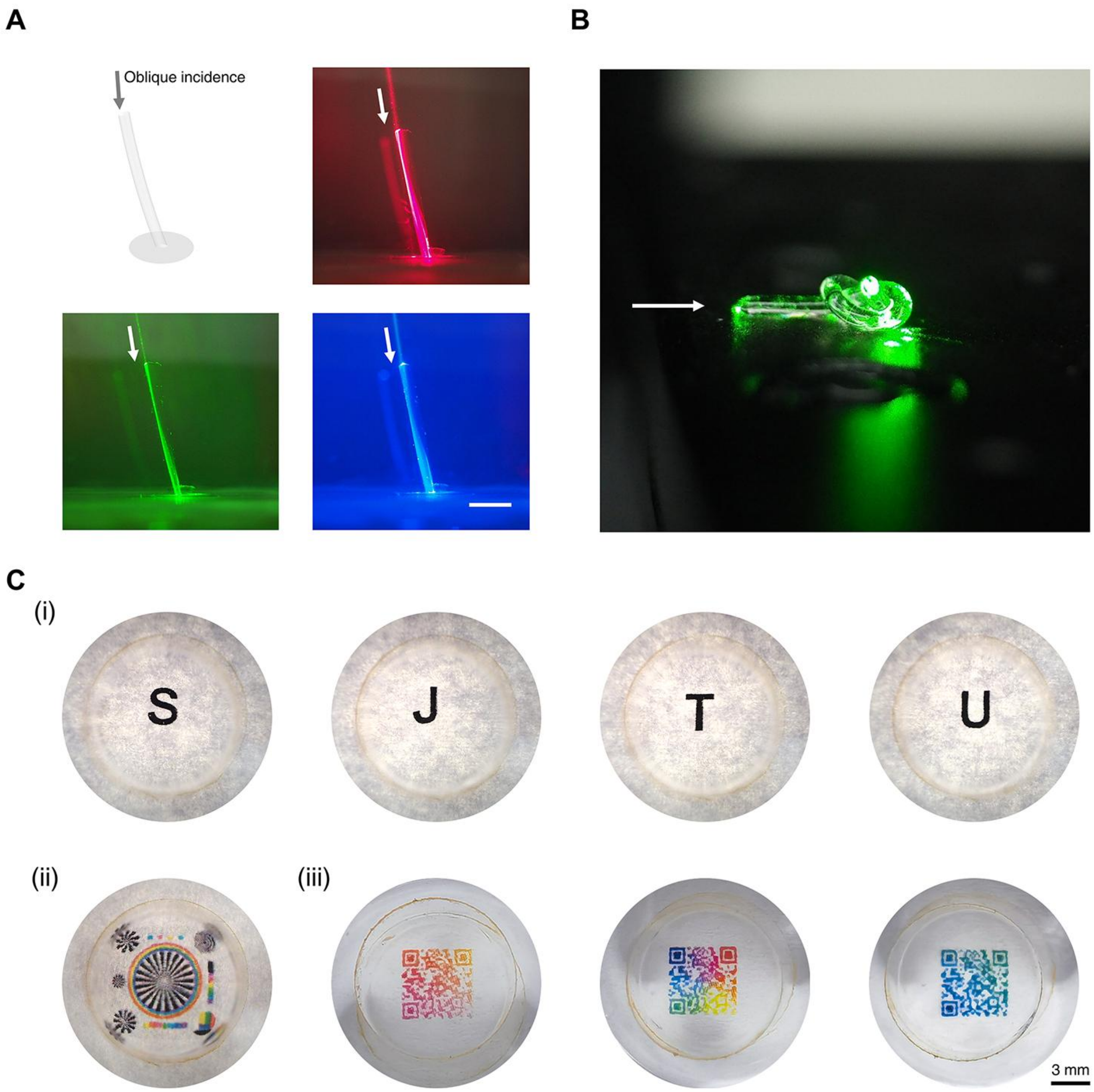

Figure S18. The light conductivity of PAM hydrogel. (**A**) Light guiding capacity of PAM hydrogels shown with the reflection of oblique incident light at the interface between water and hydrogels. (**B**) A knotted PAM hydrogel column guiding the laser shinning from the left side. The illumination entered from the left side and from the end of the PAM hydrogel, a bright spot can be observed. The results verify the capacity of PAM hydrogel to guide light with a bending structure. (**C**) Images of different patterns captured through the PAM hydrogel column. Paper sheets with different patterns were put under a glass container, where the PAM hydrogel column was set vertically inside. Letters 'S J T U' (i), a focus chart (ii) and a QR code with different colors (iii) were used as the patterns, indicating the diversity of the information which can be transferred with the device.

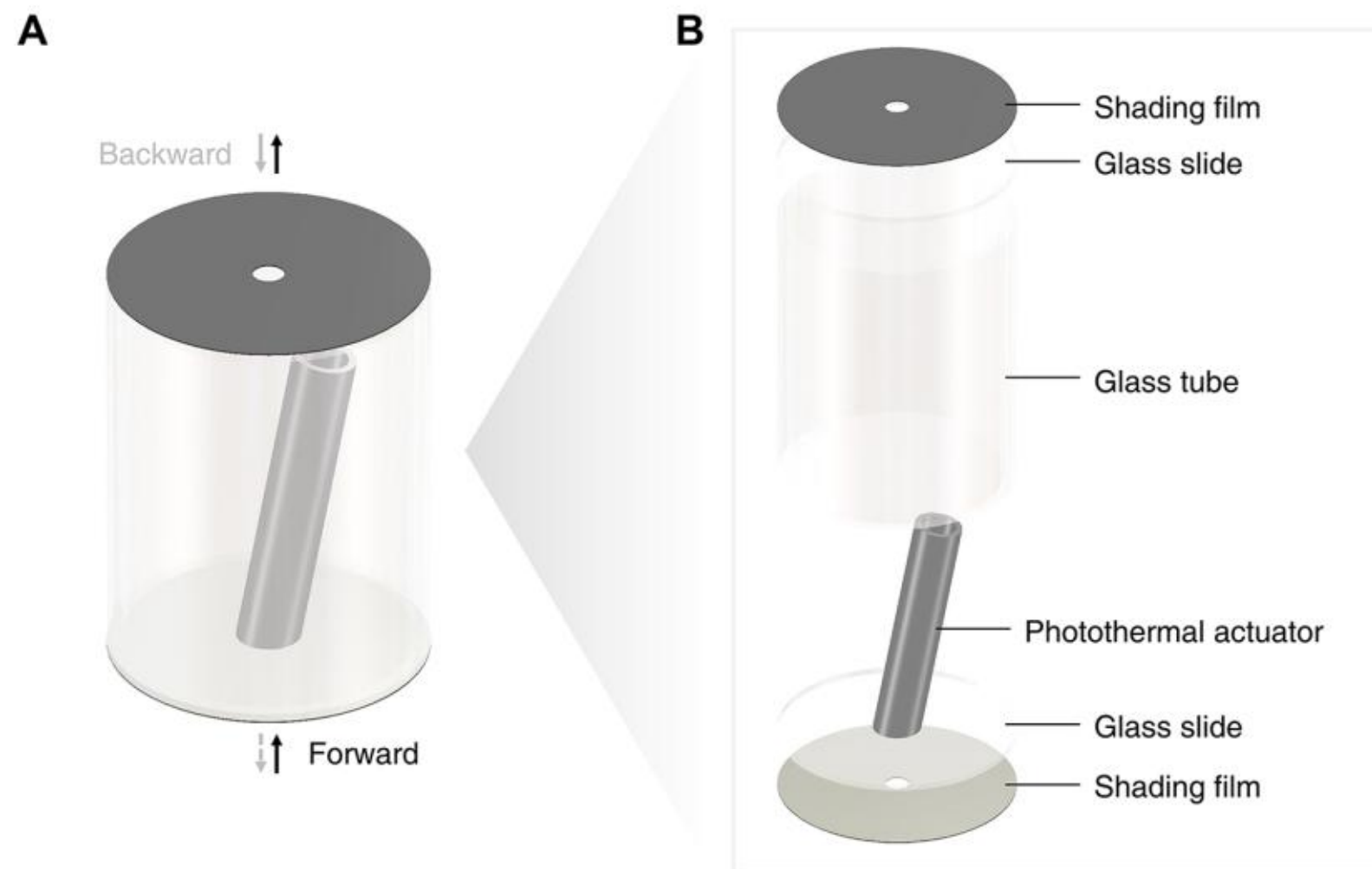

Figure S19. Schematic of the diode. (**A**) The structure and the operating modes of the diode. (**B**) The assembly and packaging methods of the diode.

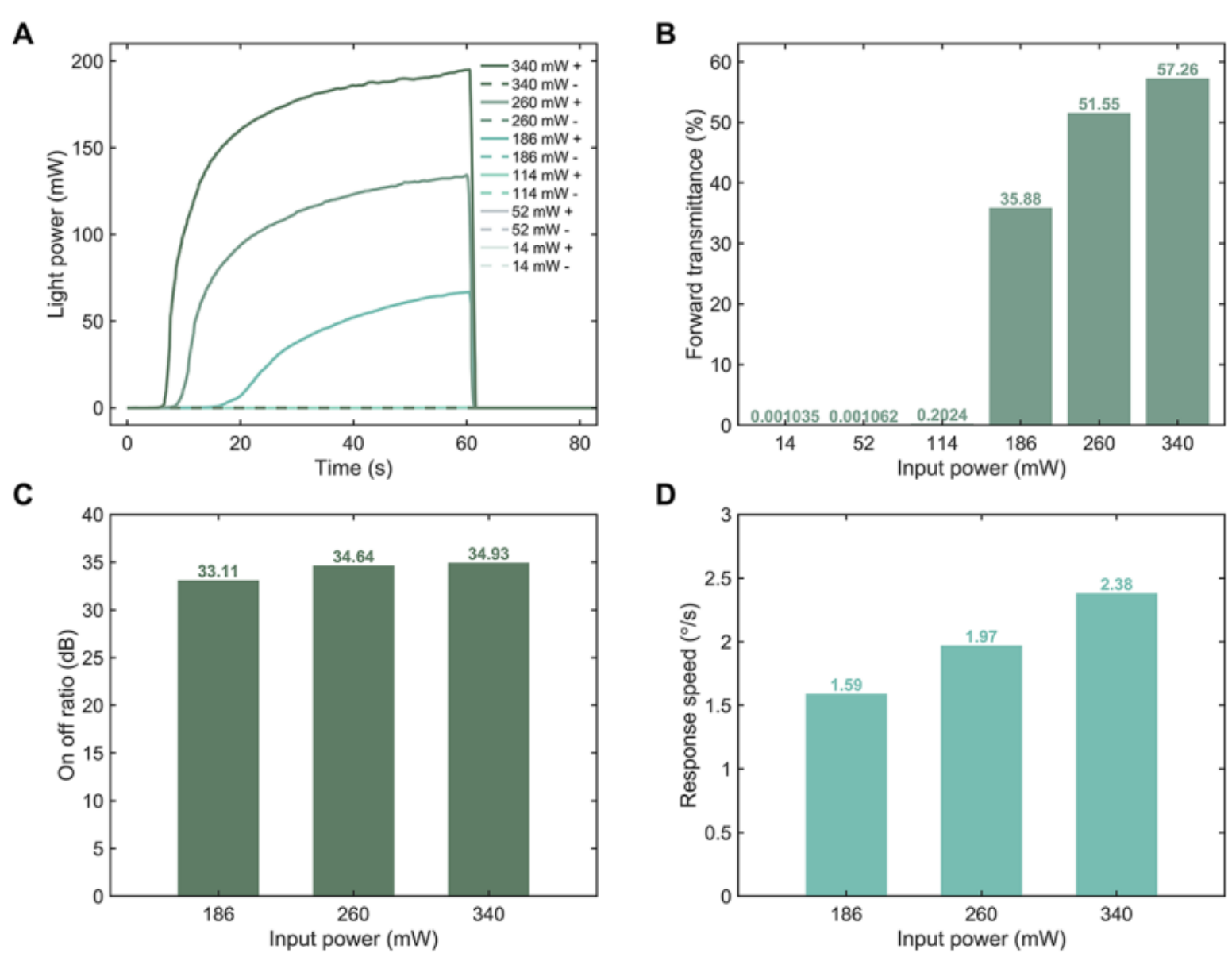

Figure S20. The characteristics of the diode made from room temperature PNIPAAm hydrogel which is introduced in Figures 3D-F, with 1.2 mm inner diameter and 2.5 mm outer diameter. (**A**) Time evolution of output power for different power of incident light from forward and backward directions. (**B**) Forward transmittance of the diode. When the input power reaches 186 mW, the forward transmittance increases immediately and the diode enters the operation window. (**C**) The isolation ration of the diode when the diode is in working condition. When the diode starts to work, the isolation ratio remains above 33 dB. (**D**) The response speed of the diode when the diode is in working condition. The maximum response speed increases when the input power is stronger.

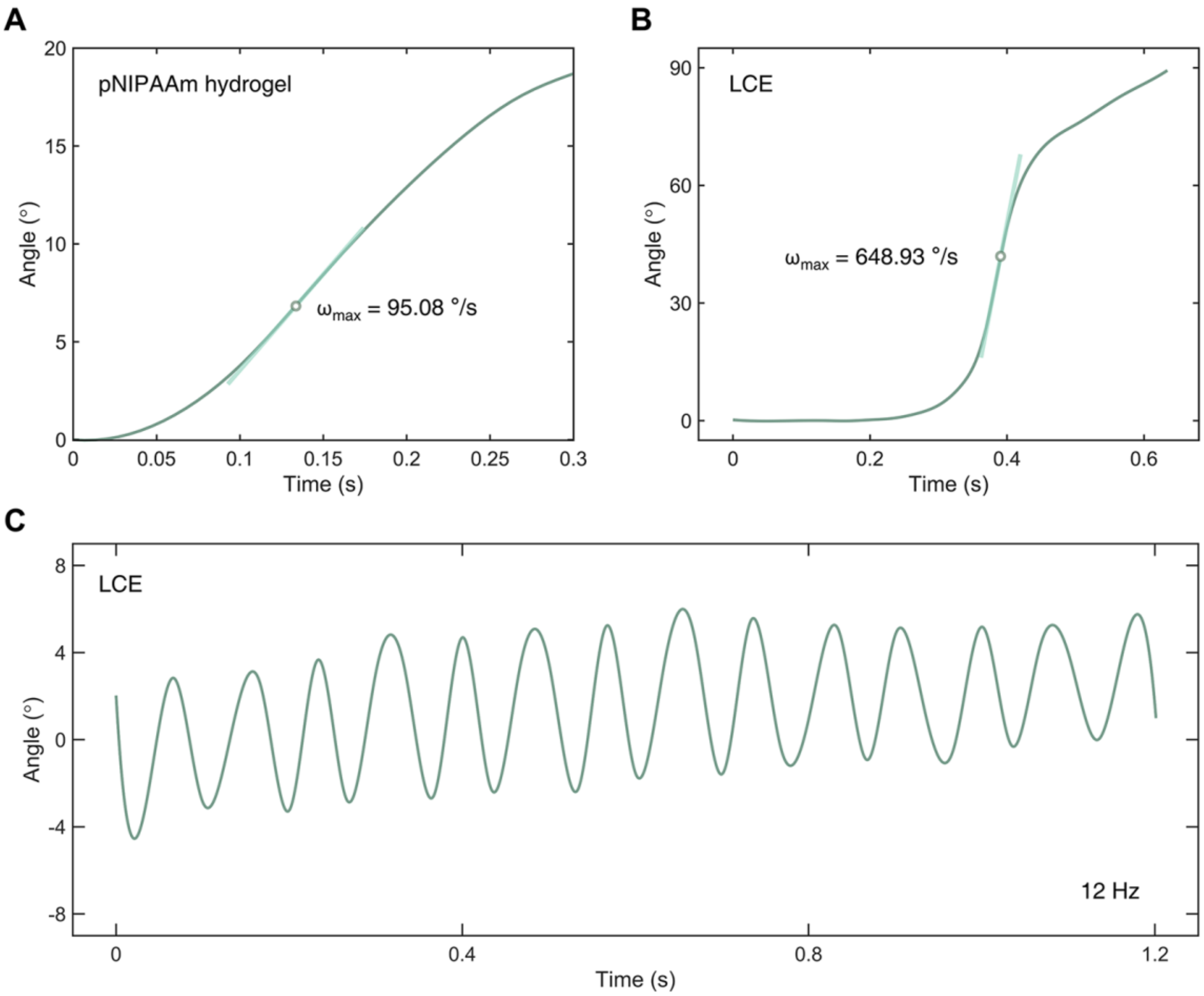

Figure S21. Fast response of PNIPAAm hydrogel and LCE column under the horizontal illumination. (**A**) Fast response of PNIPAAm hydrogel. The maximum response speed is 95.08 °/s when the input power is 485 mW. The PNIPAAm hydrogel sample has a diameter of 0.66 mm and was prepared with ice template method. (**B**) Fast response of LCE column. The maximum response speed is 648.93 °/s when the input power is 84 mW. The diameter of the sample is 0.36 mm and the LCE was prepared with RM257 and RM82 which were equal in mass. (**C**) Oscillation of the LCE column. The oscillation frequency is 12 Hz. The sample was prepared with the same method and the diameter of the sample is 0.37 mm. The fast response performances of the PNIPAAm hydrogel and LCE samples show the potential for the rapid responding devices.

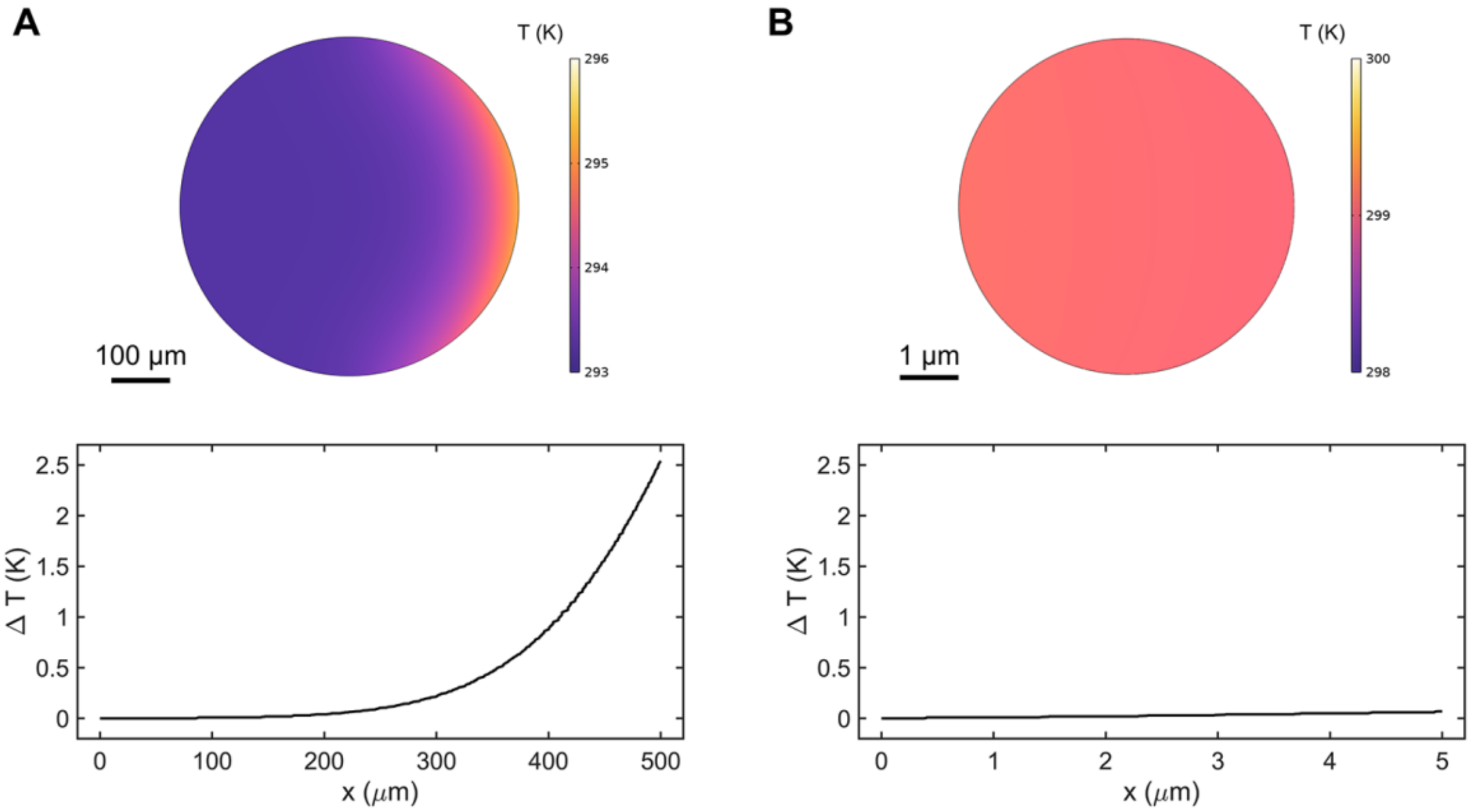

Figure S22. Scaling effect on symmetric breaking of the temperature gradient revealed with simulation for actuators with diameters of 500 μm and 5 μm.

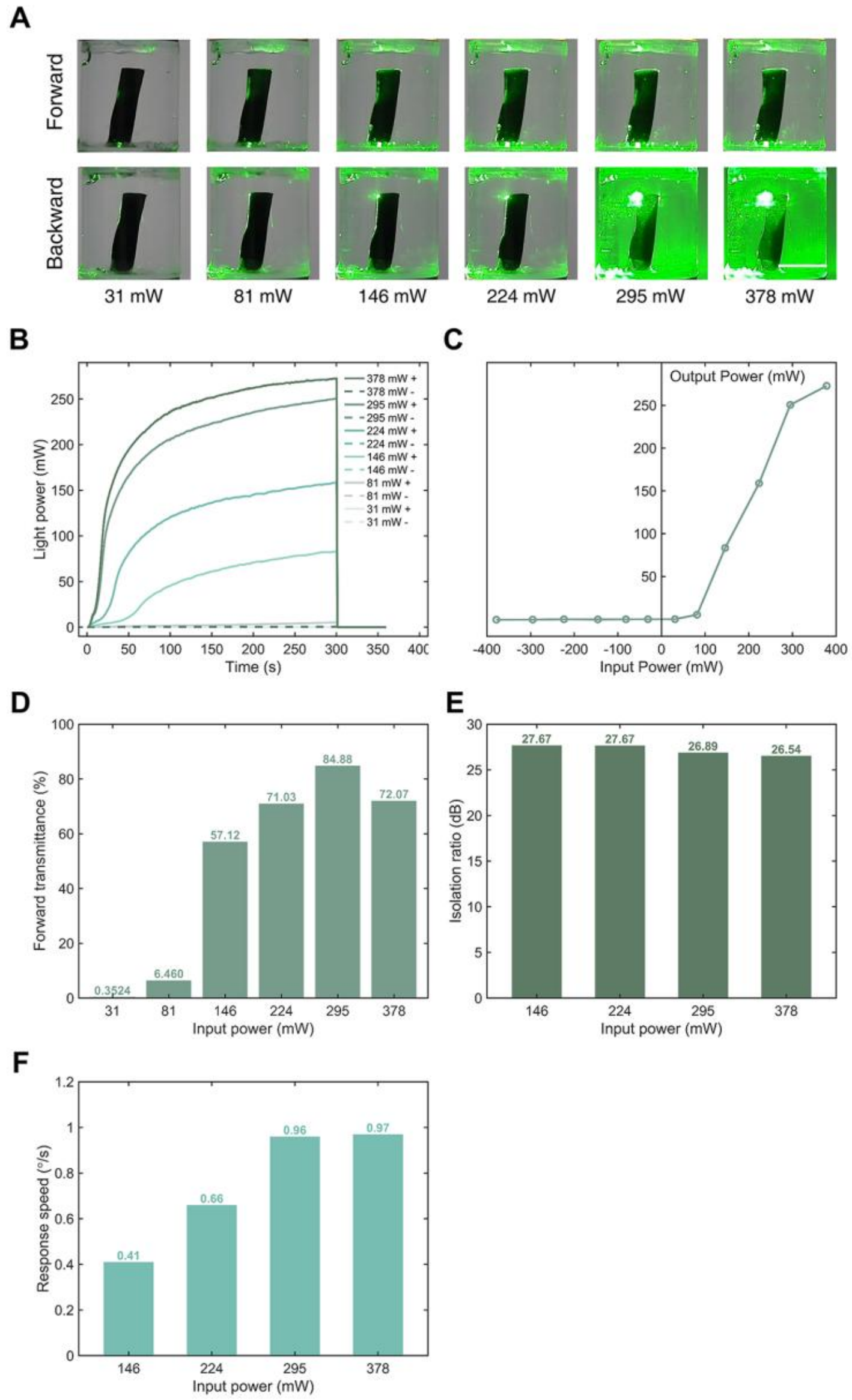

Figure S23. The characteristics of the diode made from room temperature PNIPAAm hydrogel with 2.7 mm inner diameter and 3.8 mm outer diameter. (**A**) Photos of the diode under different illumination power in forward and backward conditions. (**B**) Time evolution of output power for different power of incident light from forward and backward directions. (**C**) Characteristic of the diode was revealed by the curve of output power versus input power. (**D**) Forward transmittance of the diode. (**E**) The isolation ratio of the diode when the diode is in working condition. (When the input power is over 146 mW, the diode enters the operation window, and the isolation ratio is higher than 26 dB.) (**F**) The response speed of the diode when the diode is in working condition. Scale bar, 1 cm.

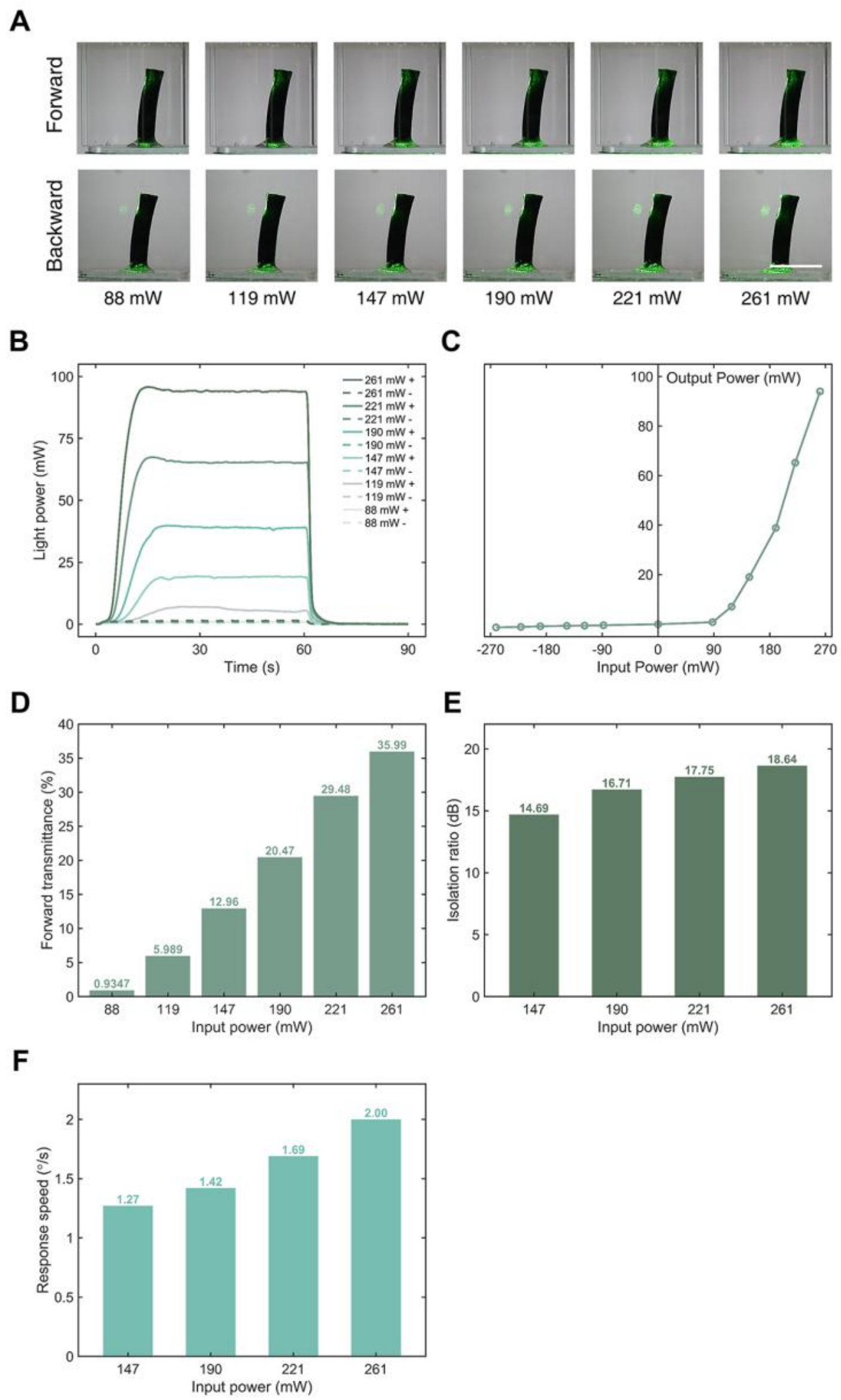

Figure S24. The characteristics of the diode made from PNIPAAm hydrogel prepared with ice template method. (**A**) Photos of the diode under different illumination power in forward and backward conditions. (**B**) Time evolution of output power for different power of incident light from forward and backward directions. (**C**) Characteristic of the diode was revealed by the curve of output power versus input power. (**D**) Forward transmittance of the diode. (**E**) The isolation ratio of the diode when the diode is in working condition. (**F**) The response speed of the diode when the diode is in working condition. (When the input power is higher than 147 mW, the forward transmittance is over 10% and the isolation ratio is over 14 dB. When the input power reaches 261 mW, the forward transmittance can be 35.99% and the isolation ratio is 18.64 dB. The reaction time is shorter comparing with diodes made from LCE or the room temperature prepared PNIPAAm and the output power becomes stable within 20 seconds.) Scale bar, 1 cm.

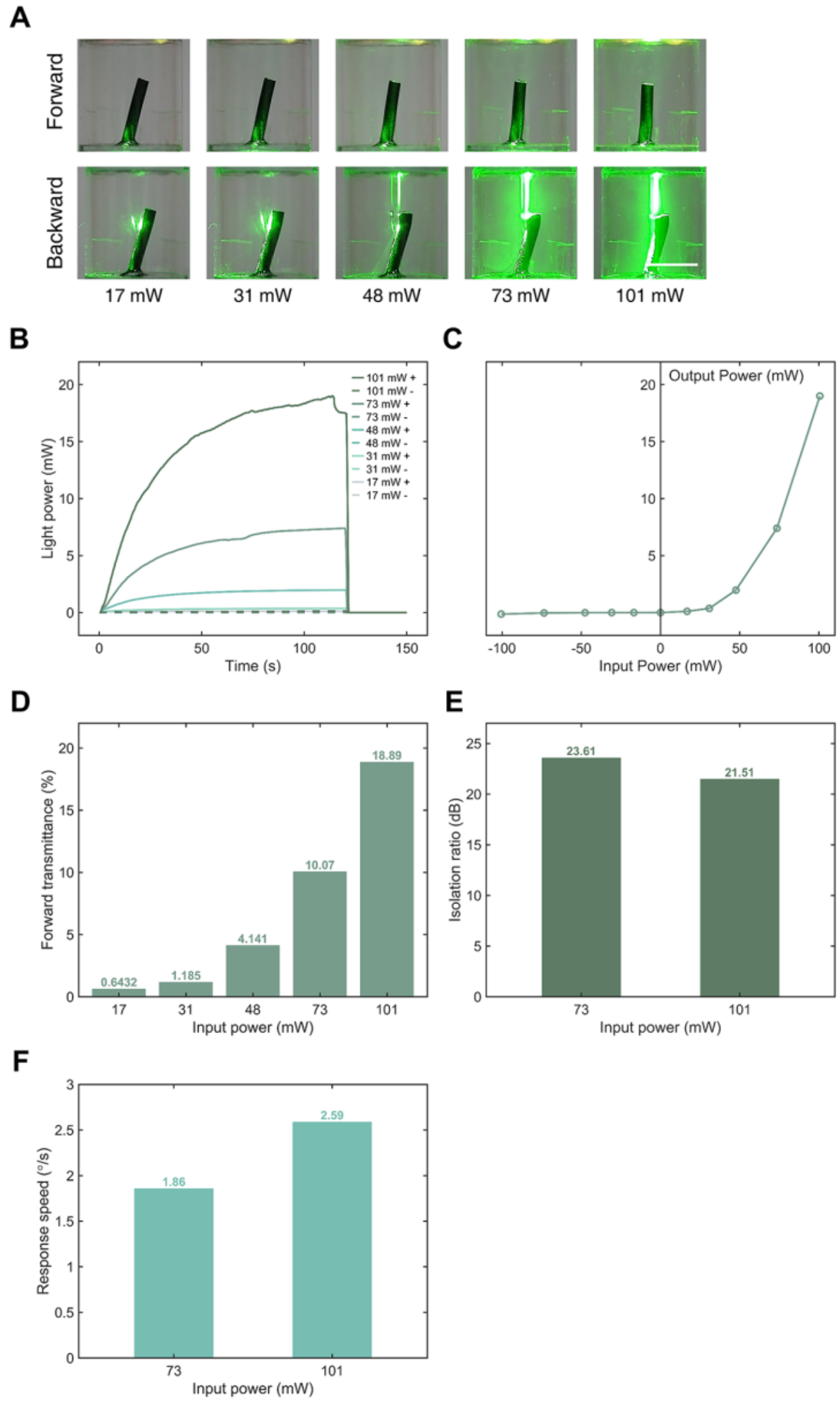

Figure S25. The characteristics of the diode made from RM257 LCE. (**A**) Photos of the diode under different illumination power in forward and backward conditions. (**B**) Time evolution of output power for different power of incident light from forward and backward directions. (**C**) Characteristic of the diode was revealed by the curve of output power versus input power. (**D**) Forward transmittance of the diode. (**E**) The isolation ration of the diode when the diode is in working condition. (When the input power is higher than 186 mW, the forward transmittance is over 10% and the isolation ratio is over 21 dB.) (**F**) The response speed of the diode when the diode is in working condition. Scale bar, 1 cm.

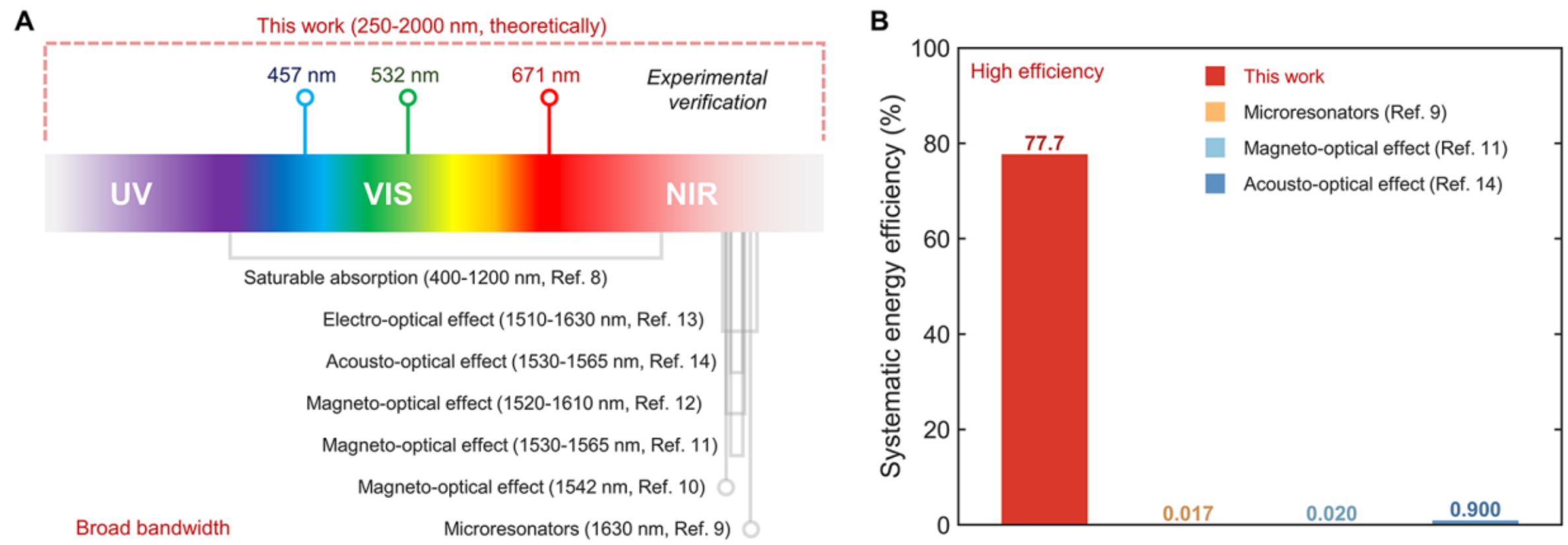

Figure S26. A comparative analysis of all optical diodes reveals the operating bandwidth (A) and efficiency (B) of diodes with different design principles. The work introduced in this article has relatively broad bandwidth (theoretically from 250 nm to 2000 nm) and high energy efficiency.

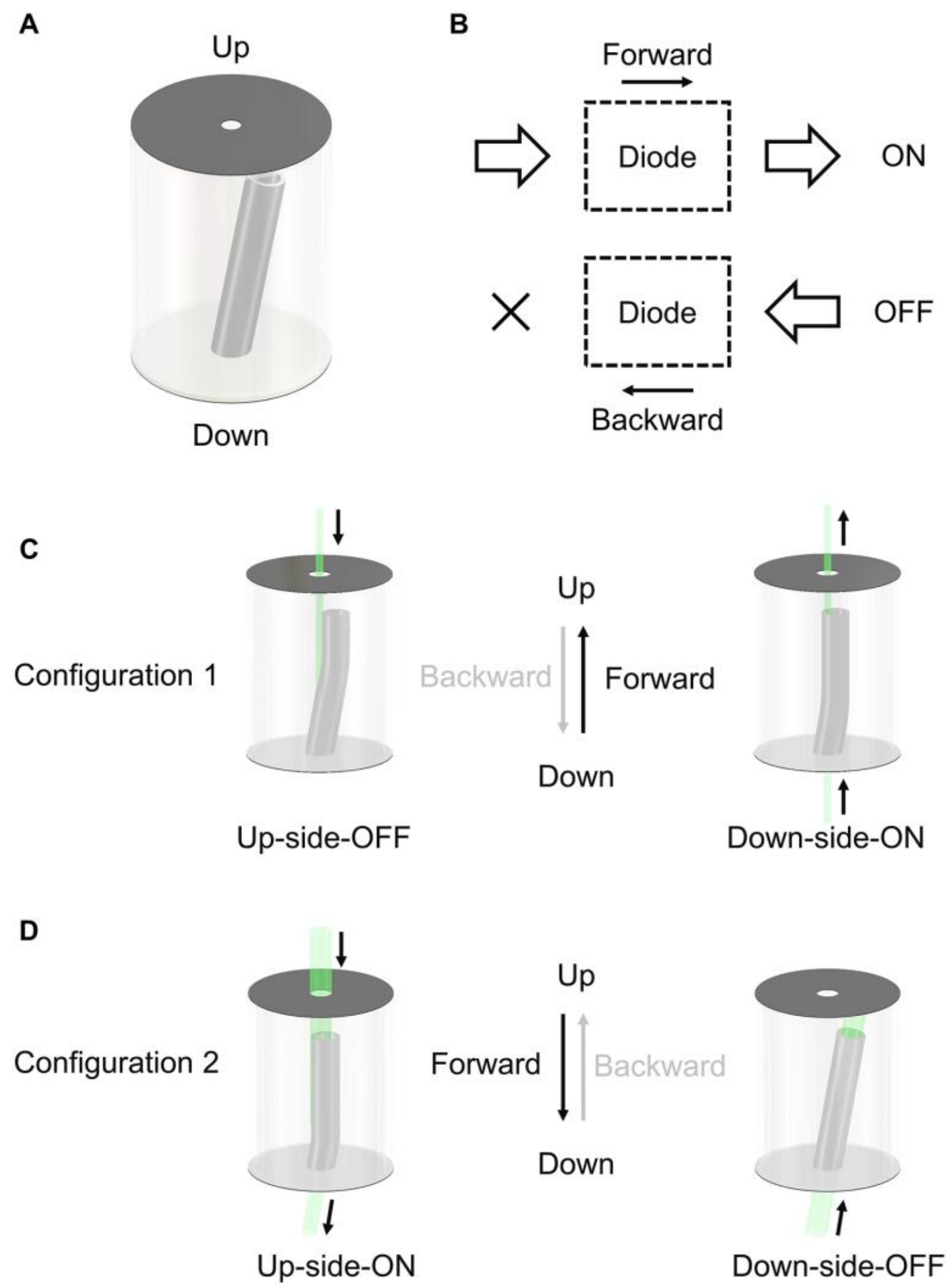

Figure S27. The definition of the description method related to the photothermal responsive waveguide constructed diode and other design principles of the diode. (**A**) The side which nears the free end of the tilted waveguide is defined as the up side of the diode and the side which is stuck with the waveguide is defined as the down side of the diode. (**B**) When the diode works at the conducting state, the diode is on and the transmission direction of the incident light is defined as the forward direction. When the diode works at the blocked state, the diode is off and the transmission direction of the diode is defined as the backward direction. (**C**) Diode configuration 1 which is introduced in Figure 3. The forward direction corresponds to the direction pointing from down to up and the backward direction is reverse. The conducting and blocked state of the diode is recorded as Down-side-ON state and Up-side-OFF state. (**D**) Diode configuration 2. When the wide beam illuminates the tilted waveguide, the structure points the incident direction and parts of the light can be transferred through the guiding layer placed inside. When the input laser comes in the opposite direction through the inner layer and do not illuminate the actuating layer, the output beam is blocked by the shadowing film and there is no light out. The conducting and blocked state of the diode is recorded as Up-side-ON state and Down-side-OFF state.

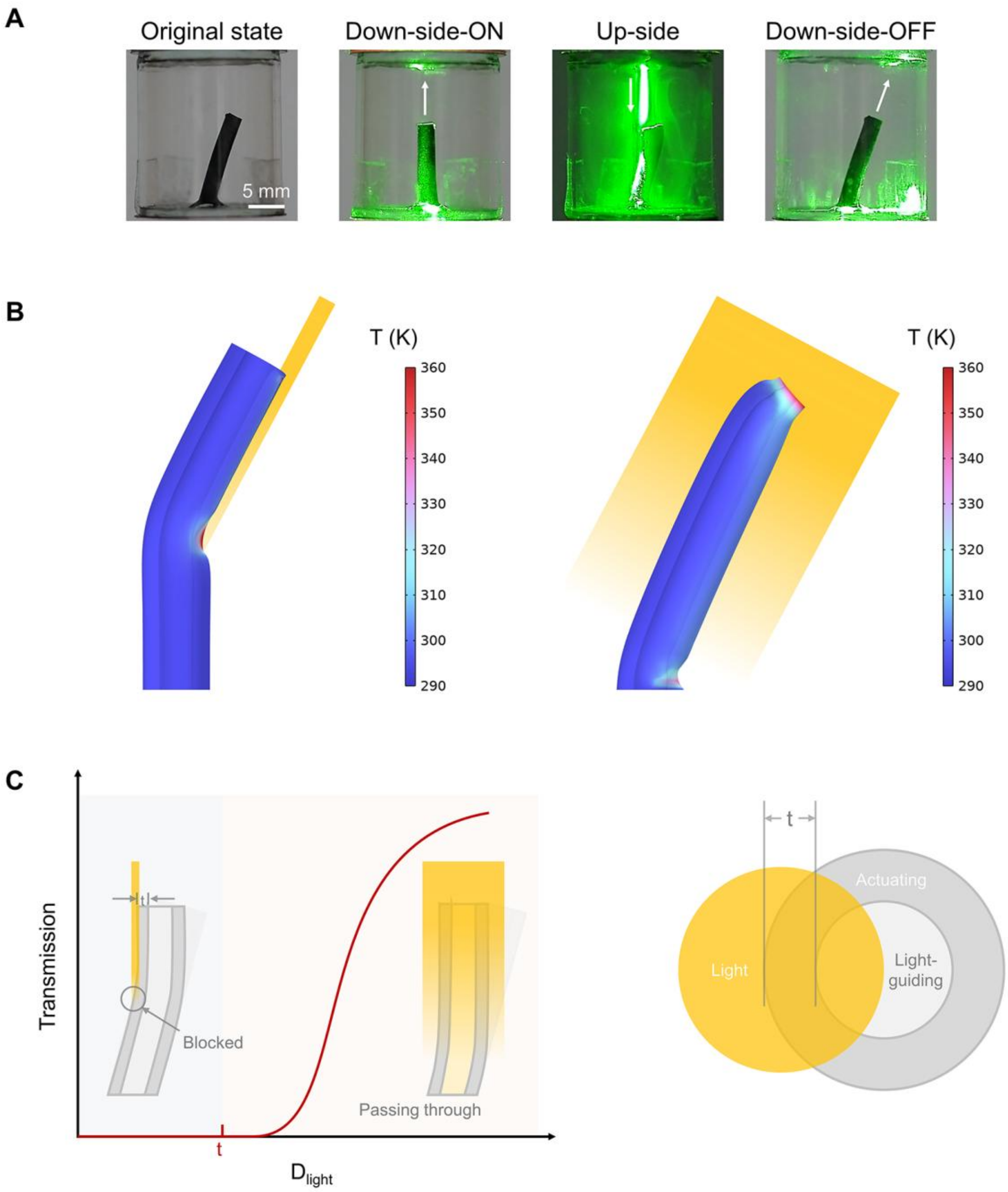

Figure S28. Influence of the light diameter for two configurations of the diode. (**A**) LCE-based diode under three different actuating modes (input power, 129 mW). (**B**) COMSOL simulation results of the diode Up-side mode deformation under two limiting light diameters. (Left, spot light situation (light diameter, 0.75 mm); right, area light situation (light diameter, 20 mm); inside diameter, 1.5 mm; outside diameter, 3 mm.) (**C**) Effect of incident light diameter on Up-side mode output.

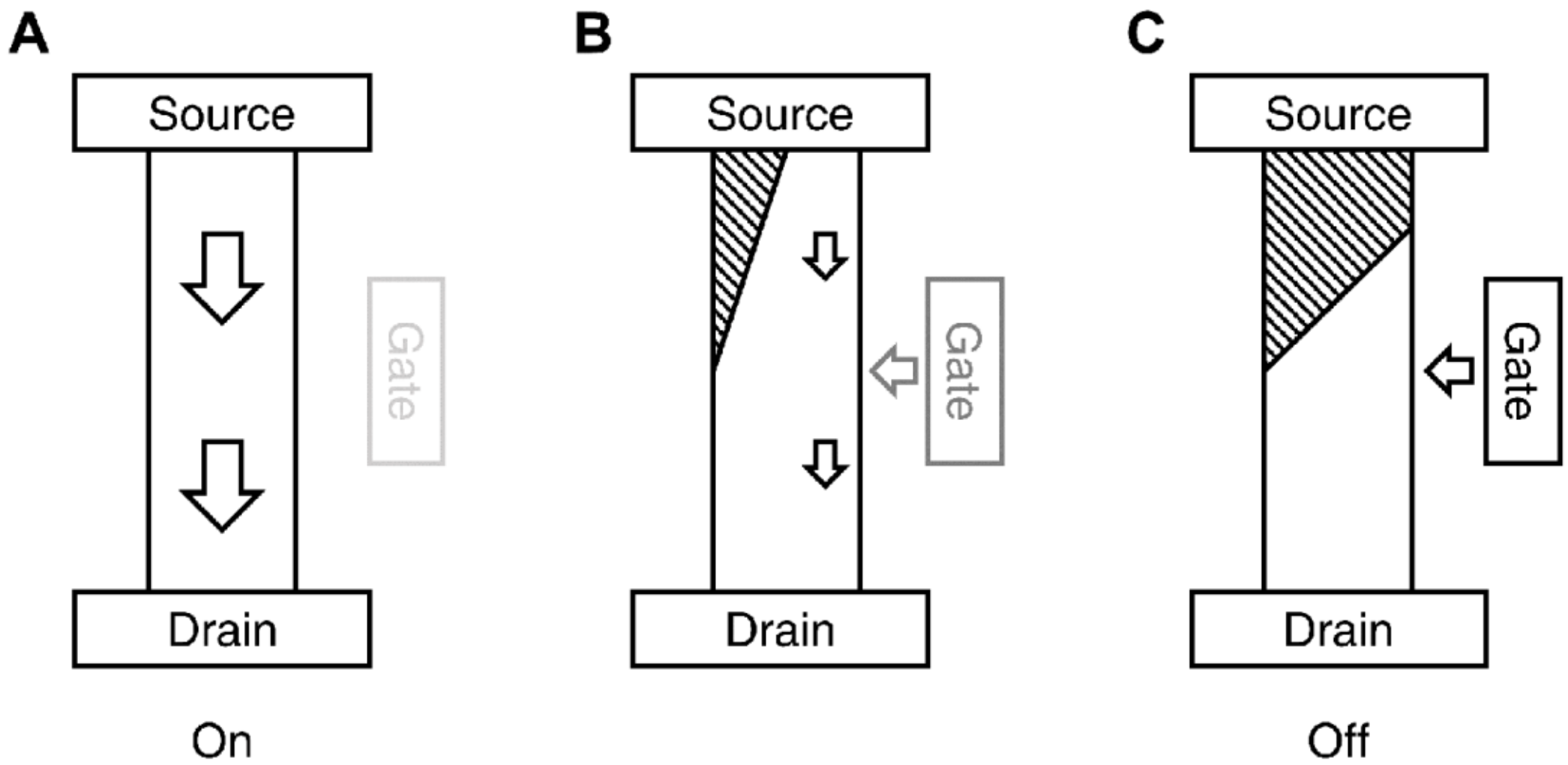

Figure S29. The working schematic diagram of the transistor. (**A**) The photo illumination from the source port can transmit through the device to the drain port when the gate is turned off and the transistor is on. (**B**) When the light from the gate port is turned on, the illumination from the source port is shadowed by the cladding and only part of the source light can reach the drain port. (The shaded area represents the shadowing effect caused by the bending actuator.) (**C**) When the light from the gate port is strong enough, the illumination from the source port cannot reach the drain port for the fully shadow caused by the bending actuator and the transistor is off.

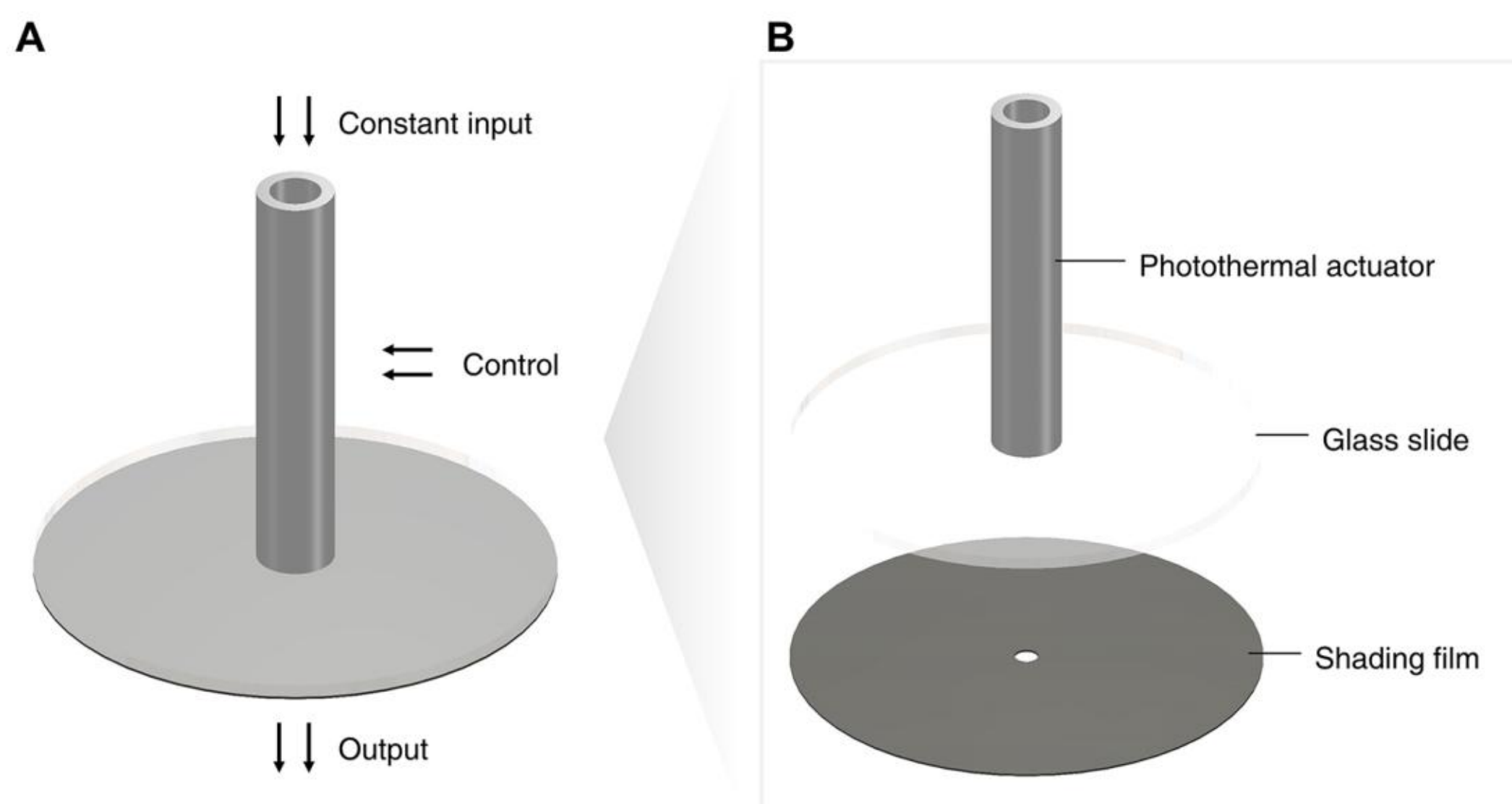

Figure S30. Schematic of the transistor. (**A**) The structure of the transistor together with the settings of the constant input laser and control laser. (**B**) The assembly and packaging methods of the transistor.

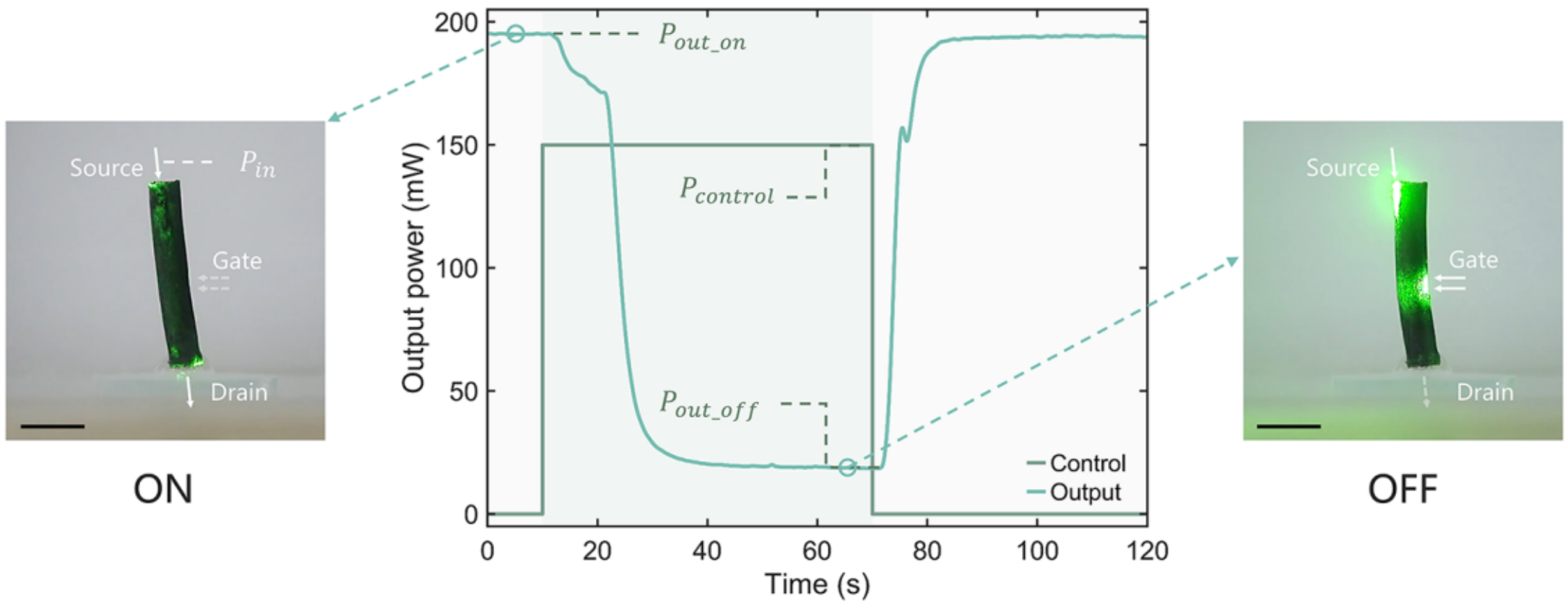

Figure S31. Time variation of the transistor corresponding to Figures 4B-D. (The control power was 150 mW and the input power was 233 mW. The photos on the side reveal the conducting state and cut-off state of the transistor. When the control light was turned off, the transistor was in the conducting state and the stable output power was calculated and defined as P_{out_on}. When the control light was turned on, the transistor was in the cut-off state and the stable output power was calculated and defined as P_{out_off}. The power of the control light was defined as $P_{control}$.) Scale bars, 1 cm.

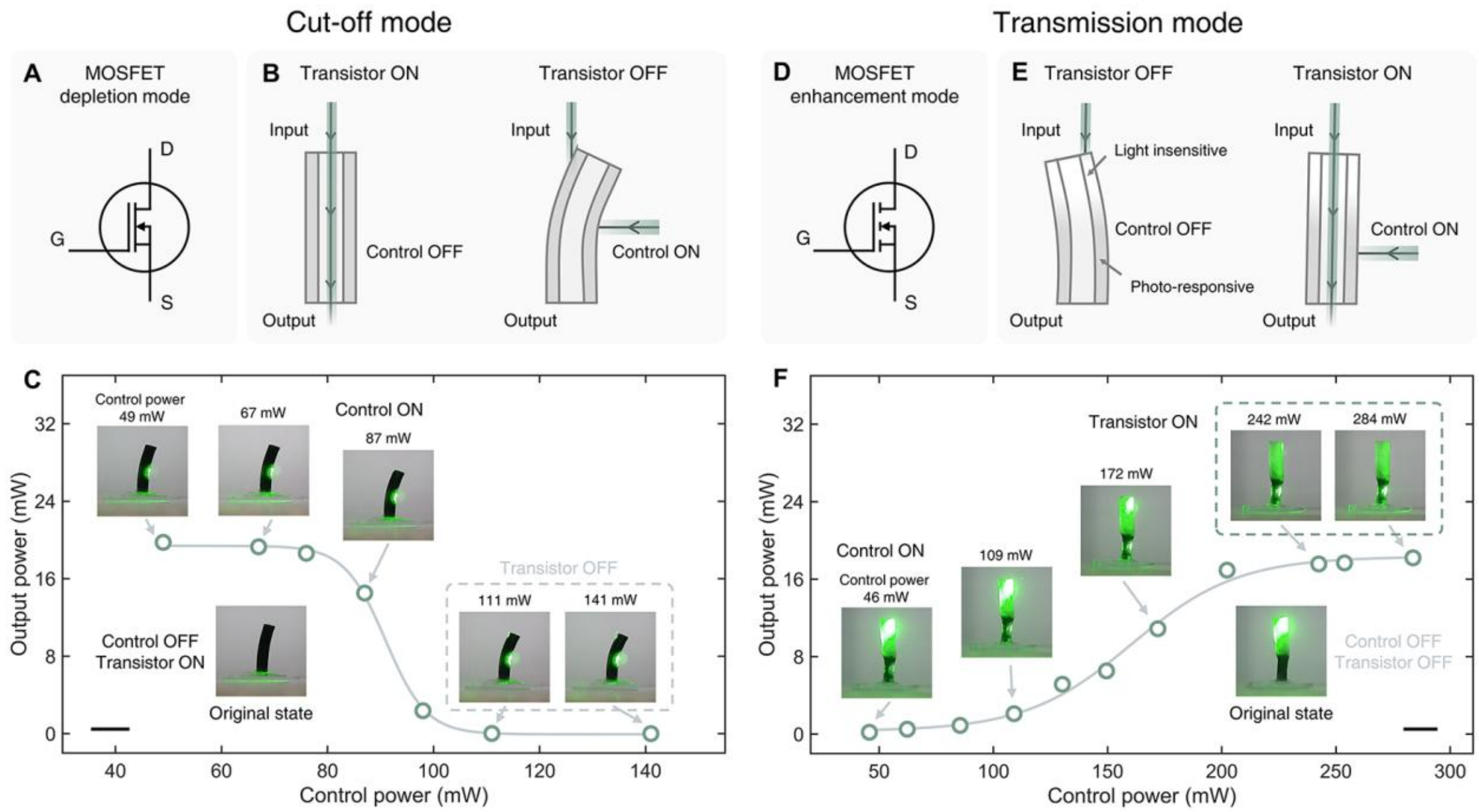

Figure S32. Comparison of cut-off mode transistor and transmission mode transistor. (**A**) Symbol of the depletion mode MOSFET, which the cut-off mode transistor has same characteristics as. (**B**) Design principle of the cut-off mode transistor. The original state of the transistor is a straight responsive waveguide, with the photo-responsive cladding. When the control signal is off, the structure maintains the original form. The input pass through the waveguide and the transistor is on. When the control signal is on, the structure bends toward the incident direction of the control signal. The input light is sheltered by the cladding and the transistor is off. (**C**) The output power and the experimental images of the cut-off mode transistor under different control power. The output power decreases when the control power increases. (The cladding of the transistor was built with ice templated method prepared PNIPAAm hydrogel. The input power was 22 mW.) (**D**) Symbol of the enhancement mode MOSFET, which the transmission mode transistor has same characteristics as. (**E**) Design principle of the transmission mode transistor. The original state of the transistor is a curved responsive waveguide. The top of the cladding is light insensitive, while the other region of the cladding is photo-responsive. The input light is parallel with the axis of the bottom side and is sheltered by the top side of the cladding when the control signal is off. When the control signal is on, the structure bends slightly towards the direction of the incident control light and the input laser passes through the device from the inner layer of the waveguide. (**F**) The output power and the experimental images of the transmission mode transistor under different control power. The output power increases when the control power increases. (The cladding of the transistor was built with ice templated method prepared hydrogel with the light insensitive PAM upper part and the photo responsive PNIPAAm lower part. The input power was 22 mW.) Scale bars, 1 cm.

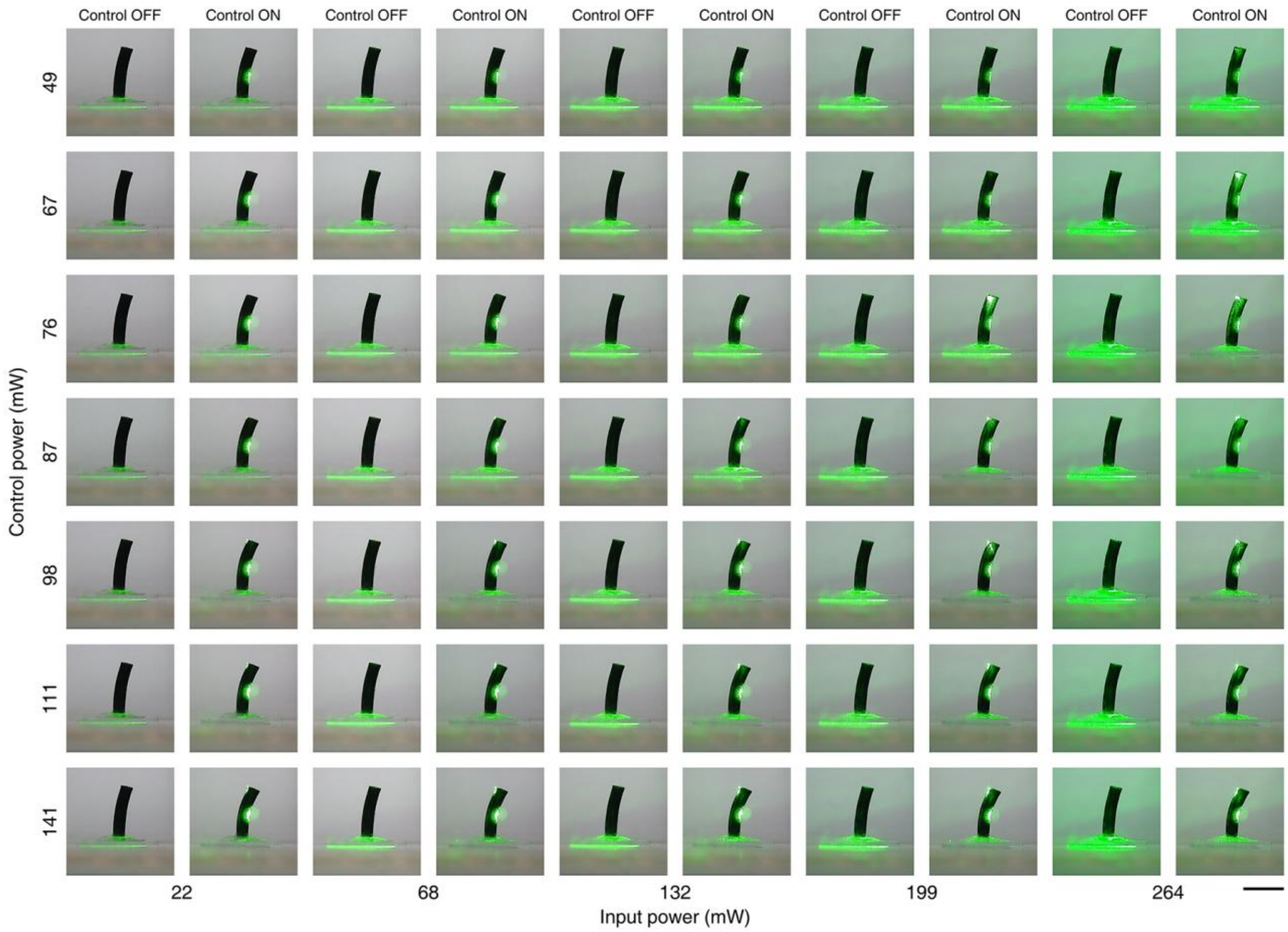

Figure S33. Experimental images of the transistor behavior under different input and control light. The input power was 22, 68, 132, 199 and 264 mW. The control power was 49, 67, 76, 87, 98, 111 and 141 mW. With the same input, the bending deformation of the responsive waveguide increases when the control power increases. With the same control, the scattering light enhances as the input power increases. (The results correspond to Figures 4E-H, and the cladding of the transistor was ice templated method prepared PNIPAAm hydrogel.) Scale bars, 1 cm.

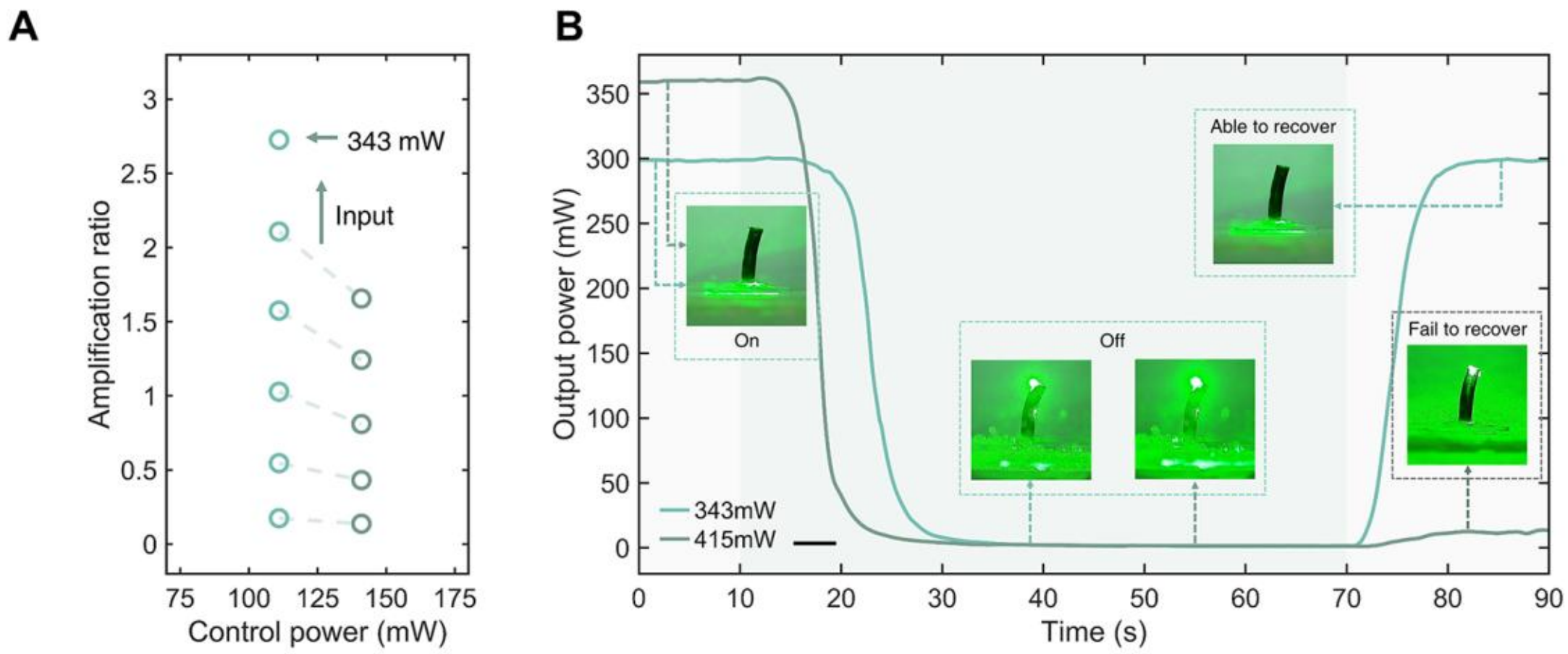

Figure S34. The amplification characteristic of the transistor made with the hollow PNIPAAm hydrogel. (**A**) The amplification ratio of the transistor. When the input power was 343 mW, the amplification ratio was 2.73. (**B**) Time evolution of output power under input power of 343 mW and 415 mW. For both conditions, the control power was 111 mW. When the control light turned on, the responsive waveguide bent toward the incident direction. The input light was blocked by the cladding and the output power was reduced close to 0. When the control light turned on, the transistor tended to recover to the original state. The structure fully recovered under 343 mW input, but when the input power reached 415 mW, the structure failed to recover and the transistor failed. The input threshold of this transistor is below 415 mW. Scale bar, 1 cm.

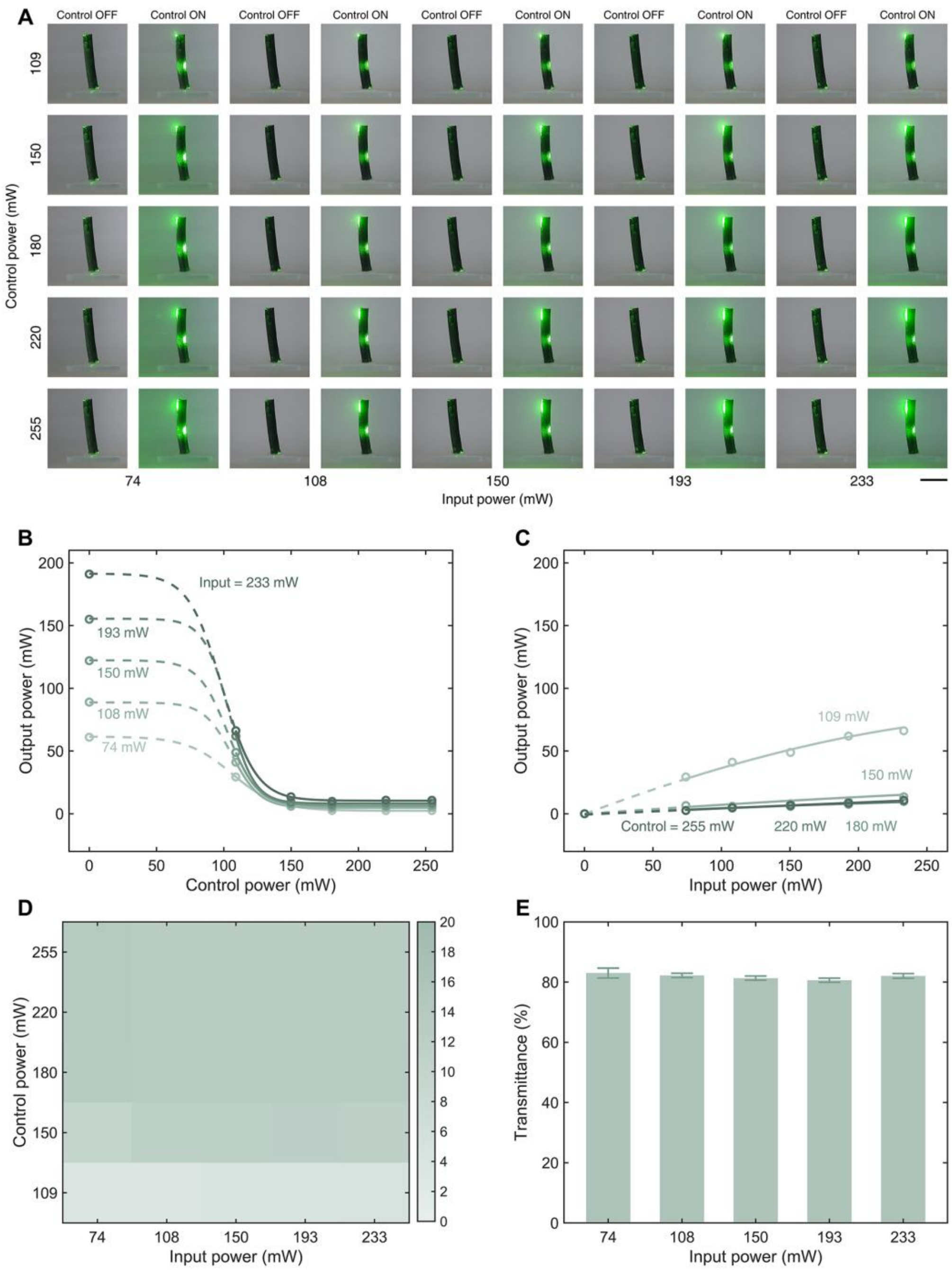

Figure S35. The characteristics of the double layer transistor, with ice template method prepared PNIPAAm hydrogel cladding and light insensitive transparent PAM hydrogel core. (**A**) Experimental images of the transistor behavior under different input and control light. Scale bar, 1 cm. (**B**) Transfer characteristics of the transistor, revealing the correlation between output and control power. (**C**) Output characteristics of the transistor, revealing the correlation between output and input power. (**D**) The isolation ratio of the transistor. (**E**) The transmittance of the transistor in the conducting state.

A

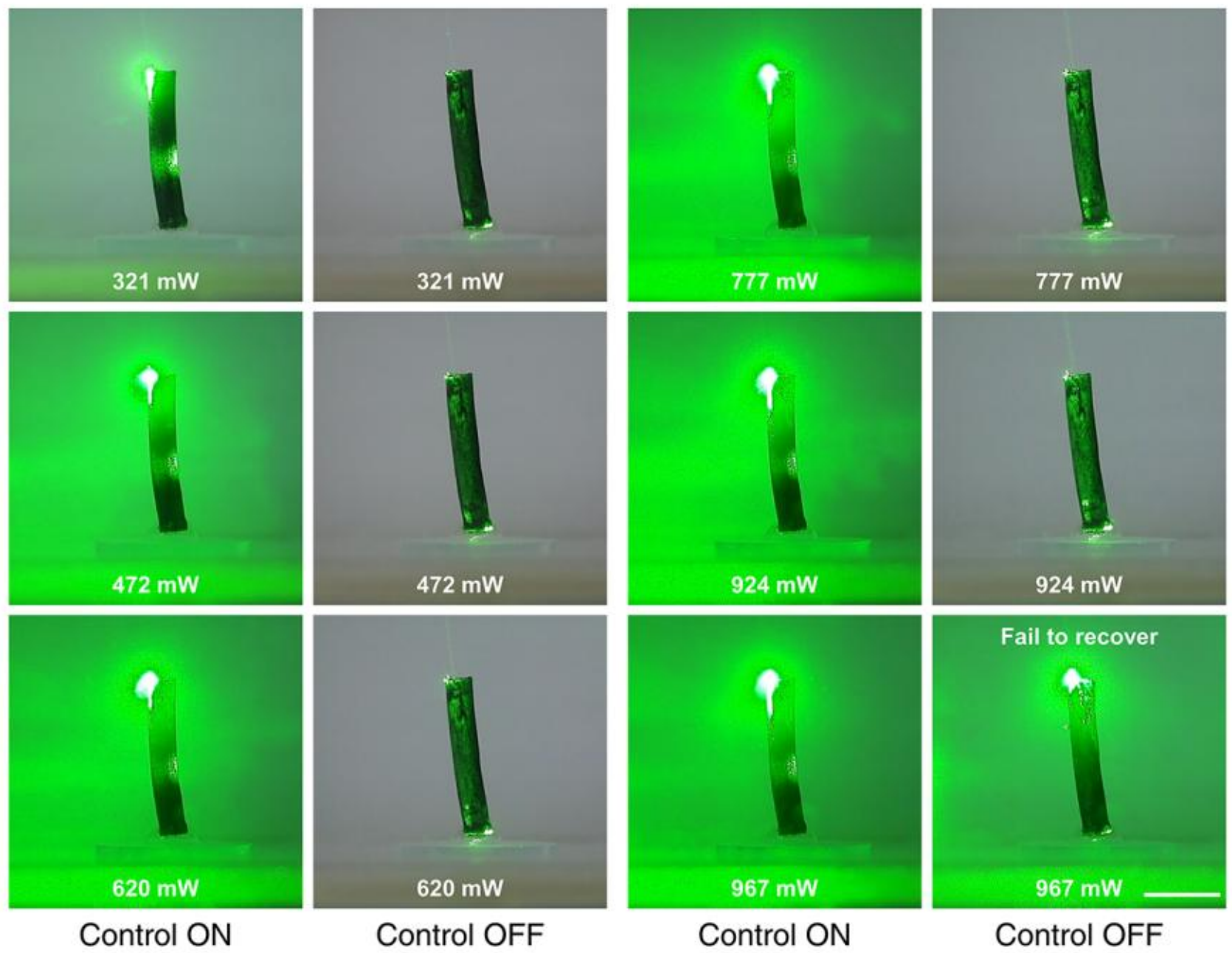

B

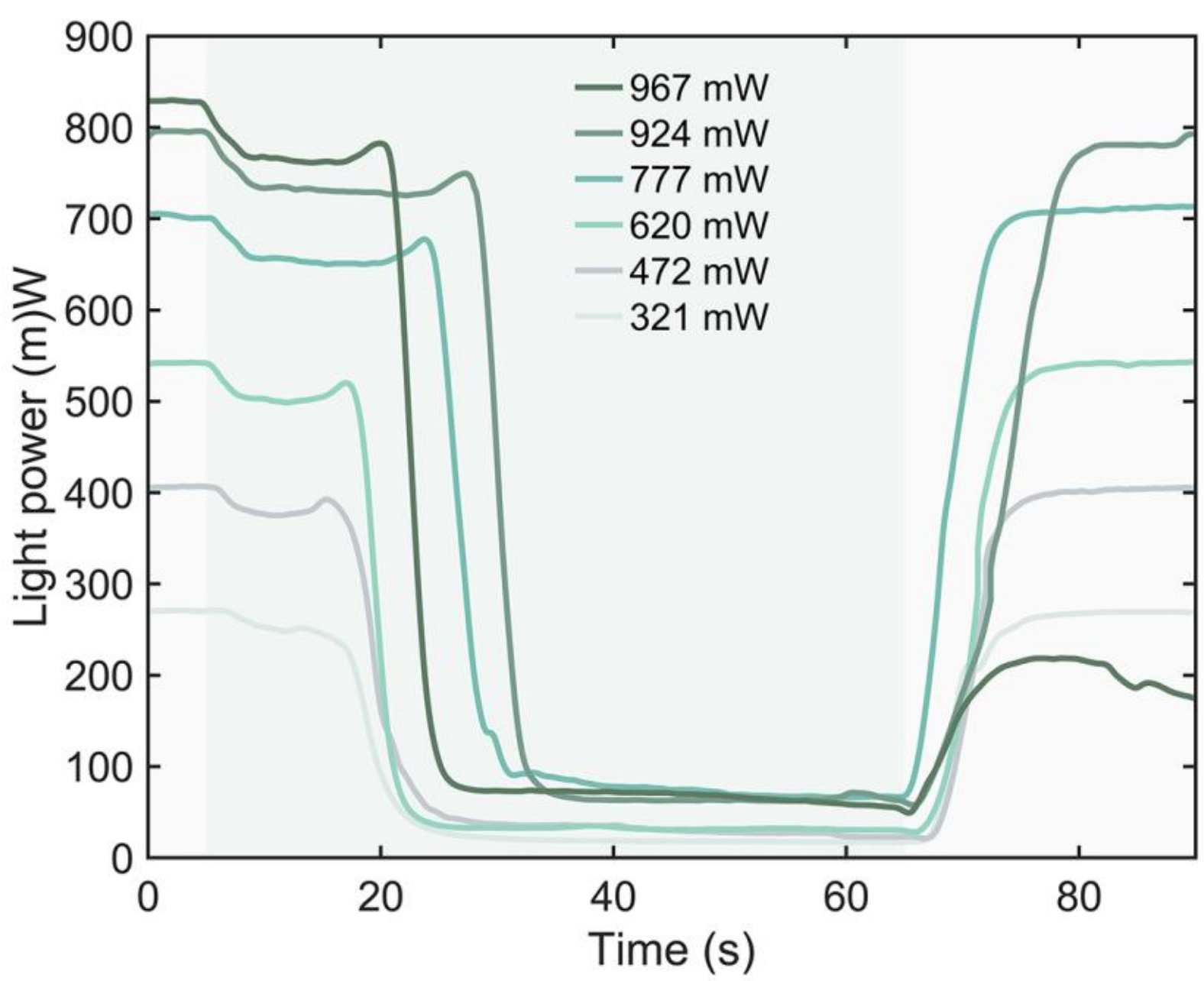

Figure S36. Amplification behavior and the maximum input power threshold of the double-layer transistor. (**A**) Photos of the transistor with the increasing input power when the control light was turned on then turned off. The control power was 108 mW and the input power increased gradually. The transistor could fully recover when the control light turned off under the input power within 924 mW. Under 967 mW input, the transistor failed to recover and the device failed. Scale bar, 1 cm. (**B**) Time variation of output power with increasing input power.

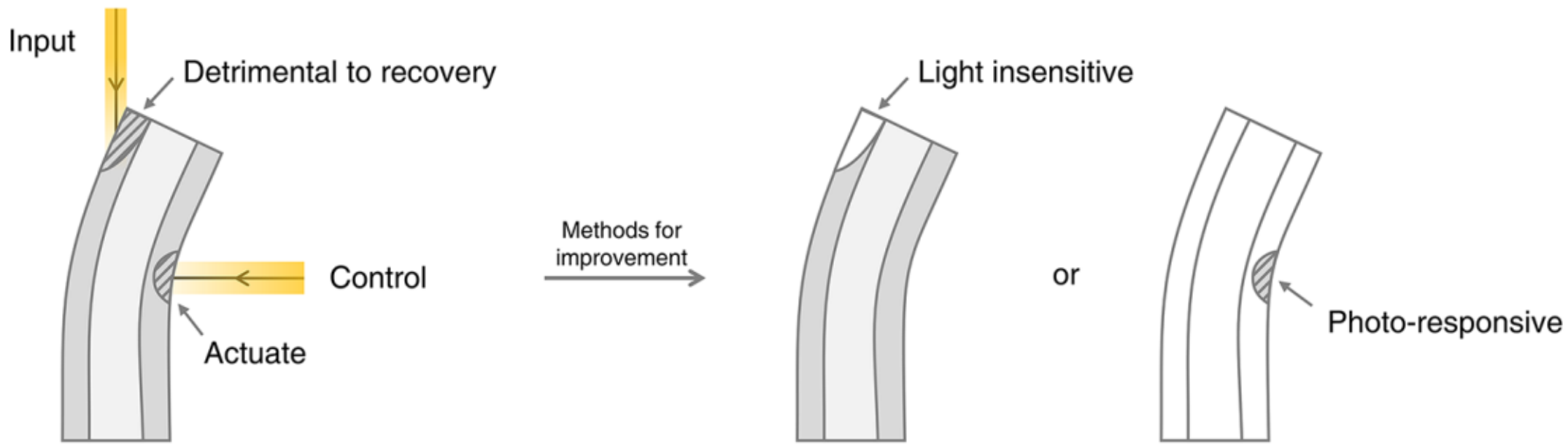

Figure S37. Influences of input and control signal on transistor responses and methods to expand the operation window of the transistor. When the transistor is in operation, the illuminated region affects the behavior of the device. The control laser illuminated area actuates the responsive waveguide to cut off the input laser, while the input light illuminated area prevents the waveguide to recover when the control laser if off, which limits the maximum input power of the transistor. Heterogeneous cladding can improve the limited input, with the light insensitive part on the top side or the only photo-responsive part at the control illuminated area. For the optimized structure, the input power can increase infinitely theoretically, under the condition that the insensitive material is not penetrated by input laser.

Logic gate	Symbol	Structure
NOT	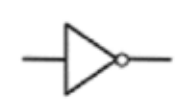	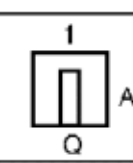
AND	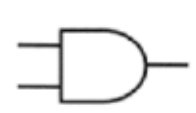	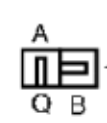
OR	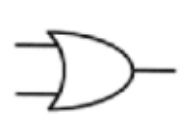	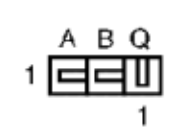
NAND	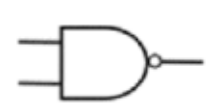	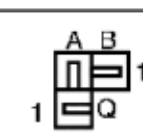
NOR	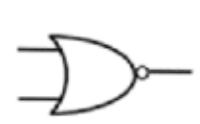	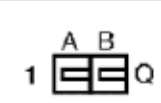

Input: A, B; Output: Q

Figure S38. The conclusion of the design principle of different logic gates with the basic elements. NOT gate is built with one basic element. AND gate and NOR gate are built with two basic elements. OR gate and NAND gate are built with three basic elements. The letters on the side of the elements represent the input or output content of the ports. “1” represents that the port is normally open. “A” and “B” represent the input and “Q” represent the output.

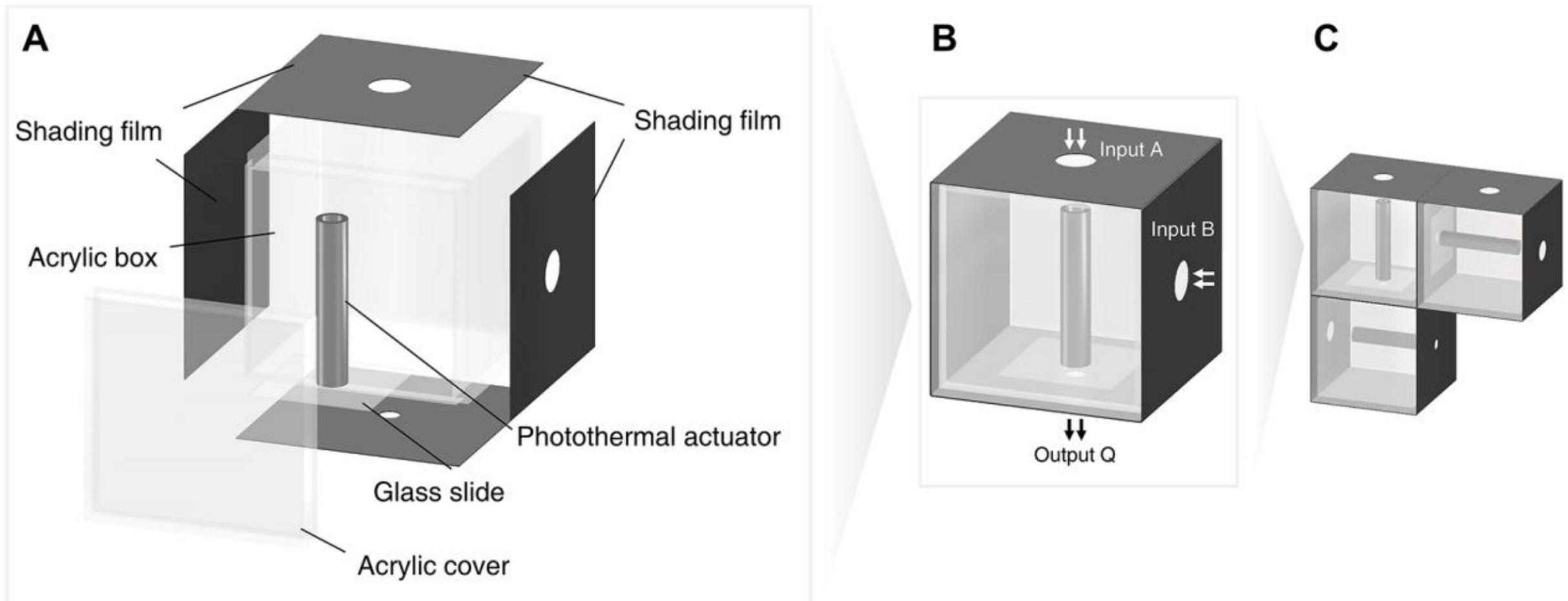

Figure S39. Schematic of the logic gate. (**A**) The assembly and packaging methods of the logic gate element. (**B**) The structure and the port definition of the basic element. (**C**) The structure of NAND gate with three assembled basic elements.

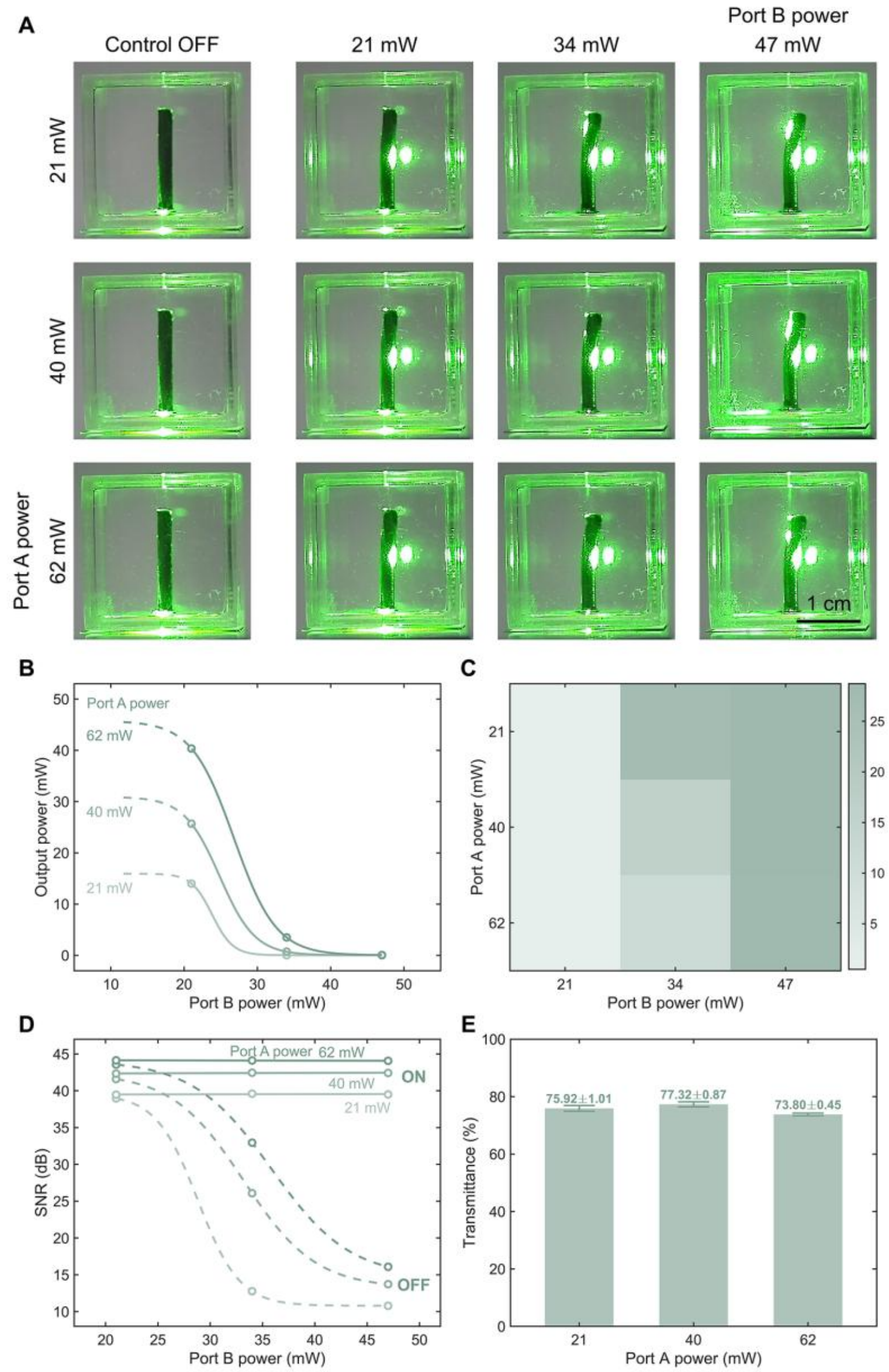

Figure S40. Characteristic of the LCE-based logic unit. (**A**) Experimental images of the logic unit under different input power (port A power: 21 mW, 40 mW, 62 mW; port B power: 21 mW, 34 mW, 47 mW). (**B**) Output characteristics of the logic unit, revealing the correlation between output and port B power which controls the deformation of the actuator. (**C**) Isolation ratio of the logic unit. (**D**) Signal-to-noise ratio of the logic unit under different input power combinations. (**E**) Transmittance of the logic unit in the conducting state.

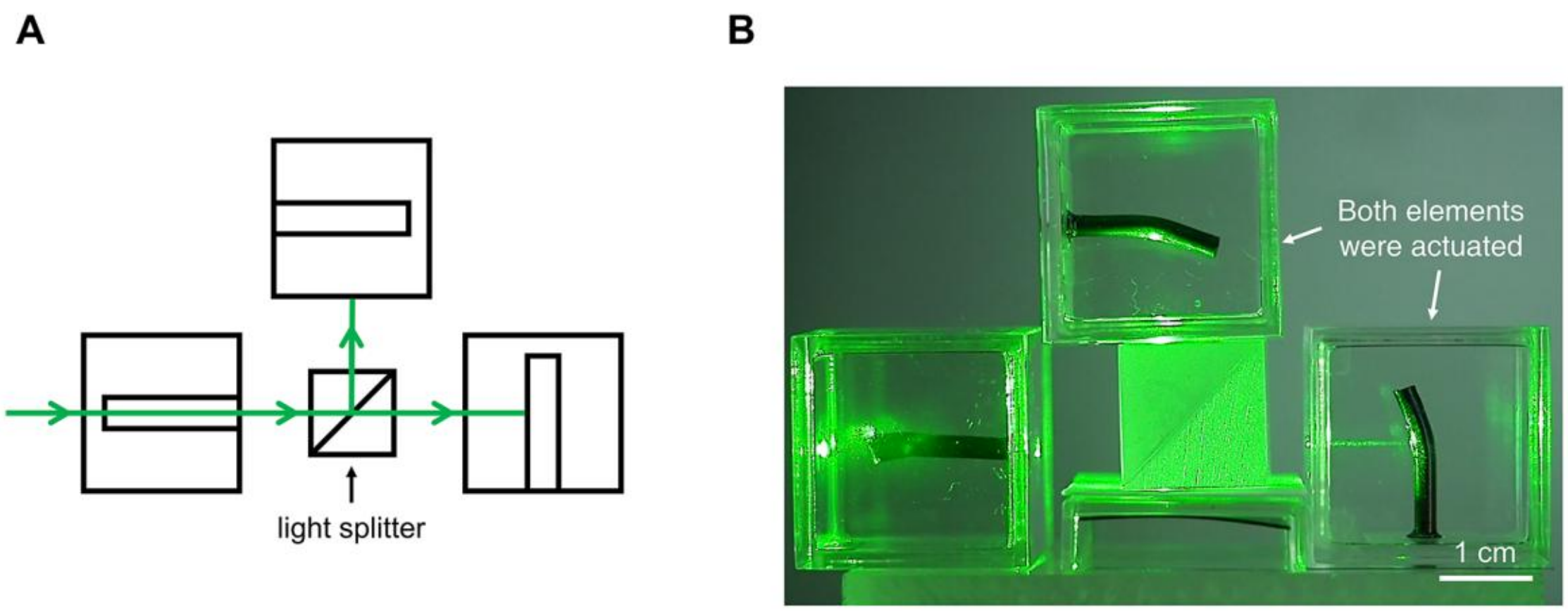

Figure S41. Fan-out validation with the LCE-based logic unit. (**A**) Schematic of the fan-out validation with the input light passing through one logic unit then split into two beams actuating two successor units. (**B**) Experiment image reveals the output light can actuate two successor units (input power: 333 mW).

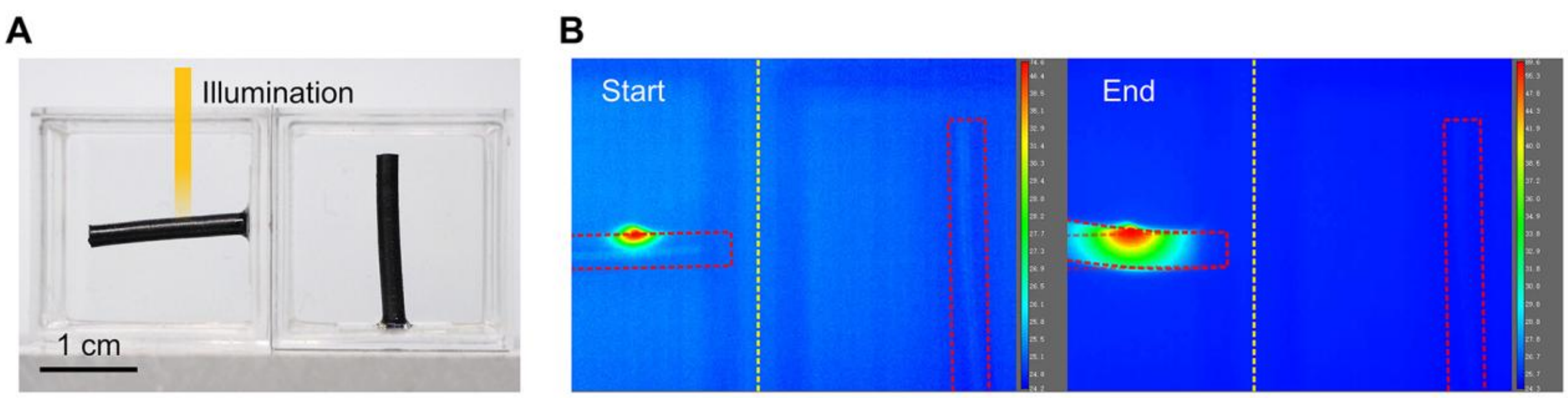

Figure S42. Thermal crosstalk effects propagating from the activated logic unit to the neighboring component (light power: 40 mW).

A

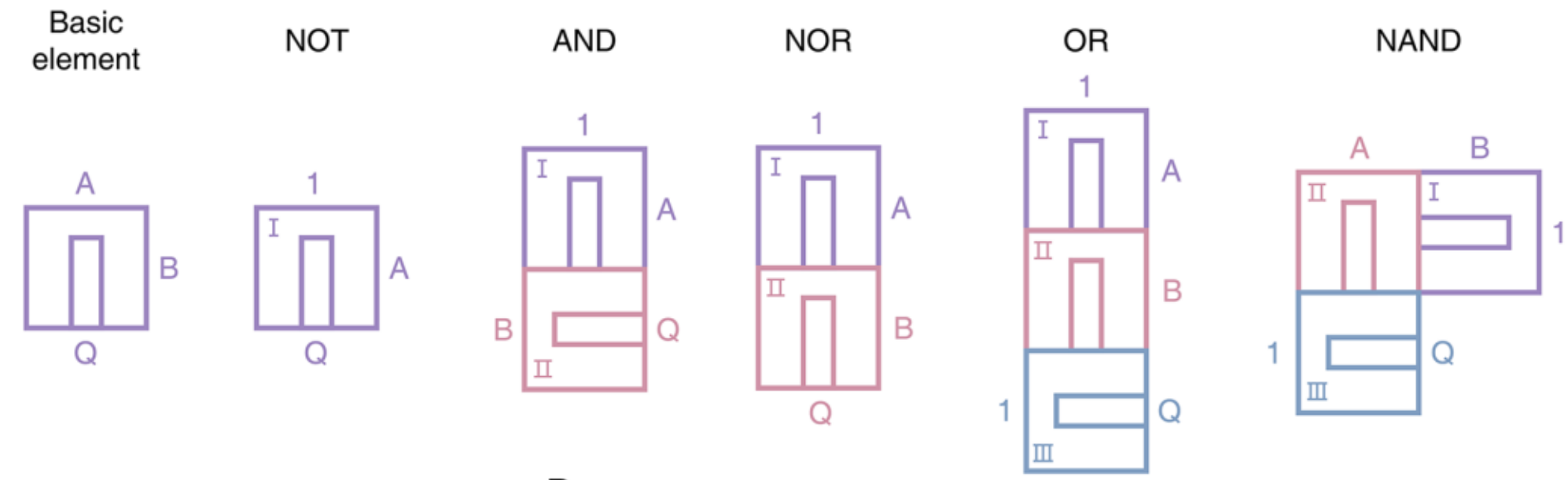

B

Input		Output
A	B	Q
1	1	0
1	0	1
0	1	0
0	0	0

C

number of the unit

NOT — I — $Q = Q_{I} = \neg A$

$A_{I} = 1$	$B_{I} = A$	$Q_{I} = \neg A$
1	1	0
1	0	1

D

AND

I			II $Q = Q_{II} = A \wedge B$		
$A_{I} = 1$	$B_{I} = B$	Q_{I}	$A_{II} = A$	$B_{II} = Q_{I}$	$Q_{II} = A \wedge B$
1	1	0	1	0	1
1	0	1	1	1	0
1	1	0	0	0	0
1	0	1	0	1	0

E

NOR

I			II $Q = Q_{II} = A \downarrow B$		
$A_{I} = 1$	$B_{I} = A$	Q_{I}	$A_{II} = Q_{I}$	$B_{II} = B$	$Q_{II} = A \downarrow B$
1	1	0	0	1	0
1	1	0	0	0	0
1	0	1	1	1	0
1	0	1	1	0	1

F

OR

I			II			III $Q = Q_{III} = A \vee B$		
$A_{I} = 1$	$B_{I} = A$	Q_{I}	$A_{II} = Q_{I}$	$B_{II} = B$	Q_{II}	$A_{III} = 1$	$B_{III} = Q_{II}$	$Q_{III} = A \vee B$
1	1	0	0	1	0	1	0	1
1	1	0	0	0	0	1	0	1
1	0	1	1	1	0	1	0	1
1	0	1	1	0	1	1	1	0

G

NAND

I			II			III $Q = Q_{III} = A \uparrow B$		
$A_{I} = 1$	$B_{I} = B$	Q_{I}	$A_{II} = A$	$B_{II} = Q_{I}$	Q_{II}	$A_{III} = 1$	$B_{III} = Q_{II}$	$Q_{III} = A \uparrow B$
1	1	0	1	0	1	1	1	0
1	0	1	1	1	0	1	0	1
1	1	0	0	0	0	1	0	1
1	0	1	0	1	0	1	0	1

Figure S43. The design principles of the logic gates with the structure and the truth table. (**A**) The combination modes of the basic element, NOT gate, AND gate, NOR gate, OR gate and NAND gate. (**B** to **G**), The truth table of the basic element and the logic gates. The cells with grey shadings represent the consistent input. The cells with green shadings represent the ports which connect two elements and the input of the successor element is determined by the output of the predecessor element.

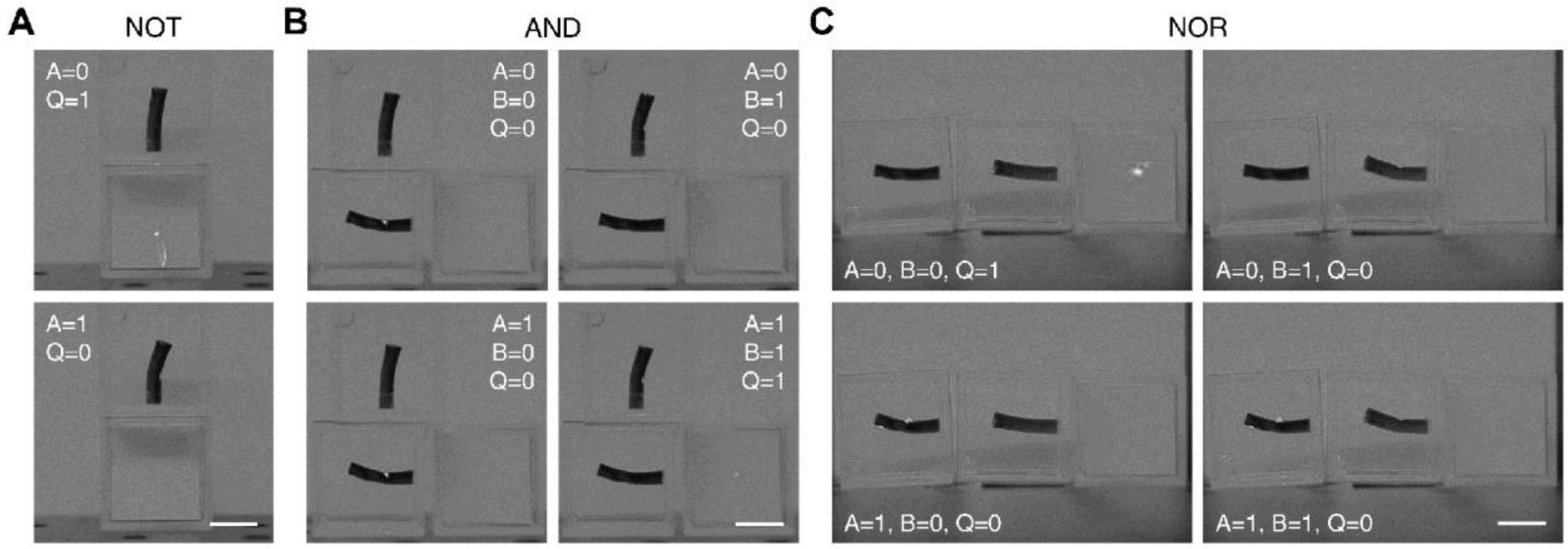

Figure S44. The experimental verification of NOT gate, AND gate and NOR gate with the PNIPAAm hydrogel cladding actuator. (The actuators were prepared with ice template methods.) Scale bars, 1 cm.

A

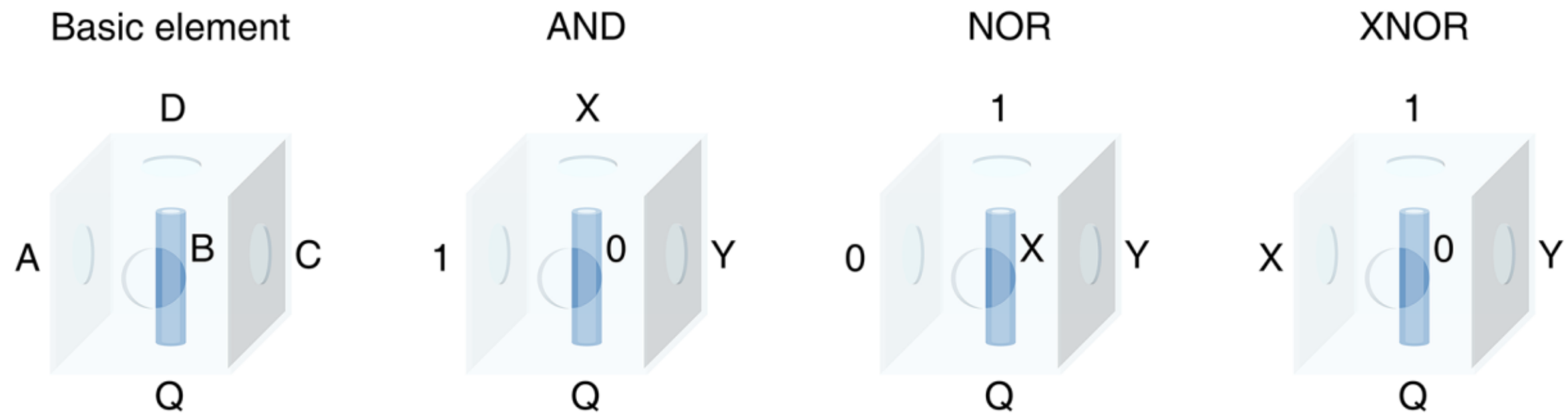

B

Input / Output

A	B	C	D	Q
1	1	1	1	0
1	1	1	0	0
1	1	0	1	0
1	1	0	0	0
1	0	1	1	1
1	0	1	0	0
1	0	0	1	0
1	0	0	0	0
0	1	1	1	0
0	1	1	0	0
0	1	0	1	0
0	1	0	0	0
0	0	1	1	0
0	0	1	0	0
0	0	0	1	1
0	0	0	0	0

AND

A = 1	B = 0	C = X	D = Y	Q = X∧Y
1	0	1	1	1
1	0	1	0	0
1	0	0	1	0
1	0	0	0	0

NOR

A = 0	B = X	C = Y	D = 1	Q = X↓Y
0	1	1	1	0
0	1	0	1	0
0	0	1	1	0
0	0	0	1	1

XNOR

A = X	B = 0	C = Y	D = 1	Q = X⊙Y
1	0	1	1	1
1	0	0	1	0
0	0	1	1	0
0	0	0	1	1

Figure S45. Schematic of five ports logic module and the design principle of different logic gates. (**A**) The design principle of the AND, NOR and XNOR gates based on the five ports element. (**B**) The truth table of the basic element together with the AND, NOR and XNOR gates. (With different combination of the definition of the input ports, AND, NOR and XNOR gates can be realized with only one basic element. When the NOT gate design is consistent with the design principle mentioned in Figure 5, NAND, OR and XOR gates can be realized with the combination of two basic elements, by adding the existing single element AND, NOR and XNOR gate, with the NOT gate.)

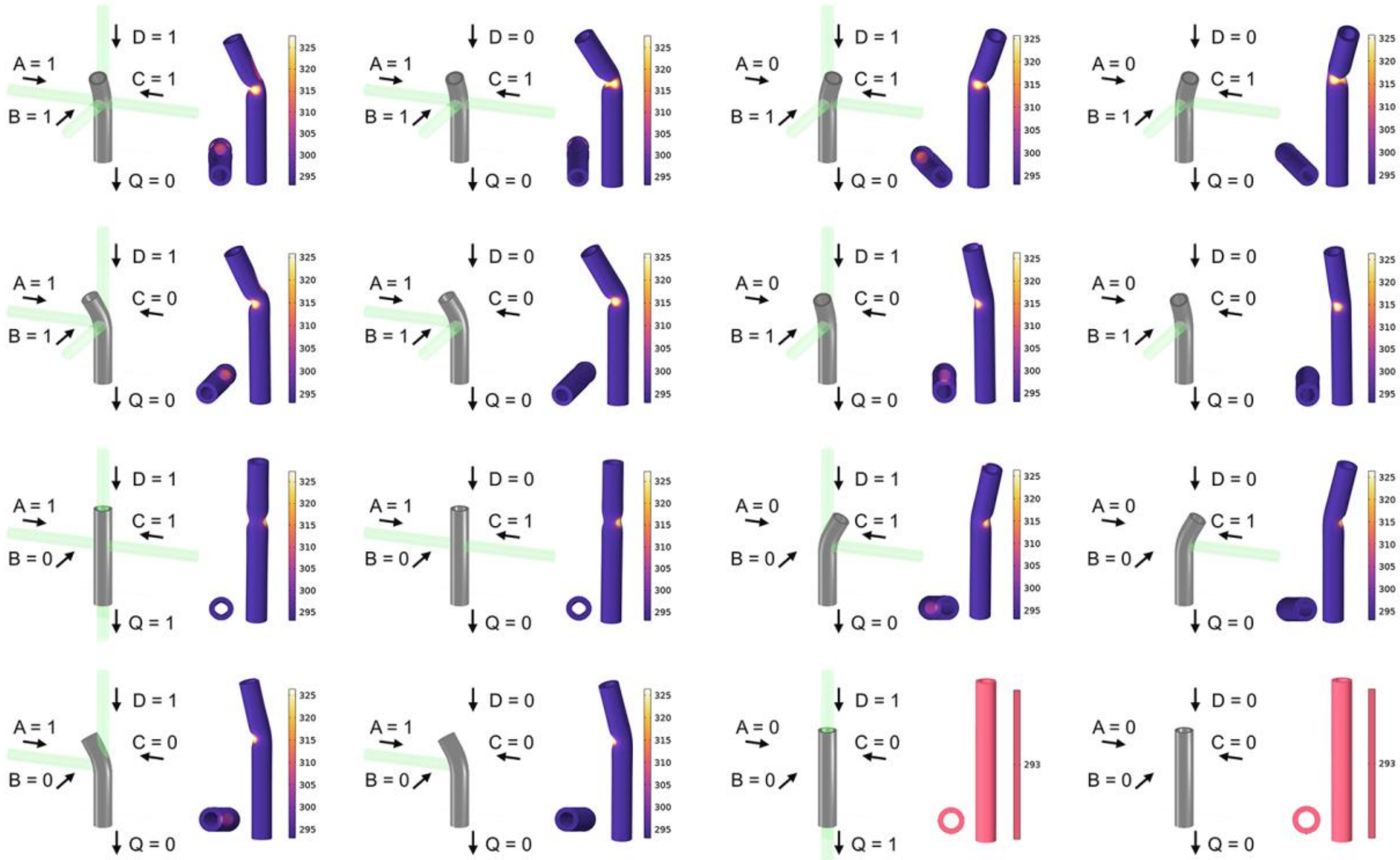

Figure S46. Simulations of five ports logic module under different combinations of inputs, including the schematic diagrams and the temperature distributions of 16 types of input combinations. The temperature distributions involve the upper view and overall view together with the deformation results. When the input port D is on and from the upper view there is no shelter from top to the bottom, the device is considered to be conducting. Based on the design assumption and the simulation results, the basic element is conducting when the input is A, C and D on, B off, or A, B and C off, D on.

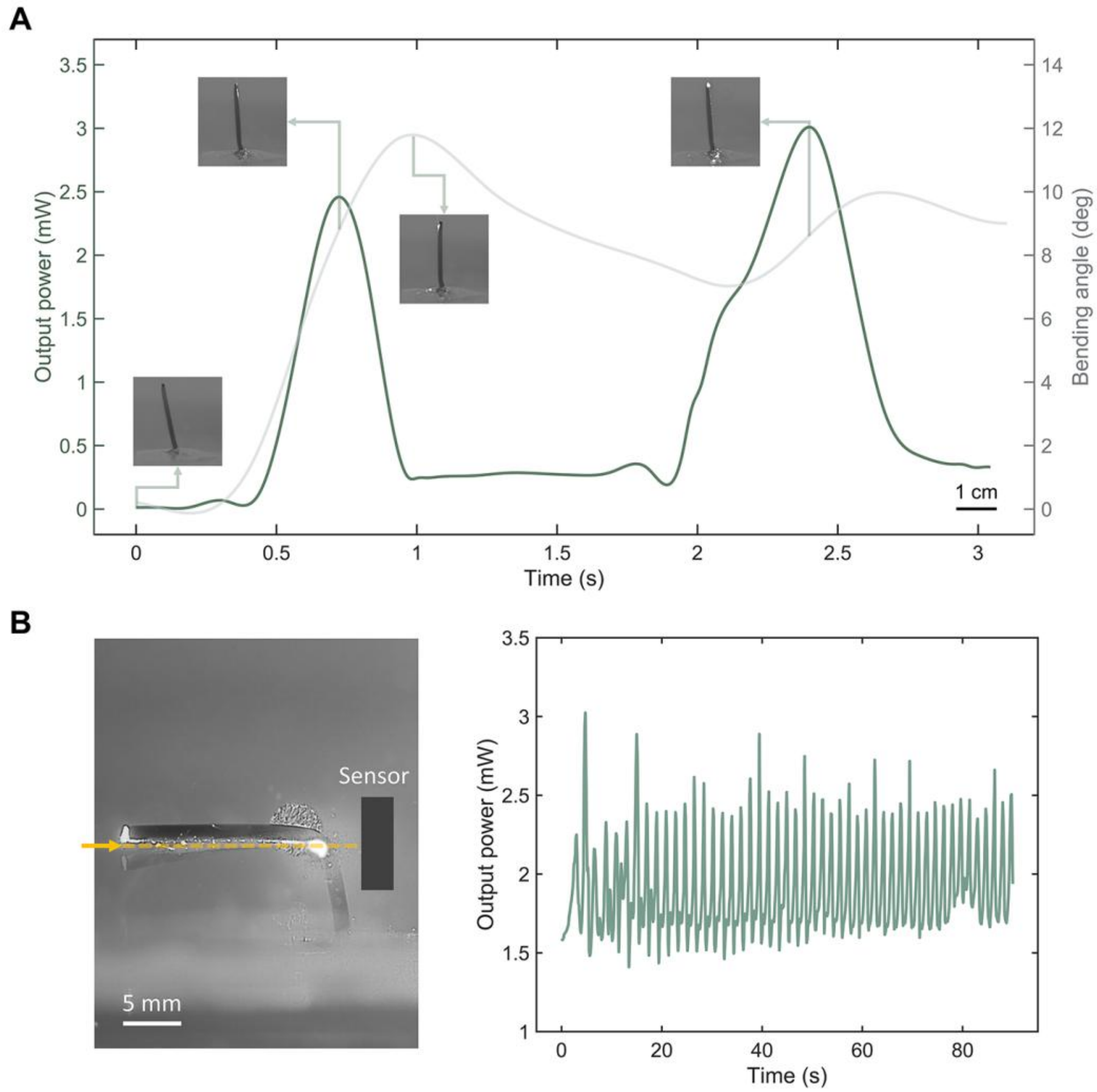

Figure S47. The periodic output of the oscillatory actuator. (A) The output of the light passing through a hollow actuator (input power 530 mW). (B) The output generated from the shadowing and exposing effect of the oscillatory behavior (input power, 418 mW). (Both actuators were fabricated with room temperature prepared PNIPAAm hydrogel.)

Supplemental Tables

Table S1. Characteristics of all optical diodes based on photothermal responsive materials when the isolation ratio is at the maximum value.

Num	Material	Isolation ratio (dB)	Forward transmittance (%)	Wavelength (nm)	Power (mW)
1	PNIPAAm hydrogel	34.93	57.3	532	340
2	PNIPAAm hydrogel	28.01	23.4	457	153
3	PNIPAAm hydrogel	30.38	24.4	532	153
4	PNIPAAm hydrogel	31.61	21.7	671	153
5	PNIPAAm hydrogel (larger diameter)	27.67	71.03	532	224
6	PNIPAAm hydrogel (ice templet)	18.64	35.99	532	261
7	RM257 LCE	23.61	10.07	532	73

Table S2. Characteristics of reported optical diodes.

	Principle	Bandwidth*	Isolation ratio	Insertion loss	Systematic loss	Response time	Power
8	Saturable absorption	800 nm	3 dB	/	/	167.2 ps	> 1GW/cm^2
9	Micro-resonators	0.04 nm	28.4 dB	21.4 dB	37.6 dB	2 μs	25.1 mW
10	Magneto-optical effect	1.32 nm	19.5 dB	18.8 dB	/	/	/
11	Magneto-optical effect	35 nm	21 dB	8 dB	37 dB	/	/
12	Magneto-optical effect	90 nm	27 dB	2.9 dB	/	/	6.31 mW
13	Electro-optical effect	120 nm	48 dB	0.5 dB	/	/	126 mW
14	Acousto-optical effect	35 nm	38 dB	1 dB	20.45 dB	/	104 mW
This work	Photothermal responsive materials	~1750 nm**	34.99 dB	/	1.10 dB	60 s	340 mW

*Bandwidth summarized in the table are the diode operating ranges given in the literature and do not correspond to the optimal isolation ratio and efficiency listed in the table.

**Theoretical bandwidth for PNIPAAm-hydrogel based diode calculated with the absorbance results (Fig. S2).

Table S3. Parameters used in the simulation for deformation under outside and inside illumination.

Parameters	Descriptions	Values
D_{out}	outer diameter of cladding	$3\ mm$
D_{in}	inner diameter of cladding	$2\ mm$
H	height of cladding	$16\ mm$
E_{gel}	Young's modulus of dry gel	$100\ MPa$
υ_{gel}	Poisson's ratio of dry gel	0.5
ν	molecule volume of water	$2.99E-29\ m^3$
$C_{P_{drygel}}$	specific heat capacity of dry gel	$1500\ J/(kg\cdot K)$
ρ_{water}	density of water	$1000\ kg/m^3$
k_{water}	conductivity of water	$0.625\ W/(m\cdot K)$
$C_{P_{water}}$	specific heat capacity of water	$4187\ J/(kg\cdot K)$
k_B	Boltzmann constant	$1.3806E-23\ J/K$
T	initial and surrounding temperature	20 ℃
D_{light}	Diameter of laser beam	$1mm$
P_{light_out}	power of laser under outside illumination	$0.3\ W$
P_{light_in1}	power of laser under inside illumination with one bending point	$0.3\ W$
P_{light_in2}	power of laser under inside illumination with two bending points	$0.5\ W$
θ_{out}	incident direction of outside illumination	27°
θ_{in}	incident direction of inside illumination	10°

Table S4. Parameters used in the simulation for five port elements.

Parameters	Descriptions	Values
D_{out}	outer diameter of cladding	$2\ mm$
D_{in}	inner diameter of cladding	$1\ mm$
H	height of cladding	$16\ mm$
E_{gel}	Young's modulus of dry gel	$100\ MPa$
υ_{gel}	Poisson's ratio of dry gel	0.5
ν	molecule volume of water	$2.99E-29\ m^3$
$C_{P_{drygel}}$	specific heat capacity of dry gel	$1500\ J/(kg \cdot K)$
ρ_{water}	density of water	$1000\ kg/m^3$
k_{water}	conductivity of water	$0.625\ W/(m \cdot K)$
$C_{P_{water}}$	specific heat capacity of water	$4187\ J/(kg \cdot K)$
P_{light}	power of laser	$0.1\ W$
D_{light}	Diameter of laser beam	$1mm$
k_B	Boltzmann constant	$1.3806E-23\ J/K$
T	initial and surrounding temperature	20 ℃

Table S5. Parameters used in the simulation for light propagation under internal illumination.

Parameters	Descriptions	Values
R_out	outer radius of cladding	$1\ mm$
R_in	inner radius of cladding	$0.5\ mm$
L	Length of the sample	$16\ mm$
rho	radius curvature of the bending part	$1.1\ mm$
$theta$	bending angle	10 °
$n1$	refractive index of the outer layer	1.37
$n2$	refractive index of the inner layer	1.42
$n0$	refractive index of external environment	1.33

Table S6. Parameters used in the simulation for light propagation under external illumination.

Parameters	Descriptions	Values
R_out	outer radius of cladding	$1\ mm$
R_in	inner radius of cladding	$0.5\ mm$
L	Length of the sample	$16\ mm$
rho	radius curvature of the bending part	$1.1\ mm$
$theta$	bending angle	$15.5\ °$
$n1$	refractive index of the outer layer	1.37
$n2$	refractive index of the inner layer	1.42
$n0$	refractive index of external environment	1.33
D_light	diameter of the incident light	$0.2\sim2\ mm$

Table S7 Young's modulus and toughness of hydrogels and LCEs

Sample	Young's modulus (kPa)	Toughness (MPa)
PNIPAAm hydrogel without allyl disulfide	144	7.23
PNIPAAm hydrogel with allyl disulfide	76	1.72
RM257 LCE	684	304

References:

1. Hong W., Zhao X., Zhou J., et al. (2008). A theory of coupled diffusion and large deformation in polymeric gels. *Journal of the Mechanics and Physics of Solids* **56**:1779-1793. DOI:10.1016/j.jmps.2007.11.010
2. Duan Z., Zhang J., An Y., et al. (2013). Simulation of the transient behavior of gels based on an analogy between diffusion and heat transfer. *Journal of Applied Mechanics-Transactions of the Asme* **80**:041017. 041017,DOI:10.1115/1.4007789
3. Qian X., Zhao Y., Alsaid Y., et al. (2019). Artificial phototropism for omnidirectional tracking and harvesting of light. *Nature Nanotechnology* **14**:1048-1055. DOI:10.1038/s41565-019-0562-3
4. Hou G., Zhang X., Du F., et al. (2024). Self-regulated underwater phototaxis of a photoresponsive hydrogel-based phototactic vehicle. *Nature Nanotechnology* **19**:77-84. DOI:10.1038/s41565-023-01490-4
5. Xin C., Ren Z., Zhang L., et al. (2023). Light-triggered multi-joint microactuator fabricated by two-in-one femtosecond laser writing. *Nature Communications* **14**:4273. DOI:10.1038/s41467-023-40038-x
6. Zhao Y., Li Q., Liu Z., et al. (2023). Sunlight-powered self-excited oscillators for sustainable autonomous soft robotics. *Science Robotics* **8**:eadf4753. eadf4753,DOI:10.1126/scirobotics.adf4753
7. Zong Y., Xi M., Wang Y., et al. (2025). Waveguide Microactuators Self-Rolled Around an Optical Fiber Taper. *Advanced Materials* **37**. DOI:10.1002/adma.202418316
8. Tang J., Zhang F., Zhou F., et al. (2020). Broadband nonlinear optical response in GeSe nanoplates and its applications in all-optical diode. *Nanophotonics* **9**:2007-2015. DOI:10.1515/nanoph-2019-0531
9. Fan L., Wang J., Varghese L. T., et al. (2012). An all-silicon passive optical diode. *Science* **335**:447-450. DOI:10.1126/science.1214383
10. Bi L., Hu J., Jiang P., et al. (2011). On-chip optical isolation in monolithically integrated non-reciprocal optical resonators. *Nature Photonics* **5**:758-762. DOI:10.1038/nphoton.2011.270
11. Shoji Y., Mizumoto T., Yokoi H., et al. (2008). Magneto-optical isolator with silicon waveguides fabricated by direct bonding. *Applied Physics Letters* **92**:071117. 071117,DOI:10.1063/1.2884855
12. Yan W., Wei Z., Yang Y., et al. (2024). Ultra-broadband magneto-optical isolators and circulators on a silicon nitride photonics platform. *Optica* **11**:376-384. DOI:10.1364/optica.506366
13. Yu M., Cheng R., Reimer C., et al. (2023). Integrated electro-optic isolator on thin-film lithium niobate. *Nature Photonics* **17**:666-671. DOI:10.1038/s41566-023-01227-8
14. Kittlaus E. A., Otterstrom N. T., Kharel P., et al. (2018). Non-reciprocal interband Brillouin modulation. *Nature Photonics* **12**:613-619. DOI:10.1038/s41566-018-0254-9
